# The causal relationship between immune cell-mediated gut microbiota and ulcerative colitis: a bidirectional two-sample, mediation Mendelian randomization analysis

**DOI:** 10.3389/fnut.2024.1433545

**Published:** 2024-10-25

**Authors:** Jinyin Xiao, Xiajun Guo, Youwei Lin, Zhenquan Wang

**Affiliations:** ^1^Department of Anorectal, The Second Affiliated Hospital of Hunan University of Traditional Chinese Medicine, Changsha, China; ^2^Graduate School, Hunan University of Traditional Chinese Medicine, Changsha, China; ^3^Department of Geriatric, The First People’s Hospital of Xiangtan City, Xiangtan, China

**Keywords:** gut microbiota, immune cells, ulcerative colitis, mediation analysis, Mendelian randomization

## Abstract

**Background:**

Numerous studies have highlighted the close association between gut microbiota and the development of ulcerative colitis (UC), yet research on whether immune cells mediate this process remains scarce. This study utilizes various Mendelian randomization (MR) methods to investigate the causal relationship between gut microbiota and UC, further exploring the mediating role of immune cells in this process.

**Methods:**

Genome-wide association study (GWAS) summary statistics for 473 gut microbiota, 731 immune cell phenotypes, and UC were obtained from the GWAS catalog database. Single nucleotide polymorphisms (SNP) were used as instrumental variables (IV) to validate the causal relationship between gut microbiota and UC through two-sample MR and Bayesian weighted MR (BWMR), and reverse MR was employed to explore the presence of reverse causal effects. Two-step MR was applied to identify immune cell mediators and evaluate their mediation effects.

**Results:**

The study revealed a causal relationship between 20 gut microbiota and UC, with 14 microbiota acting as protective factors for UC and 6 as risk factors. Mediation MR identified 26 immune cell mediators, among which the association between CD11b on Mo MDSC and *Bifidobacterium bifidum* (*B. bifidum*) was most significant (*p* = 0.0017, OR = 1.4540, 95% CI: 1.1504–1.8378). Mediation MR analysis indicated that the mediation effect of CD11b on Mo MDSC between *B. bifidum* and UC was −0.0385, with a mediation effect ratio of 16.67%.

**Conclusion:**

There is a clear causal relationship between certain gut microbiota and UC, and CD11b on Mo MDSC is a significant mediator between *B. bifidum* and UC, providing new insights for the clinical treatment of UC.

## 1 Introduction

Ulcerative colitis (UC), a principal form of inflammatory bowel disease (IBD), is characterized by continuous mucosal inflammation and ulceration in the colon and rectum (1). The global incidence of UC is rapidly increasing, marking it as a significant global disease of the 21st century (2). Although the pathogenesis of UC is not fully understood, dysbiosis of gut microbiota and immune-mediated inflammatory responses are considered closely related to its occurrence and development, and there is a complex interaction between them (3).

The gut microbiota, one of the most complex microbial communities within the human body, consists primarily of bacteria, archaea, fungi, and viruses, with bacteria being the most abundant (4). These microorganisms have profound effects on host health and disease. Under physiological conditions, the gut microbiota exists in a symbiotic relationship with the host, participating in body metabolism, promoting nutrient absorption, providing energy support, regulating intestinal immunity, inhibiting pathogens, and maintaining gut homeostasis (5). However, compared to healthy individuals, a significant alteration in the structure, diversity, and function of the gut microbiota has been observed in a large number of IBD patients. These changes primarily manifest as a decrease in specific beneficial bacteria, such as Bifidobacterium and Lactobacillus, and an increase in the abundance of potentially pathogenic bacteria, including *Escherichia coli*, Fusobacteriaceae, and *Enterococcus faecalis*, with these microbial shifts being considered pivotal factors in the development of UC (3, 6, 7). Consequently, fecal microbiota transplantation (FMT) is considered a potential therapeutic strategy for UC (8).

Notably, the gut microbiota plays a crucial role in regulating the balance and function of the intestinal immune system. On one hand, the gut microbiota promotes the development and maturation of the intestinal mucosal immune system, thereby maintaining intestinal barrier function and homeostasis (9). On the other hand, the gut microbiota and their associated metabolites stimulate the release of downstream signaling molecules by intestinal epithelial cells and immune cells, modulating the activation status and inflammatory responses of immune cells, thereby influencing the immune balance of the intestinal mucosa (10, 11). Consequently, abnormalities in the gut microbiota can lead to dysregulation and abnormal activation of the intestinal immune system, triggering aberrant responses of immune cells, which may initiate and exacerbate inflammatory responses and tissue damage, potentially constituting an important mechanism in the pathogenesis of UC (12, 13). In recent years, there has been a surge of interest in the interplay between the gut microbiota and intestinal immunity in the context of UC, leading to a proliferation of related research. However, the majority of these studies have primarily focused on the interactions between alterations in the overall structure and diversity of the gut microbiota and the dysregulation of the intestinal immune system. Comparatively, fewer studies have delved deeply into or specifically examined the interactions between individual species of gut microbiota and particular types of immune cells.

Mendelian randomization (MR) is a novel epidemiological approach that utilizes genetic variations as instrumental variables (IV) based on genome-wide association study (GWAS) data to assess the causal relationship between exposure factors and outcomes (14). Similar to randomized controlled trials (RCT), MR analysis can effectively reduce the interference of confounding factors, providing evidence closer to the true causal relationship and ensuring the authenticity and reliability of the results (15). However, unlike RCT, MR studies have the advantages of being simple to conduct, requiring less time and cost, and effectively addressing ethical and reverse causality issues (15). Therefore, this study employs various MR methods to explore the causal relationship between the gut microbiota and UC, and further investigates whether immune cells mediate this process and the potential mediating factors involving immune cells.

## 2 Materials and methods

### 2.1 Study design

**Figure 1:**
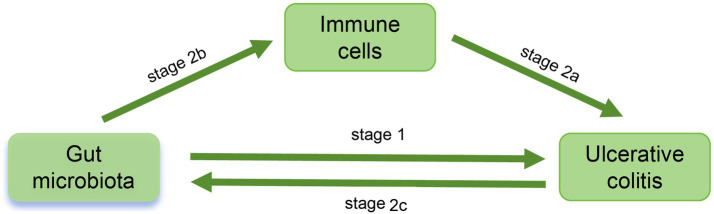
Study Workflow. In stage 1, the causal relationship between 473 gut microflora and UC was analyzed. In stage 2, the potential mediators and mediation effects of 731 immune cell phenotypes between intestinal flora and UC were investigated: in stage 2a, explore the effect of immune cells on UC; in stage 2b, explore the influence of intestinal flora on immune cells and determine the potential mediators; in stage 2c, explore whether there is a reverse causal relationship between UC and intestinal flora.

This study employed single nucleotide polymorphisms (SNP) as IV to investigate the causal relationship between 473 gut microbiota species and UC, as well as potential immune mediation, using various MR analysis methods. The study was primarily divided into two stages (Figure 1). In the first stage, we utilized two-sample MR and Bayesian weighted MR (BWMR) to establish the causal relationship between gut microbiota and UC. In the second stage, we further screened potential mediators and their mediation effects that mediated the causal relationship between gut microbiota and UC from 731 immune cell phenotypes through mediation MR analysis.

### 2.2 Data sources for gut microbiota, immune cells and UC

The gut microbiota data were sourced from a study conducted by Qin et al. (16), which reported the presence of 473 gut microbiota species in the feces of 5,959 Finnish individuals. The summary statistics mentioned were obtained from the GWAS catalog (GCST90032172-GCST90032644).^1^

The immune cell data were derived from a study conducted by Valeria Orru et al. (17) that included 731 immune cell phenotypes in 3757 European Sardinia populations. These phenotypes included absolute cell counts (AC), relative cell counts (RC), median fluorescence intensity (MFI) reflecting surface antigen levels, and morphological parameters (MP), and the above immune cell phenotypes included TBNK, Treg, T-cell, dendritic cells, T-cell, monocytes, and myeloid cell panel. The GWAS summary statistics for the above 731 immune cell phenotypes were publicly available from GWAS catalog (GCST90001391-GCST90002121).

The summary statistics related to UC were obtained from the GWAS catalog (GCST90044155). This dataset comprised 2,569 cases of European ancestry and 453,779 controls of the same descent, encompassing 11,842,647 SNPs (18). In this study, GWAS data for the gut microbiome and immune cells are continuous variables, whereas UC GWAS data are binary variables.

### 2.3 Selection of IV

For MR studies, the selection of IVs must satisfy the following assumptions: (1) a significant correlation exists between the IV and gut microbiota; (2) the IV is independent of any confounders; (3) there is no direct association between the IV and UC, with the relationship mediated solely through the gut microbiota. Furthermore, to ensure the accuracy of mediation MR analysis and the validity of causal inference, the following assumptions must be met: (1) The IV must be significantly associated with the exposure variable. (2) The relationship between the IV and the outcome variable should be mediated solely through the exposure variable (and the mediator), without other confounding pathways. (3) The IV should influence the outcome variable only through the exposure variable (and the mediator influenced by the exposure variable), without other direct paths. (4) The effect of the exposure variable on the outcome variable should be fully mediated by the mediator, with no unmeasured confounding factors affecting the relationship between the mediator and the outcome variable. Specific criteria for selecting SNP within the gut microbiome include: (1) identifying SNPs with genome-wide significance using *p* < 5 × 10^−8. If the number of SNPs in the exposure data is below the minimum required for MR studies (10 SNPs), a more lenient criterion of *p* < 1 × 10^−5 is used; (2) removing linkage disequilibrium in SNP with parameters set at *r*^2^ = 0.001 and kb = 10,000 to minimize the effect of confounders and enhance accuracy of the results; (3) excluding weak instrumental variables using an F-statistic <10 to prevent bias in causal inference.

### 2.4 Statistical analysis

All MR and statistical analyses involved in this study were conducted using the “TwoSampleMR” package (version 0.5.11) in R software version 4.3.3. During the MR analysis, we used Inverse Variance Weighted (IVW) as the main analysis method, and other supplementary analytical approaches included MR Egger, Weighted Median, Simple Mode, and Weighted Mode. Sensitivity analyses were conducted employing Cochran’s Q statistic and MR-Egger regression to test for heterogeneity among SNP (*p* < 0.05 indicating heterogeneity), and MR-Egger regression to detect horizontal pleiotropy (*p* < 0.05 indicating pleiotropy). Leave-one-out sensitivity analysis was employed to assess whether any single SNP disproportionately influenced the causal relationship between gut microbiota and UC (19). Statistical power was calculated using the online tool developed by Stephen Burgess.^2^ In mediation analysis, a two-step MR approach was used to evaluate the mediating effects of immune cells between gut microbiota and UC (20).

## 3 Results

### 3.1 Impact of gut microbiota on UC

**Figure 2:**
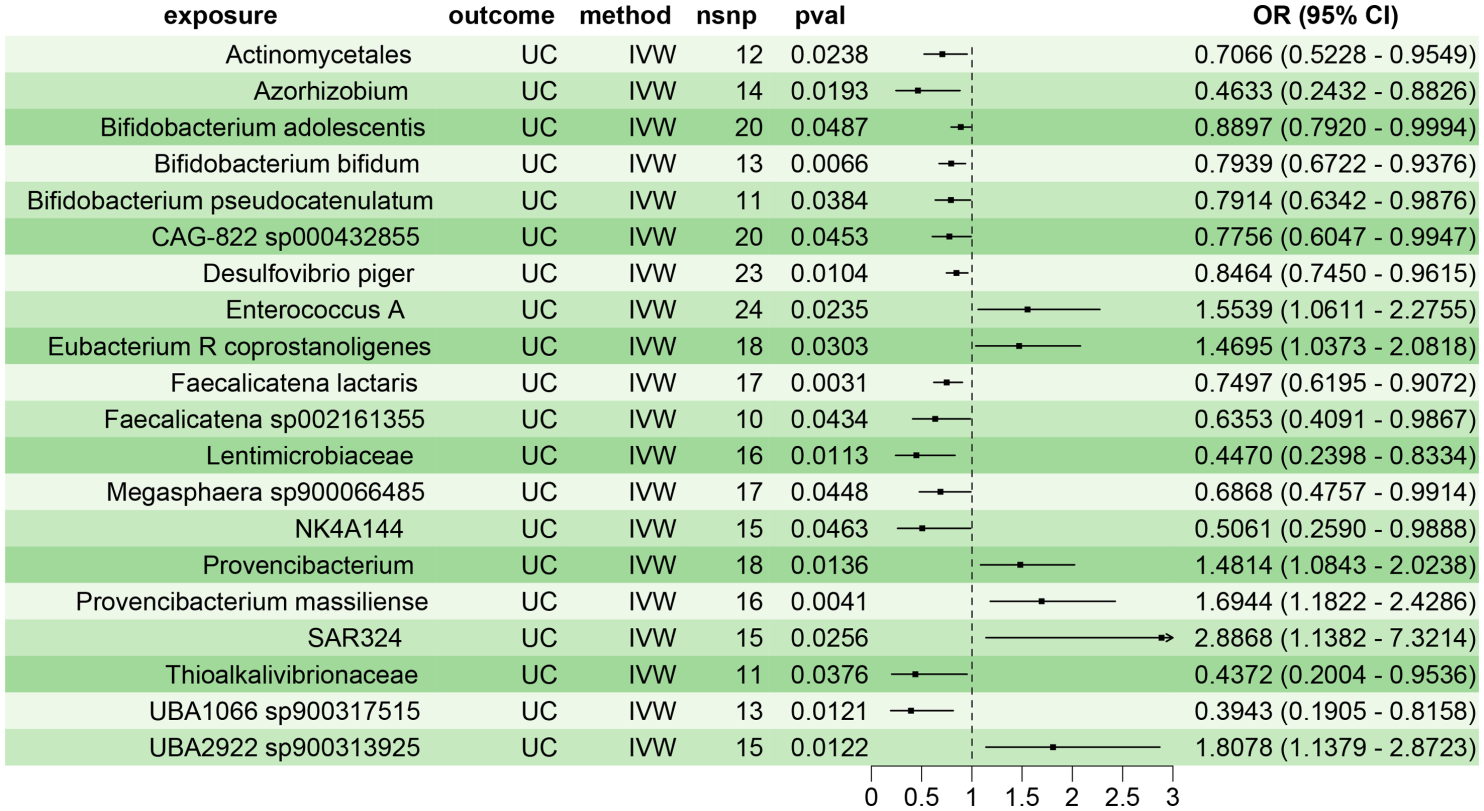
Two-sample MR assessing the causal relationship between gut microbiota and UC.

Using the two-sample MR, we assessed the causal relationship between 473 gut microbiota species and UC. We identified 20 gut microbiota species with significant causal associations with UC (*p* < 0.05), of which 14 were protective factors for UC (OR < 1), including Actinomycetales (OR = 0.7066, 95% CI: 0.5228–0.9549), Azorhizobium (OR = 0.4633, 95% CI: 0.2432–0.8826), *Bifidobacterium adolescentis* (OR = 0.8897, 95% CI: 0.7920–0.9994), *Bifidobacterium bifidum* (*B. bifidum*) (OR = 0.7939, 95% CI: 0.6722–0.9376), *Bifidobacterium pseudocatenulatum* (OR = 0.7914, 95% CI: 0.6342–0.9876), CAG-822 sp000432855 (OR = 0.7756, 95% CI: 0.6047–0.9947), *Desulfovibrio piger* (OR = 0.8464, 95% CI: 0.7450–0.9615), Faecalicatena lactaris (OR = 0.7497, 95% CI: 0.6195–0.9072), Faecalicatena sp002161355 (OR = 0.6353, 95% CI: 0.4091–0.9867), Lentimicrobiaceae (OR = 0.4470, 95% CI: 0.2398–0.8334), Megasphaera sp900066485 (OR = 0.6868, 95% CI: 0.4757–0.9914), NK4A144 (OR = 0.5061, 95% CI: 0.2590–0.9888), Thioalkalivibrionaceae (OR = 0.4372, 95% CI: 0.2004–0.9536), and UBA1066 sp900317515 (OR = 0.3943, 95% CI: 0.1905–0.8158). Six gut microbiota species were identified as risk factors for UC (OR > 1), including Enterococcus A (OR = 1.5539, 95% CI: 1.0611–2.2755), Eubacterium R coprostanoligenes (OR = 1.4695, 95% CI: 1.0373–2.0818), Provencibacterium (OR = 1.4814, 95% CI: 1.0843–2.0238), Provencibacterium massiliense (OR = 1.6944, 95% CI: 1.1822–2.4286), SAR324 (OR = 2.8868, 95% CI: 1.1382–7.3214), and UBA2922 sp900313925 (OR = 1.8078, 95% CI: 1.1379–2.8723) (Figure 2). Sensitivity analyses revealed no significant heterogeneity or pleiotropy in the above results, indicating the reliability and robustness of the study findings (Supplementary Table 2; Supplementary Figure File 1).

**Figure 3:**
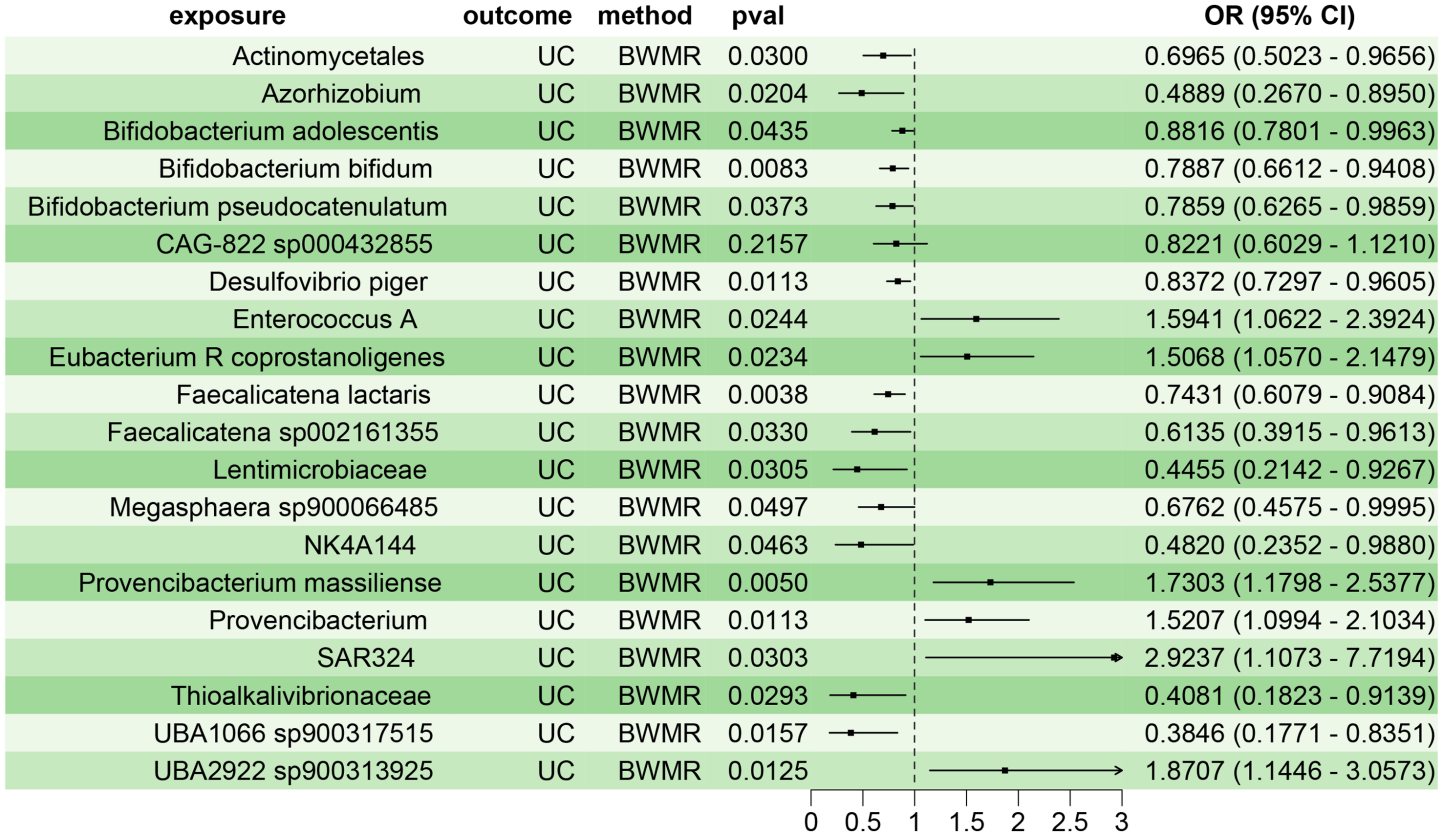
BWMR assessing the causal relationship between gut microbiota and UC.

To confirm the reliability of the aforementioned findings, we further validated the causal relationship between the 20 gut microbiota species and UC using BWMR approach. The results demonstrated a clear causal relationship between these 20 gut microbiota species and UC, with 14 species acting as protective factors and 6 species as risk factors for UC (Figure 3). This corroborated the findings from the two-sample MR analysis, further substantiating the reliability of the results.

### 3.2 Immune cells mediating the impact of gut microbiota on UC

Initially, we screened 34 immune cell phenotypes associated with UC from 731 using MR analysis (Supplementary Figure 1). Subsequently, we explored the relationship between the 20 gut microbiota closely related to UC and the 34 immune cell phenotypes. Our findings revealed that 13 gut microbiota were significantly associated with 26 immune cell phenotypes (*p* < 0.05) (Supplementary Figure 2). Notably, *B. bifidum* and the immune cell CD11b on Mo MDSC exhibited the most significant positive correlation (*p* = 0.0017, OR = 1.4540, 95% CI: 1.1504–1.8378) in the IVW test. Consequently, we selected *B. bifidum* as the exposure factor and CD11b on Mo MDSC as the mediator to analyze the mediating effect of CD11b on Mo MDSC in the causal relationship between *B. bifidum* and UC.

**Figure 4:**
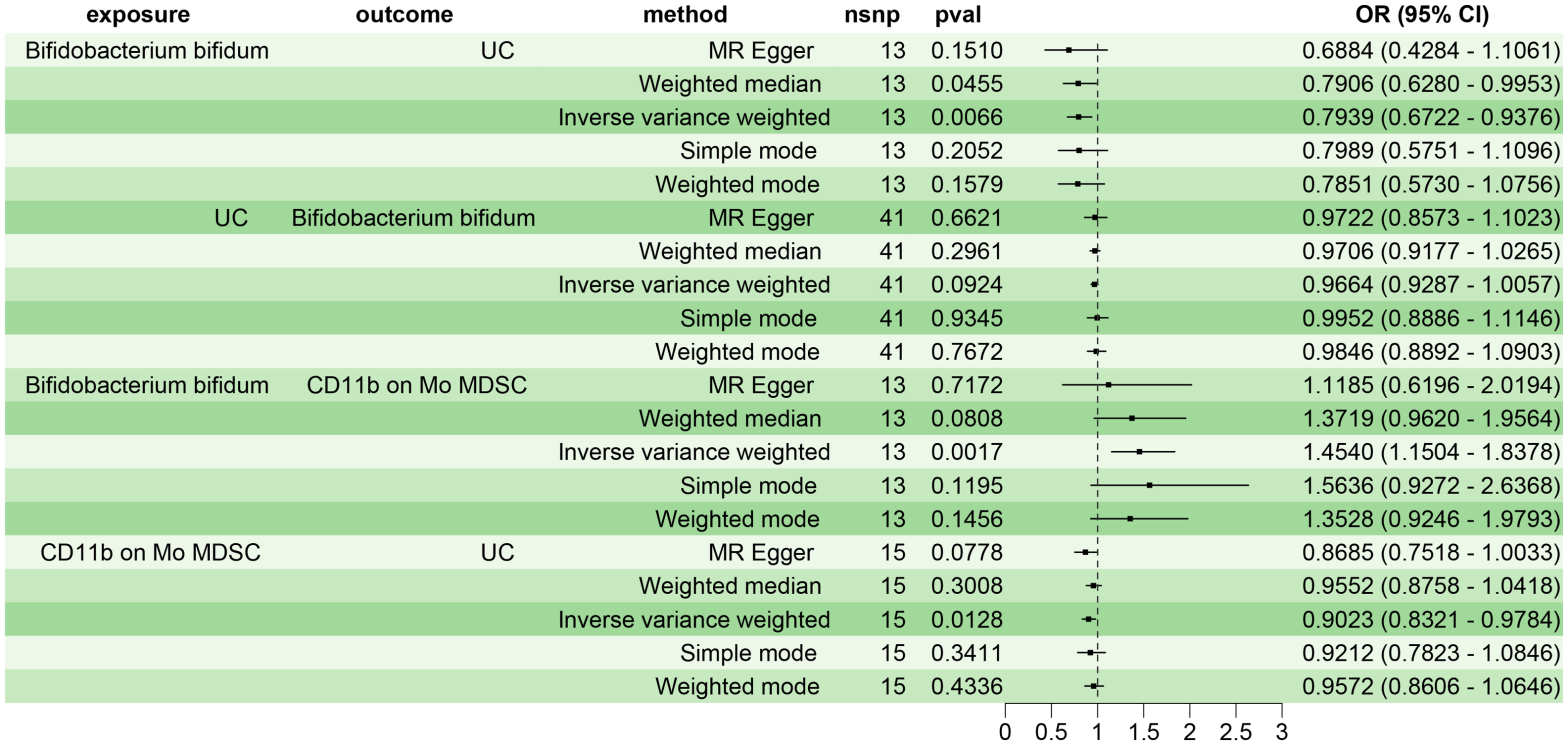
Impact of *B. bifidum* on UC mediated by CD11b on Mo MDSC.

Prior to conducting the mediating MR analysis, we needed to exclude the reverse causal effect of UC on *B. bifidum* through reverse MR analysis. The results of the reverse MR analysis indicated no reverse causal effect between UC and *B. bifidum* (*p* = 0.0924) (Figure 4). Consequently, we employed a two-step MR analysis to investigate the mediating effect of CD11b on Mo MDSC in the causal relationship between *B. bifidum* and UC. The results revealed a significant causal relationship between *B. bifidum* and UC, with *B. bifidum* acting as a protective factor (*p* = 0.0066, OR = 0.7939, 95% CI: 0.6722–0.9376). *B. bifidum* was positively correlated with CD11b on Mo MDSC (*p* = 0.0017, OR = 1.4540, 95% CI: 1.1504–1.8378), while CD11b on Mo MDSC was negatively correlated with UC (*p* = 0.0128, OR = 0.9023, 95% CI: 0.8321–0.9784) (Figure 4). β1 = 0.3743, β2 = −0.1028, where β1 represents the effect of the exposure factor (*B. bifidum*) on the mediator (CD11b on Mo MDSC), and β2 represents the effect of the mediator (CD11b on Mo MDSC) on the outcome (UC). The total effect of *B. bifidum* on UC was −0.2308, the direct effect was −0.1923, and the mediating effect of CD11b on Mo MDSC in the relationship between *B. bifidum* and UC was −0.0385, with a mediating effect ratio of 16.67% (Supplementary Table 3).

## 4 Discussion

This study identified 20 gut microbiota with significant causal associations with UC from 473 gut microbiota, using Two-sample MR and BWMR. A two-step MR was employed to examine the effects of gut microbiota on immune cells and the influence of immune cells on UC. The results revealed 26 immune cell phenotypes potentially mediating the causal relationship between gut microbiota and UC, with the link between CD11b on Mo MDSC and *B. bifidum* being notably prominent. The findings indicate that *B. bifidum*’s protective regulation of UC is partly mediated by CD11b on Mo MDSC (16.67%), underscoring the causal connection between *B. bifidum* and UC and highlighting the intermediary role of CD11b on Mo MDSC.

Bifidobacterium, one of the most critical probiotics in the gut, includes *B. bifidum*, *B. adolescentis*, and *B. longum*, widely used in the clinic to treat various gastrointestinal diseases (21). Studies indicate that Bifidobacterium improves IBD by maintaining intestinal barrier function, regulating gut immunity and microbiome diversity (22, 23). *B. bifidum*, a crucial type of Bifidobacterium, is found to alleviate UC via several pathways. Firstly, *B. bifidum* strengthens cell tight junctions and maintains intestinal epithelial barrier function to alleviate UC. For instance, through its derived proteins, *B. bifidum* regulates nuclear factor kappa-B (NF-κB), RhoA/Rho associated kinase (ROCK), and mitogen-activated protein kinase (MAPK) signaling pathways, enhancing the expression of tight junction proteins and mitigating inflammation in DSS-induced UC mice (24). In DSS-induced colitis, *B. bifidum* metabolite indole-3-lactic acid regulates aryl hydrocarbon receptor (AHR)/NF-E2-related factor 2 (NRF2)/NOD-like receptor thermal protein domain associated protein 3 (NLRP3) signaling pathway, thereby upregulating tight junction proteins and protecting intestinal epithelial barrier function (25). Ahmad et al. found that *B. bifidum* mitigated inflammation in UC mice by regulating multiple miRNAs, thereby adjusting the expression of tight junction and NF-κB proteins (26). Secondly, *B. bifidum* has immune-regulatory functions, inhibiting the expression of inflammatory factors interleukin (IL)-1β, tumor necrosis factor (TNF)-*α*, IL-6, and interferon (IFN)-*γ*, while promoting IL-10 expression, thus facilitating gut damage repair in colitis mice (27, 28). Lastly, the therapeutic mechanism of *B. bifidum* on UC may also relate to its ability to increase short-chain fatty acid production (29). Consistent with our results, these studies underscore the significant protective role of *B. bifidum* in UC. However, these are primarily animal studies, lacking support from other evidence layers. Conversely, our study verifies the causal relationship between the two from a new perspective.

Although studies have shown that *B. bifidum* has good immunomodulatory effects for UC, little is known about whether and which immune cells are involved in these processes. The concept of myeloid-derived suppressor cells (MDSCs) was formally defined in 2007 (30), primarily composed of immature myeloid progenitor cells, and precursors of macrophages, dendritic cells (DC) and granulocytes, representing a heterogeneous cell population with immune suppressive function (31). Under the stimulation of various pathological conditions such as cancer, infection, and chronic inflammation, MDSCs are generated and accumulated, suppressing the functions of T cells and other immune cells through various mechanisms, thereby maintaining immune balance and preventing overreaction (32, 33). CD11b on Mo MDSC refers to monocytic MDSCs expressing CD11b on the cell surface. As an important marker on the surface of MDSCs, CD11b, a member of the integrin family, is related to the migration, adhesion, and immune suppression ability of MDSCs (34). Mo MDSC is one of the main subgroups of MDSCs. Although there is no research evidence to directly confirm that it mediates the protective regulation of UC by *B. bifidum*, it has been found that chronic inflammation can activate and promote the accumulation of MDSCs in the colon, thus promoting the expression of IL-10, or inhibiting IFN- *γ* production by T cells, ultimately improving intestinal inflammation, and MDSCs are associated with the occurrence of colon cancer (35, 36). Moreover, cannabinoids receptor agonist SMM-189 and IL-37b genetically modified mouse bone marrow mesenchymal stem cells (MSC-IL-37b) inhibit colitis by increasing the number of MDSCs or inducing MDSCs differentiation, possibly related to the immune suppression function of MDSCs (37, 38). Consistent with these findings, our study also regards Mo MDSC as negatively correlated with UC, indicating the potential suppressive effect of Mo MDSC on UC.

Interestingly, studies have found that MDSCs alone cannot alleviate DSS-induced colitis, but in synergy with the gut microbiota metabolite butyrate, MDSCs can effectively suppress colonic inflammation (39). Although the associations between *B. bifidum* and UC, as well as MDSCs and UC have been well-recognized, it remains unclear whether MDSCs are involved in mediating the process by which *B. bifidum* regulates UC. Limited research has shown that *B. bifidum* significantly stimulates the expression of co-stimulatory molecules and anti-inflammatory cytokines (IL-10 and TGF-*β*) in DC of UC patients (40). Our findings suggest that Mo MDSC mediates the causal effect between *B. bifidum* and UC, providing new insights for the regulation of UC by gut microbiota.

In this study, we employed various MR analysis methods to explore the causal effects between 473 novel gut microbiota species and UC, as well as potential immune mediators on a large scale. We pioneered the proposition that Mo MDSC mediates the causal relationship between *B. bifidum* and UC, providing new strategies for the prevention and treatment of UC. Additionally, sensitivity analyses and reverse MR were conducted to exclude potential confounding factors and reverse causal effects, enhancing the reliability of our findings. However, there are some limitations to this study. Firstly, although we demonstrated the causal relationship between gut microbiota and UC using a large sample, we only included participants of European ancestry to avoid heterogeneity and bias caused by genetic differences among different ethnicities. Therefore, the results may not be representative of other populations. Future studies should validate these findings in other ethnic groups to assess their applicability and enhance the generalizability of the research. Secondly, due to the extensive workload, we did not fully discuss the potential mediating roles of other 25 immune cell phenotypes in this process. Thirdly, although our MR analysis initially confirmed the causal relationship between gut microbiota and UC, as well as the mediating role of immune cells, these conclusions have not yet been supported by animal or clinical evidence, which may be the focus of our future work. Fourthly, although this study used highly relevant IVs and multiple sensitivity analyses to reduce the impact of confounding factors, we acknowledge that it is still not possible to completely eliminate all potential confounders. Therefore, future research should employ additional methods to validate our findings. Lastly, since the immune cell data in this study were derived from peripheral blood, and immune cells in peripheral blood may significantly differ in expression levels, phenotypes, and functions from those in the gastrointestinal tract, the results may not fully explain gastrointestinal-specific immune responses. Future studies should consider directly collecting and analyzing immune cells from gastrointestinal tissues to achieve more accurate conclusions.

## 5 Conclusion

In summary, our study demonstrates a clear causal relationship between gut microbiota and UC, with certain immune cells mediating this process. Specifically, a higher level of *B. bifidum* is associated with a reduced risk of UC, and MDSCs play a significant mediating role in this context. This finding provides new targets and avenues for future therapeutic interventions using the gut microbiota to treat UC.

## Data Availability

The original contributions presented in the study are included in the article/Supplementary material, further inquiries can be directed to the corresponding author.

## Footnotes

^1^https://www.ebi.ac.uk/gwas

^2^https://sb452.shinyapps.io/power
