# Supplementary material for: The causal relationship between immune cell-mediated gut microbiota and ulcerative colitis: a bidirectional two-sample, mediation Mendelian randomization analysis
Source: Front Nutr. 2024 Oct 25;11:1433545. doi: 10.3389/fnut.2024.1433545 (PMC11545678; doi:10.3389/fnut.2024.1433545)
Supplement: Supplementary file 1 [file Data_Sheet_1.docx]

Supplementary Material

The causal relationship between immune cell-mediated gut microbiota and ulcerative colitis: a bidirectional two-sample, mediation Mendelian randomization analysis

Jinyin Xiao^1,2^, Xiajun Guo^3^, Youwei Lin^2^ and Zhenquan Wang^1*^

*** Correspondence:** Zhenquan Wang: [320035@hnucm.edu.cn](mailto:320035@hnucm.edu.cn)

# Supplementary Figures and Tables

## Supplementary Tables

**Supplementary Table 1.** STROBE-MR checklist of recommended items to address in reports of Mendelian randomization studies^1^ ^2^

| **Item No.** | **Section** | **Checklist item** | **Page No.** | **Relevant text from manuscript** |
| --- | --- | --- | --- | --- |
| 1 | **TITLE and ABSTRACT** | Indicate Mendelian randomization (MR) as the study’s design in the title and/or the abstract if that is a main purpose of the study |  | The title is “The causal relationship between immune cell-mediated gut microbiota and ulcerative colitis: a bidirectional two-sample, mediation Mendelian randomization analysis”. |
|  | **INTRODUCTION** |  |  |  |
| 2 | **Background** | Explain the scientific background and rationale for the reported study. What is the exposure? Is a potential causal relationship between exposure and outcome plausible? Justify why MR is a helpful method to address the study question |  | Numerous studies have highlighted the close association between gut microbiota and the development of ulcerative colitis (UC), yet research on whether immune cells mediate this process remains scarce. |
| 3 | **Objectives** | State specific objectives clearly, including pre-specified causal hypotheses (if any). State that MR is a method that, under specific assumptions, intends to estimate causal effects |  | This study utilizes various Mendelian randomization (MR) methods to investigate the causal relationship between gut microbiota and UC, further exploring the mediating role of immune cells in this process. |
|  | **METHODS** |  |  |  |
| 4 | **Study design and data sources** | Present key elements of the study design early in the article. Consider including a table listing sources of data for all phases of the study. For each data source contributing to the analysis, describe the following: |  |  |
|  | a) | Setting: Describe the study design and the underlying population, if possible. Describe the setting, locations, and relevant dates, including periods of recruitment, exposure, follow-up, and data collection, when available. |  | Study design : please see the section “2.1 Study design” and Figure 1.  Population: please see the section “2.2 Data sources for gut microbiota, immune cells and UC”. |
|  | b) | Participants: Give the eligibility criteria, and the sources and methods of selection of participants. Report the sample size, and whether any power or sample size calculations were carried out prior to the main analysis |  | Please see the section “2.2 Data sources for gut microbiota, immune cells”. |
|  | c) | Describe measurement, quality control and selection of genetic variants |  | Please see the section “2.3 Selection of IV”. |
|  | d) | For each exposure, outcome, and other relevant variables, describe methods of assessment and diagnostic criteria for diseases |  | Please see the section “2.2 Data sources for gut microbiota, immune cells”. |
|  | e) | Provide details of ethics committee approval and participant informed consent, if relevant |  | All participants provided informed consent in all the  corresponding original studies. All data used in this  work are publicly available from studies with  relevant participant consent and ethical approval.  Ethical approval from an institutional review board  was not necessary for the present study as only  publicly available summary level data was used. |
| 5 | **Assumptions** | Explicitly state the three core IV assumptions for the main analysis (relevance, independence and exclusion restriction) as well assumptions for any additional or sensitivity analysis |  | Please see the section “2.3 Selection of IV”. |
| 6 | **Statistical methods: main analysis** | Describe statistical methods and statistics used |  |  |
|  | a) | Describe how quantitative variables were handled in the analyses (i.e., scale, units, model) |  | NA |
|  | b) | Describe how genetic variants were handled in the analyses and, if applicable, how their weights were selected |  | NA |
|  | c) | Describe the MR estimator (e.g. two-stage least squares, Wald ratio) and related statistics. Detail the included covariates and, in case of two-sample MR, whether the same covariate set was used for adjustment in the two samples |  | Please see the section “2.3 Selection of IV”. |
|  | d) | Explain how missing data were addressed |  | NA |
|  | e) | If applicable, indicate how multiple testing was addressed |  | NA |
| 7 | **Assessment of assumptions** | Describe any methods or prior knowledge used to assess the assumptions or justify their validity |  | Please see the section “2.3 Selection of IV”. |
| 8 | **Sensitivity analyses and additional analyses** | Describe any sensitivity analyses or additional analyses performed (e.g. comparison of effect estimates from different approaches, independent replication, bias analytic techniques, validation of instruments, simulations) |  | Please see the section “2.4 Statistical analysis”. |
| 9 | **Software and pre-registration** |  |  |  |
|  | a) | Name statistical software and package(s), including version and settings used |  | All MR and statistical analyses involved in this study were conducted using the "TwoSampleMR" package (version 0.5.11) in R software version 4.3.3. |
|  | b) | State whether the study protocol and details were pre-registered (as well as when and where) |  | NA |
|  | **RESULTS** |  |  |  |
| 10 | **Descriptive data** |  |  |  |
|  | a) | Report the numbers of individuals at each stage of included studies and reasons for exclusion. Consider use of a flow diagram |  | Please see the section “2.2 Data sources for gut microbiota, immune cells and UC”. |
|  | b) | Report summary statistics for phenotypic exposure(s), outcome(s), and other relevant variables (e.g. means, SDs, proportions) |  | Please see the section “2.2 Data sources for gut microbiota, immune cells and UC”. |
|  | c) | If the data sources include meta-analyses of previous studies, provide the assessments of heterogeneity across these studies |  | NA |
|  | d) | For two-sample MR:  i.  Provide justification of the similarity of the genetic variant-exposure associations between the exposure and outcome samples  ii.  Provide information on the number of individuals who overlap between the exposure and outcome studies |  | Please see the section “2.4 Statistical analysis”.  Due to the use of summary-level statistics we were  not able to identify individuals who overlap between  exposure and outcome. |
| 11 | **Main results** |  |  |  |
|  | a) | Report the associations between genetic variant and exposure, and between genetic variant and outcome, preferably on an interpretable scale |  | Please see the section “2.3 Selection of IV”. |
|  | b) | Report MR estimates of the relationship between exposure and outcome, and the measures of uncertainty from the MR analysis, on an interpretable scale, such as odds ratio or relative risk per SD difference |  | Please see the section “2.3 Selection of IV”. |
|  | c) | If relevant, consider translating estimates of relative risk into absolute risk for a meaningful time period |  | NA |
|  | d) | Consider plots to visualize results (e.g. forest plot, scatterplot of associations between genetic variants and outcome versus between genetic variants and exposure) |  | Supplementary Figure File1. |
| 12 | **Assessment of assumptions** |  |  |  |
|  | a) | Report the assessment of the validity of the assumptions |  | Sensitivity analyses revealed no significant heterogeneity or pleiotropy in the above results, indicating the reliability and robustness of the study findings (Supplementary Table 2, Supplementary Figure File1). |
|  | b) | Report any additional statistics (e.g., assessments of heterogeneity across genetic variants, such as *I^2^*, Q statistic or E-value) |  | Sensitivity analyses revealed no significant heterogeneity or pleiotropy in the above results, indicating the reliability and robustness of the study findings (Supplementary Table 2, Supplementary Figure File1). |
| 13 | **Sensitivity analyses and additional analyses** |  |  |  |
|  | a) | Report any sensitivity analyses to assess the robustness of the main results to violations of the assumptions |  | Sensitivity analyses revealed no significant heterogeneity or pleiotropy in the above results, indicating the reliability and robustness of the study findings (Supplementary Table 2, Supplementary Figure File1). |
|  | b) | Report results from other sensitivity analyses or additional analyses |  | Sensitivity analyses revealed no significant heterogeneity or pleiotropy in the above results, indicating the reliability and robustness of the study findings (Supplementary Table 2, Supplementary Figure File1). |
|  | c) | Report any assessment of direction of causal relationship (e.g., bidirectional MR) |  | Prior to conducting the mediating MR analysis, we needed to exclude the reverse causal effect of UC on B. bifidum through reverse MR analysis. The results of the reverse MR analysis indicated no reverse causal effect between UC and B. bifidum (P=0.0924) (Figure 4). |
|  | d) | When relevant, report and compare with estimates from non-MR analyses |  | NA |
|  | e) | Consider additional plots to visualize results (e.g., leave-one-out analyses) |  | Supplementary Figure File1. |
|  | **DISCUSSION** |  |  |  |
| 14 | **Key results** | Summarize key results with reference to study objectives |  | This study identified 20 gut microbiota with significant causal associations with UC from 473 gut microbiota, using Two-sample MR and BWMR. A two-step MR was employed to examine the effects of gut microbiota on immune cells and the influence of immune cells on UC. The results revealed 26 immune cell phenotypes potentially mediating the causal relationship between gut microbiota and UC, with the link between CD11b on Mo MDSC and B. bifidum being notably prominent. The findings indicate that B. bifidum's protective regulation of UC is partly mediated by CD11b on Mo MDSC (16.67%), underscoring the causal connection between B. bifidum and UC and highlighting the intermediary role of CD11b on Mo MDSC. |
| 15 | **Limitations** | Discuss limitations of the study, taking into account the validity of the IV assumptions, other sources of potential bias, and imprecision. Discuss both direction and magnitude of any potential bias and any efforts to address them |  | However, there are some limitations to this study. Firstly, although we demonstrated the causal relationship between gut microbiota and UC using a large sample, we only included participants of European ancestry to avoid heterogeneity and bias caused by genetic differences among different ethnicities, thus the results may not be representative of other races. Secondly, due to the extensive workload, we did not fully discuss the potential mediating roles of other 25 immune cell phenotypes in this process. Lastly, although our MR analysis initially confirmed the causal relationship between gut microbiota and UC, as well as the mediating role of immune cells, these conclusions have not yet been supported by animal or clinical evidence, which may be the focus of our future work. |
| 16 | **Interpretation** |  |  |  |
|  | a) | Meaning: Give a cautious overall interpretation of results in the context of their limitations and in comparison with other studies |  | In this study, we employed various MR analysis methods to explore the causal effects between 473 novel gut microbiota species and UC, as well as potential immune mediators on a large scale. We pioneered the proposition that Mo MDSC mediates the causal relationship between B. bifidum and UC, providing new strategies for the prevention and treatment of UC. Additionally, sensitivity analyses and reverse MR were conducted to exclude potential confounding factors and reverse causal effects, enhancing the reliability of our findings. |
|  | b) | Mechanism: Discuss underlying biological mechanisms that could drive a potential causal relationship between the investigated exposure and the outcome, and whether the gene-environment equivalence assumption is reasonable. Use causal language carefully, clarifying that IV estimates may provide causal effects only under certain assumptions |  | Please refer to the section "Discussion". |
|  | c) | Clinical relevance: Discuss whether the results have clinical or public policy relevance, and to what extent they inform effect sizes of possible interventions |  | Please refer to the section "Discussion". |
| 17 | **Generalizability** | Discuss the generalizability of the study results (a) to other populations, (b) across other exposure periods/timings, and (c) across other levels of exposure |  | The conclusions drawn from our data could not be  immediately generalized to other populations, as all  of our data originates from European sources. |
|  | **OTHER INFORMATION** |  |  |  |
| 18 | **Funding** | Describe sources of funding and the role of funders in the present study and, if applicable, sources of funding for the databases and original study or studies on which the present study is based |  | The author(s) declare financial support was received for the research, authorship, and/or publication of this article. This research was funded by the Hunan Provincial Natural Science Foundation of China (2023JJ60040), Changsha City Natural Science Foundation (kq2202482), the Academic Leader of Hunan Chinese Medicine Anorectal Disease (Hunan Chinese Medicine Letter [2022] No.4), and the Hunan Clinical Research Center for the Prevention and Treatment of Anorectal Diseases (Hunan TCM Letter [2022] No.93). |
| 19 | **Data and data sharing** | Provide the data used to perform all analyses or report where and how the data can be accessed, and reference these sources in the article. Provide the statistical code needed to reproduce the results in the article, or report whether the code is publicly accessible and if so, where |  | The original contributions presented in the study are included in the article/Supplementary material, further inquiries can be directed to the corresponding authors. |
| 20 | **Conflicts of Interest** | All authors should declare all potential conflicts of interest |  | The authors declare that the research was conducted in the absence of any commercial or financial relationships that could be construed as a potential conflict of interest. |

This checklist is copyrighted by the Equator Network under the Creative Commons Attribution 3.0 Unported (CC BY 3.0) license.

1. Skrivankova VW, Richmond RC, Woolf BAR, Yarmolinsky J, Davies NM, Swanson SA, et al. Strengthening the Reporting of Observational Studies in Epidemiology using Mendelian Randomization (STROBE-MR) Statement. JAMA. 2021;under review.

2. Skrivankova VW, Richmond RC, Woolf BAR, Davies NM, Swanson SA, VanderWeele TJ, et al. Strengthening the Reporting of Observational Studies in Epidemiology using Mendelian Randomisation (STROBE-MR): Explanation and Elaboration. BMJ. 2021;375:n2233.

### Supplementary Table 2. Sensitivity analysis between 20 intestinal flora and UC.

| **exposure** | **outcome** | **method** | **pval** |
| --- | --- | --- | --- |
| Actinomycetales | UC | heterogeneity/MR Egger | 0.2940 |
|  |  | heterogeneity/IVW | 0.2708 |
|  |  | pleiotropy | 0.2892 |
| Azorhizobium | UC | heterogeneity/MR Egger | 0.7982 |
|  |  | heterogeneity/IVW | 0.8544 |
|  |  | pleiotropy | 0.9762 |
| Bifidobacterium adolescentis | UC | heterogeneity/MR Egger | 0.9609 |
|  |  | heterogeneity/IVW | 0.9735 |
|  |  | pleiotropy | 0.8225 |
| Bifidobacterium bifidum | UC | heterogeneity/MR Egger | 0.8180 |
|  |  | heterogeneity/IVW | 0.8470 |
|  |  | pleiotropy | 0.5420 |
| Bifidobacterium pseudocatenulatum | UC | heterogeneity/MR Egger | 0.1379 |
|  |  | heterogeneity/IVW | 0.1600 |
|  |  | pleiotropy | 0.5096 |
| CAG-822 sp000432855 | UC | heterogeneity/MR Egger | 0.4473 |
|  |  | heterogeneity/IVW | 0.5021 |
|  |  | pleiotropy | 0.6790 |
| Desulfovibrio piger | UC | heterogeneity/MR Egger | 0.8867 |
|  |  | heterogeneity/IVW | 0.8989 |
|  |  | pleiotropy | 0.4937 |
| Enterococcus A | UC | heterogeneity/MR Egger | 0.7140 |
|  |  | heterogeneity/IVW | 0.7525 |
|  |  | pleiotropy | 0.6380 |
| Eubacterium R coprostanoligenes | UC | heterogeneity/MR Egger | 0.2909 |
|  |  | heterogeneity/IVW | 0.3335 |
|  |  | pleiotropy | 0.6001 |
| Faecalicatena lactaris | UC | heterogeneity/MR Egger | 0.9272 |
|  |  | heterogeneity/IVW | 0.9513 |
|  |  | pleiotropy | 0.9599 |
| Faecalicatena sp002161355 | UC | heterogeneity/MR Egger | 0.1290 |
|  |  | heterogeneity/IVW | 0.1848 |
|  |  | pleiotropy | 0.9656 |
| Lentimicrobiaceae | UC | heterogeneity/MR Egger | 0.4005 |
|  |  | heterogeneity/IVW | 0.4702 |
|  |  | pleiotropy | 0.8083 |
| Megasphaera sp900066485 | UC | heterogeneity/MR Egger | 0.5550 |
|  |  | heterogeneity/IVW | 0.6269 |
|  |  | pleiotropy | 0.9306 |
| NK4A144 | UC | heterogeneity/MR Egger | 0.5406 |
|  |  | heterogeneity/IVW | 0.5913 |
|  |  | pleiotropy | 0.5671 |
| Provencibacterium massiliense | UC | heterogeneity/MR Egger | 0.4017 |
|  |  | heterogeneity/IVW | 0.4676 |
|  |  | pleiotropy | 0.7446 |
| Provencibacterium | UC | heterogeneity/MR Egger | 0.3092 |
|  |  | heterogeneity/IVW | 0.3714 |
|  |  | pleiotropy | 0.8828 |
| SAR324 | UC | heterogeneity/MR Egger | 0.9527 |
|  |  | heterogeneity/IVW | 0.9710 |
|  |  | pleiotropy | 0.9833 |
| Thioalkalivibrionaceae | UC | heterogeneity/MR Egger | 0.2626 |
|  |  | heterogeneity/IVW | 0.3425 |
|  |  | pleiotropy | 0.9799 |
| UBA1066 sp900317515 | UC | heterogeneity/MR Egger | 0.8459 |
|  |  | heterogeneity/IVW | 0.8922 |
|  |  | pleiotropy | 0.8306 |
| UBA2922 sp900313925 | UC | heterogeneity/MR Egger | 0.7006 |
|  |  | heterogeneity/IVW | 0.6800 |
|  |  | pleiotropy | 0.3016 |

### Supplementary Table 3. The mediating effect of CD11b on Mo MDSC in the causal relationship between B. bifidum and UC.

| **exposure** | **outcome** | **method** | **nsnp** | **b** | **se** | **pval** | **lo_ci** | **up_ci** | **or** | **or_lci95** | **or_uci95** |
| --- | --- | --- | --- | --- | --- | --- | --- | --- | --- | --- | --- |
| B. bifidum | UC | MR Egger | 13 | -0.3734 | 0.2420 | 0.1510 | -0.8476 | 0.1008 | 0.6884 | 0.4284 | 1.1061 |
|  |  | Weighted median | 13 | -0.2350 | 0.1131 | 0.0378 | -0.4567 | -0.0133 | 0.7906 | 0.6334 | 0.9868 |
|  |  | Inverse variance weighted | 13 | -0.2308 | 0.0849 | 0.0066 | -0.3973 | -0.0644 | 0.7939 | 0.6722 | 0.9376 |
|  |  | Simple mode | 13 | -0.2246 | 0.1608 | 0.1879 | -0.5398 | 0.0906 | 0.7989 | 0.5829 | 1.0949 |
|  |  | Weighted mode | 13 | -0.2420 | 0.1505 | 0.1338 | -0.5370 | 0.0530 | 0.7851 | 0.5845 | 1.0544 |
| UC | B. bifidum | MR Egger | 41 | -0.0282 | 0.0641 | 0.6621 | -0.1539 | 0.0974 | 0.9722 | 0.8573 | 1.1023 |
|  |  | Weighted median | 41 | -0.0298 | 0.0286 | 0.2961 | -0.0858 | 0.0261 | 0.9706 | 0.9177 | 1.0265 |
|  |  | Inverse variance weighted | 41 | -0.0342 | 0.0203 | 0.0924 | -0.0740 | 0.0056 | 0.9664 | 0.9287 | 1.0057 |
|  |  | Simple mode | 41 | -0.0048 | 0.0578 | 0.9345 | -0.1181 | 0.1085 | 0.9952 | 0.8886 | 1.1146 |
|  |  | Weighted mode | 41 | -0.0155 | 0.0520 | 0.7672 | -0.1174 | 0.0864 | 0.9846 | 0.8892 | 1.0903 |
| B. bifidum | CD11b on Mo MDSC | MR Egger | 13 | 0.1120 | 0.3014 | 0.7172 | -0.4787 | 0.7028 | 1.1185 | 0.6196 | 2.0194 |
|  |  | Weighted median | 13 | 0.3162 | 0.1776 | 0.0751 | -0.0320 | 0.6644 | 1.3719 | 0.9685 | 1.9433 |
|  |  | Inverse variance weighted | 13 | 0.3743 | 0.1195 | 0.0017 | 0.1401 | 0.6086 | 1.4540 | 1.1504 | 1.8378 |
|  |  | Simple mode | 13 | 0.4470 | 0.2620 | 0.1138 | -0.0666 | 0.9606 | 1.5636 | 0.9355 | 2.6133 |
|  |  | Weighted mode | 13 | 0.3022 | 0.2135 | 0.1823 | -0.1162 | 0.7205 | 1.3528 | 0.8903 | 2.0555 |
| CD11b on Mo MDSC | UC | MR Egger | 15 | -0.1410 | 0.0736 | 0.0778 | -0.2853 | 0.0033 | 0.8685 | 0.7518 | 1.0033 |
|  |  | Weighted median | 15 | -0.0458 | 0.0472 | 0.3316 | -0.1383 | 0.0467 | 0.9552 | 0.8709 | 1.0478 |
|  |  | Inverse variance weighted | 15 | -0.1028 | 0.0413 | 0.0128 | -0.1838 | -0.0219 | 0.9023 | 0.8321 | 0.9784 |
|  |  | Simple mode | 15 | -0.0821 | 0.0891 | 0.3720 | -0.2567 | 0.0924 | 0.9212 | 0.7736 | 1.0968 |
|  |  | Weighted mode | 15 | -0.0438 | 0.0571 | 0.4564 | -0.1557 | 0.0682 | 0.9572 | 0.8558 | 1.0706 |

## Supplementary Figures


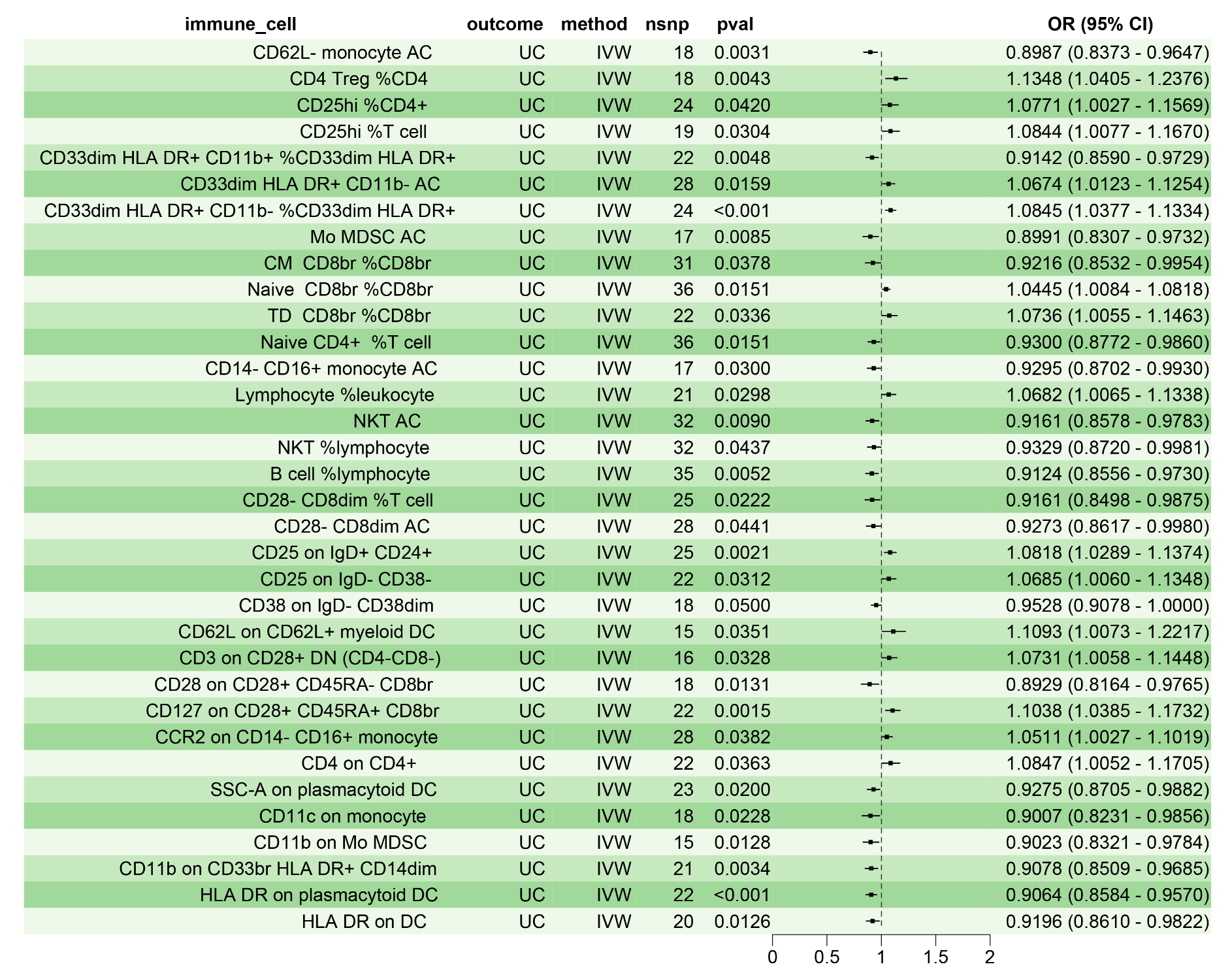


**Supplementary Figure 1.** Effect of immune cells on UC.


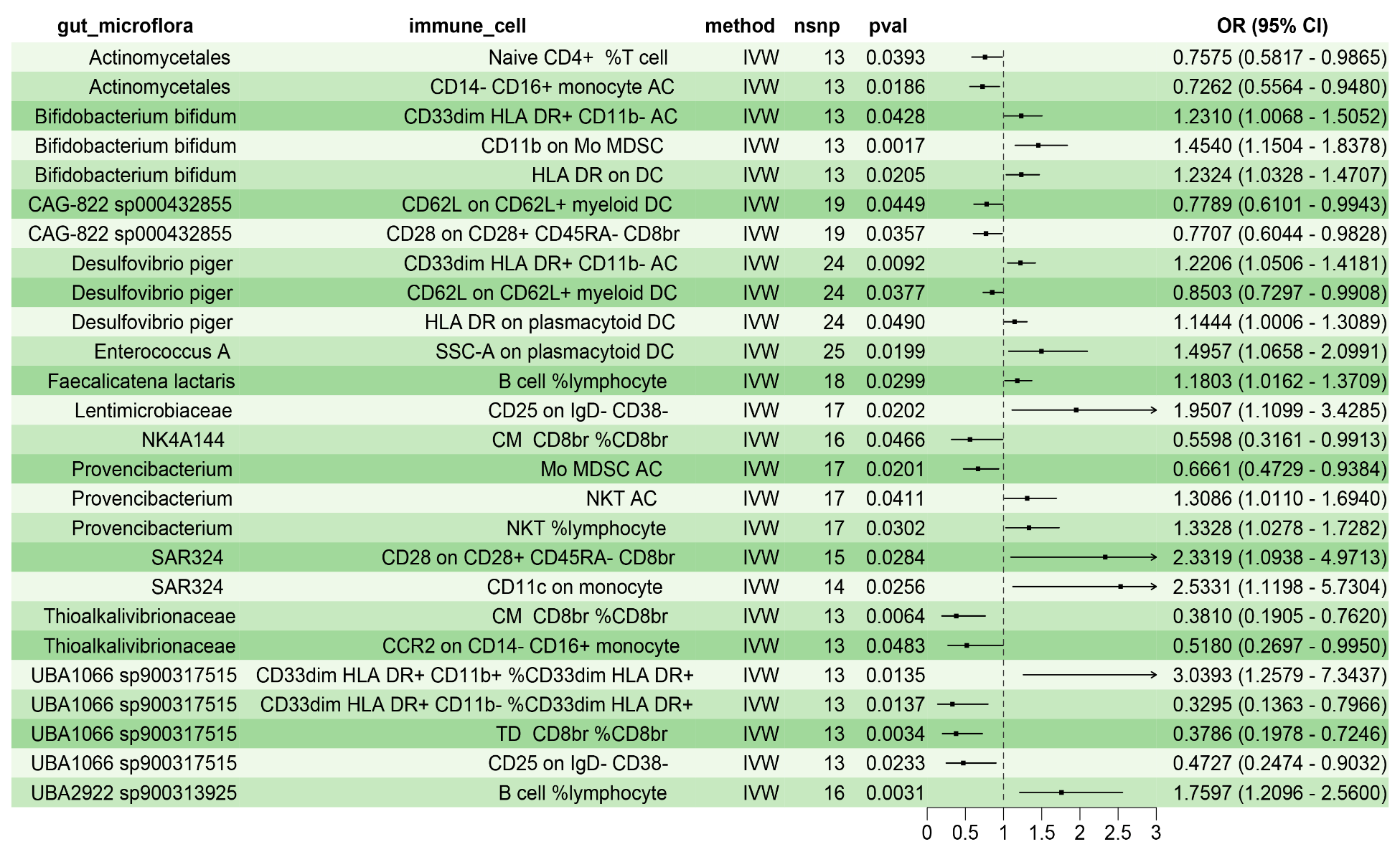


**Supplementary Figure 2.** Effect of the gut microbiota on immune cells.

## Supplementary Figure File1. Forest, funnel, scatter plots and leave-one-out sensitivity analysis of causal relationships between exposure and outcome. (A) forest plot; (B) funnel plot; (C) scatter plot; (D) leave-one-out sensitivity analysis.

### Actinomycetales on UC.


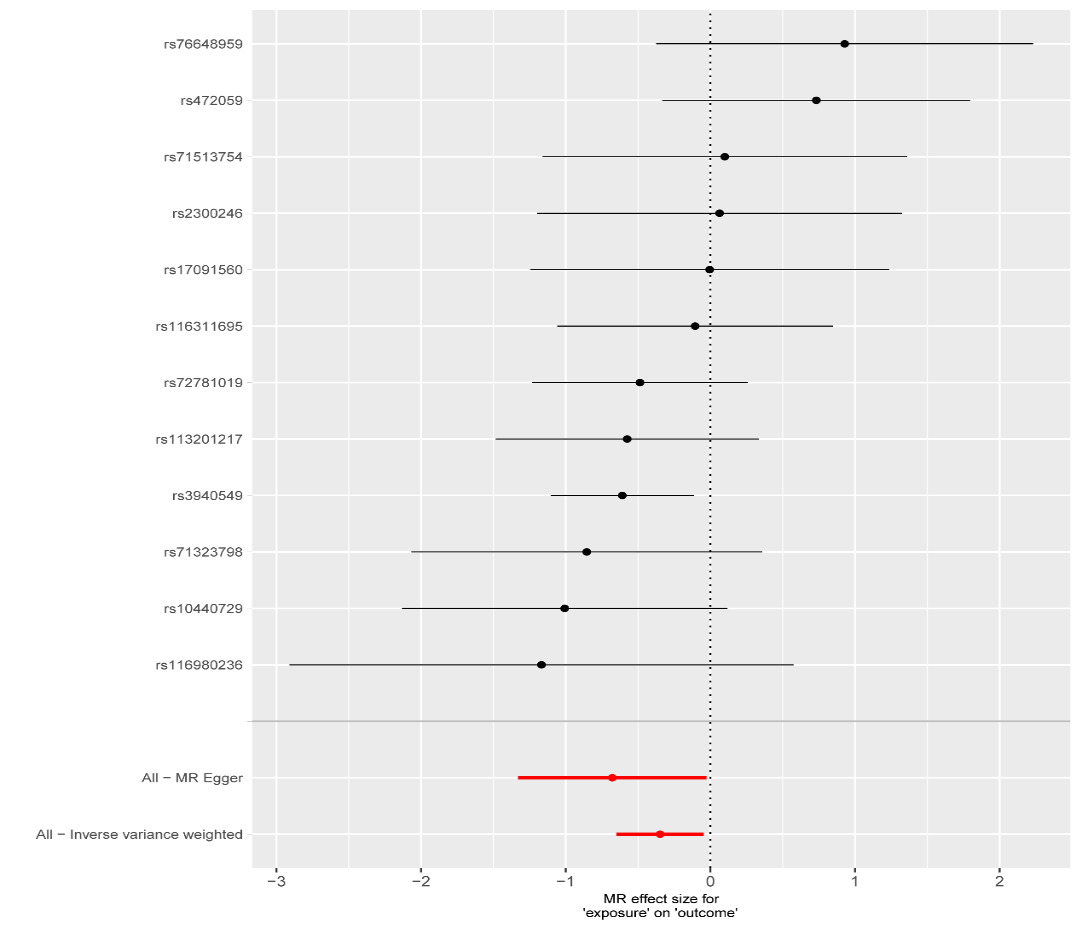

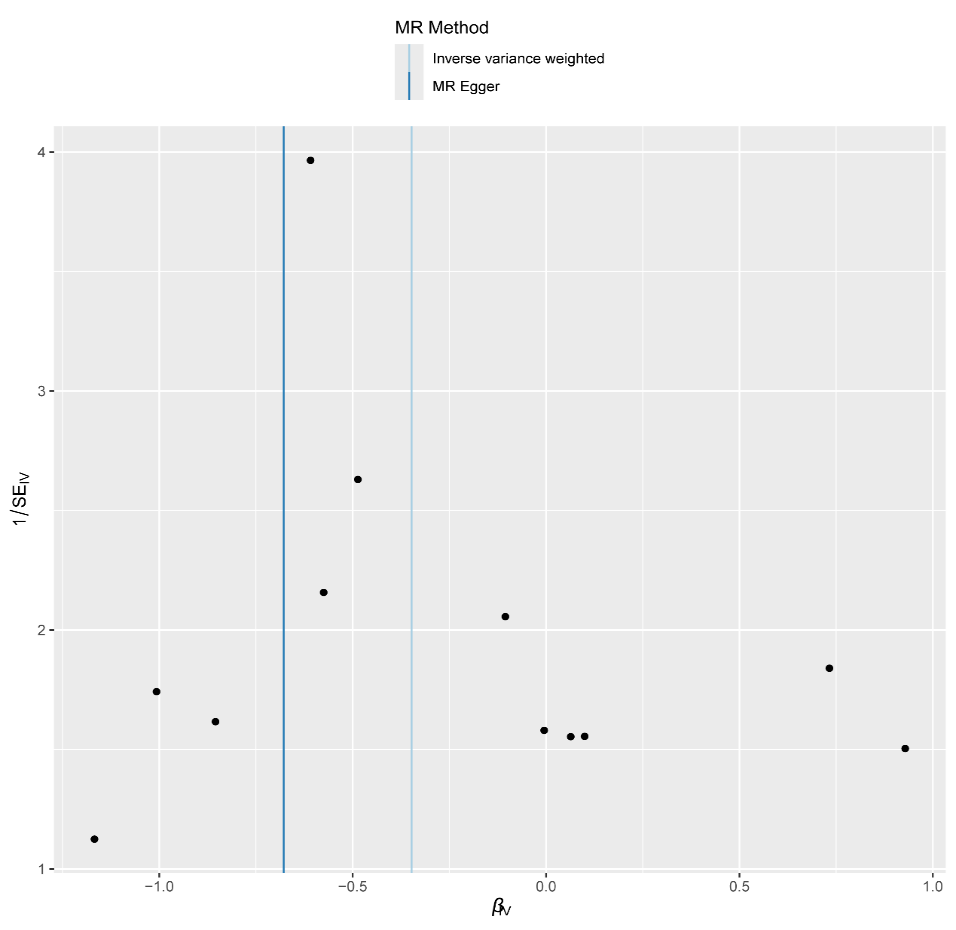


**（A） (B)**


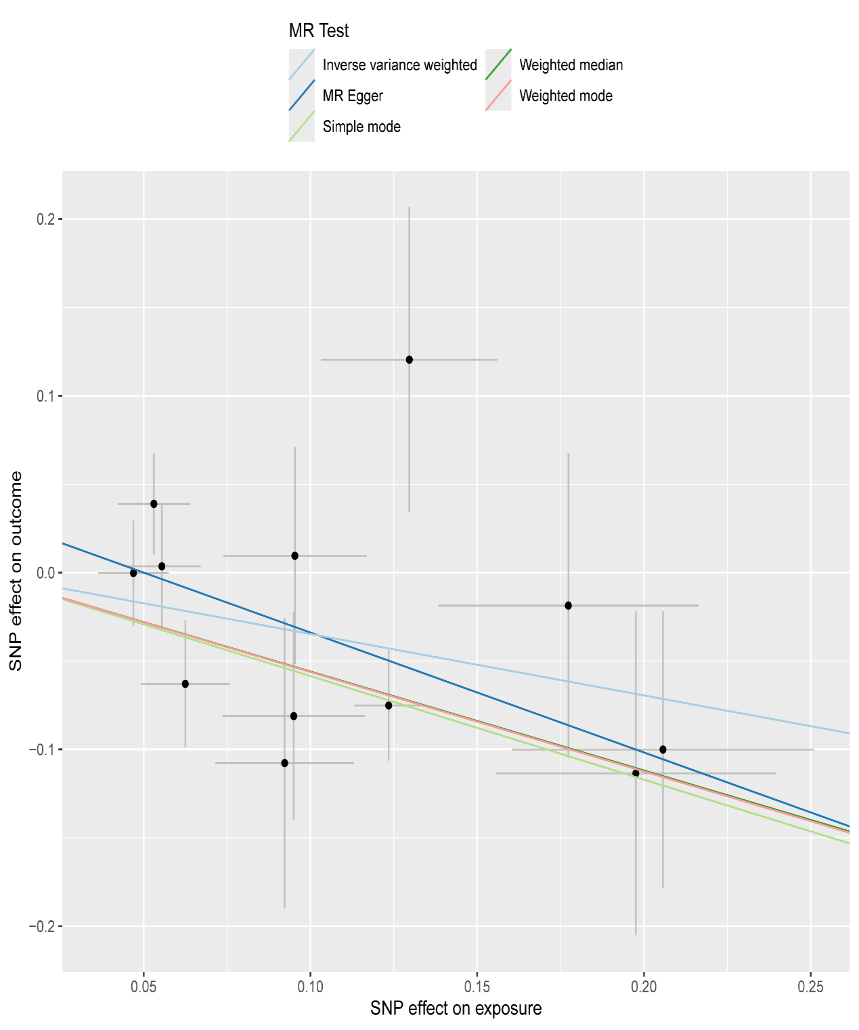

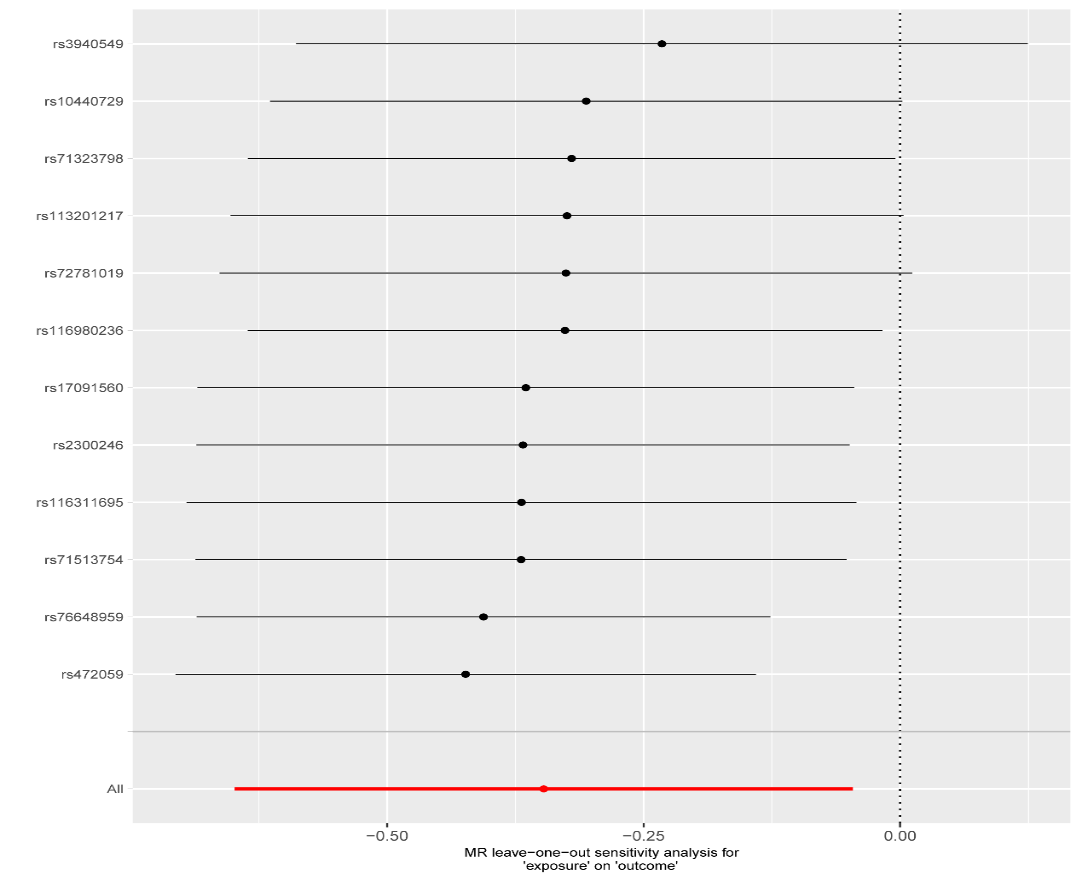


**(C) (D)**

### Azorhizobium on UC.


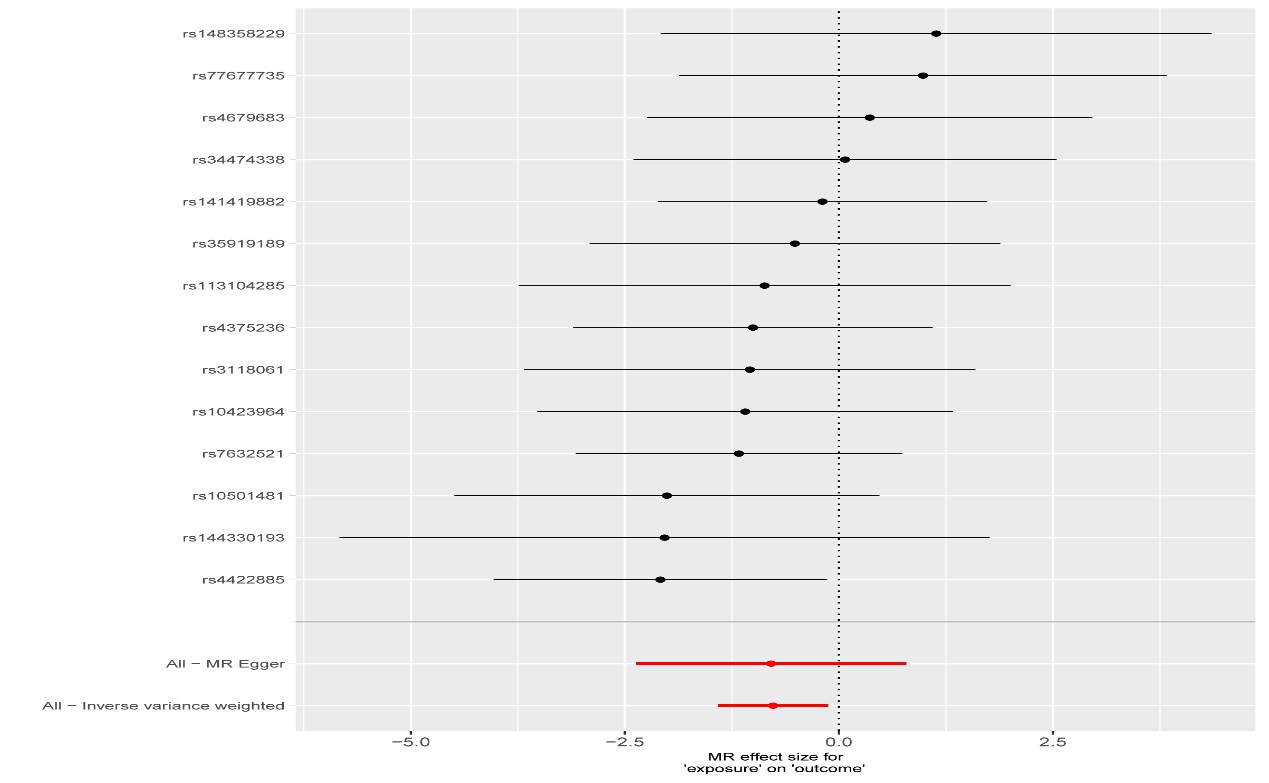

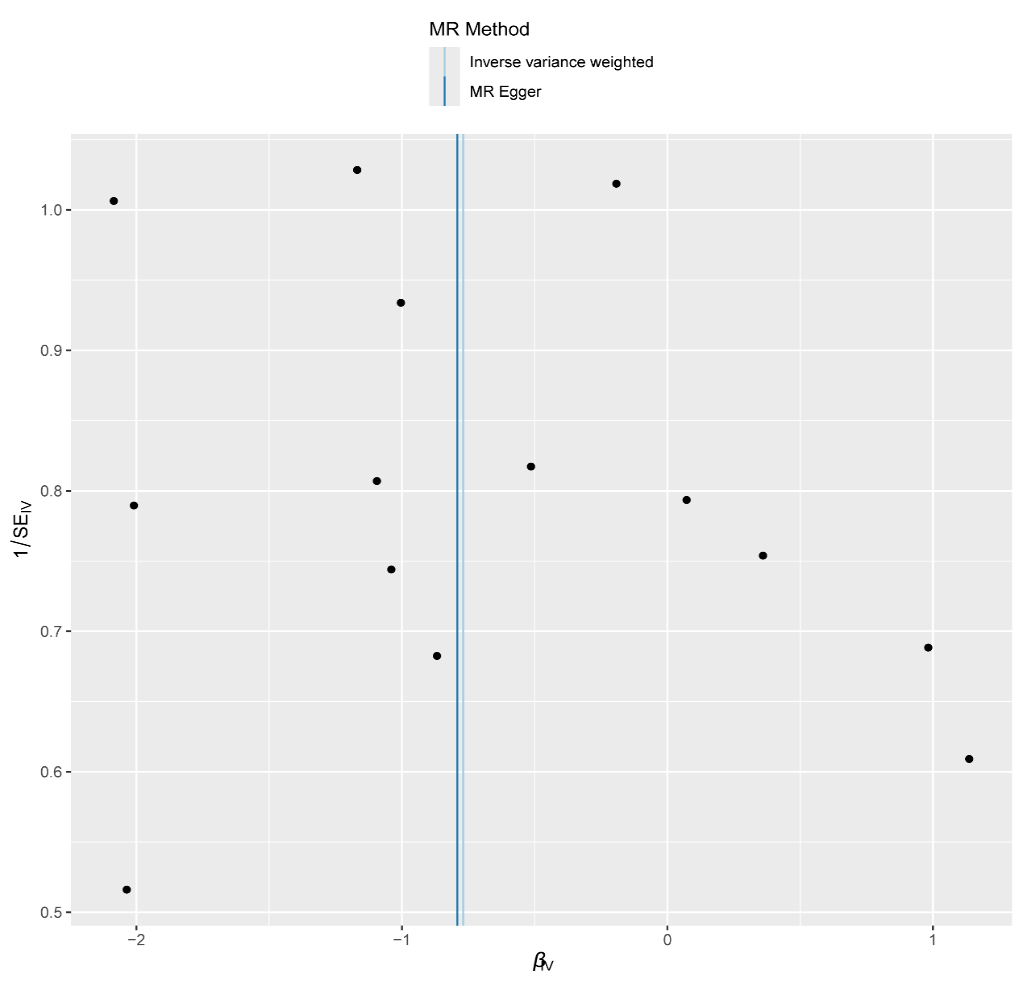


**(A) (B)**


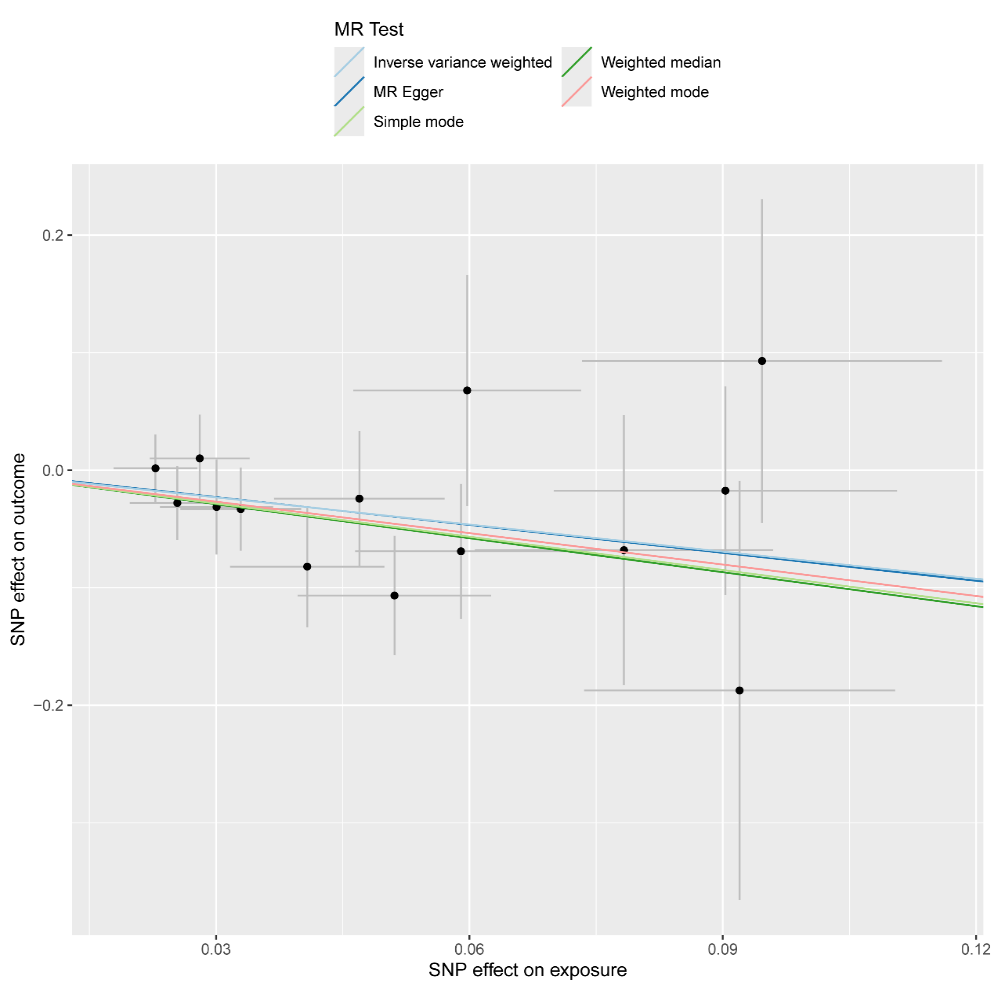

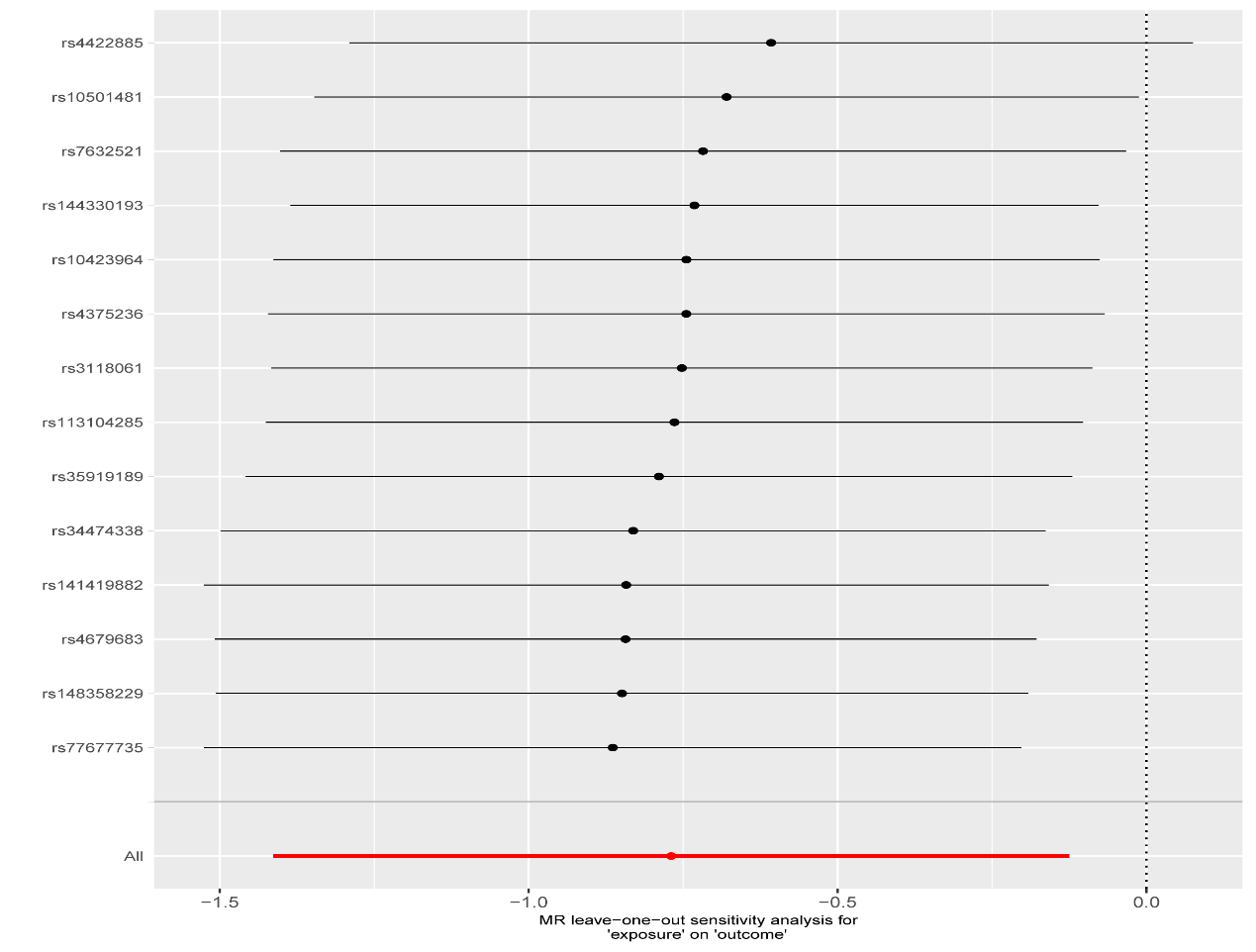


**(C) (D)**

### Bifidobacterium adolescentis on UC.


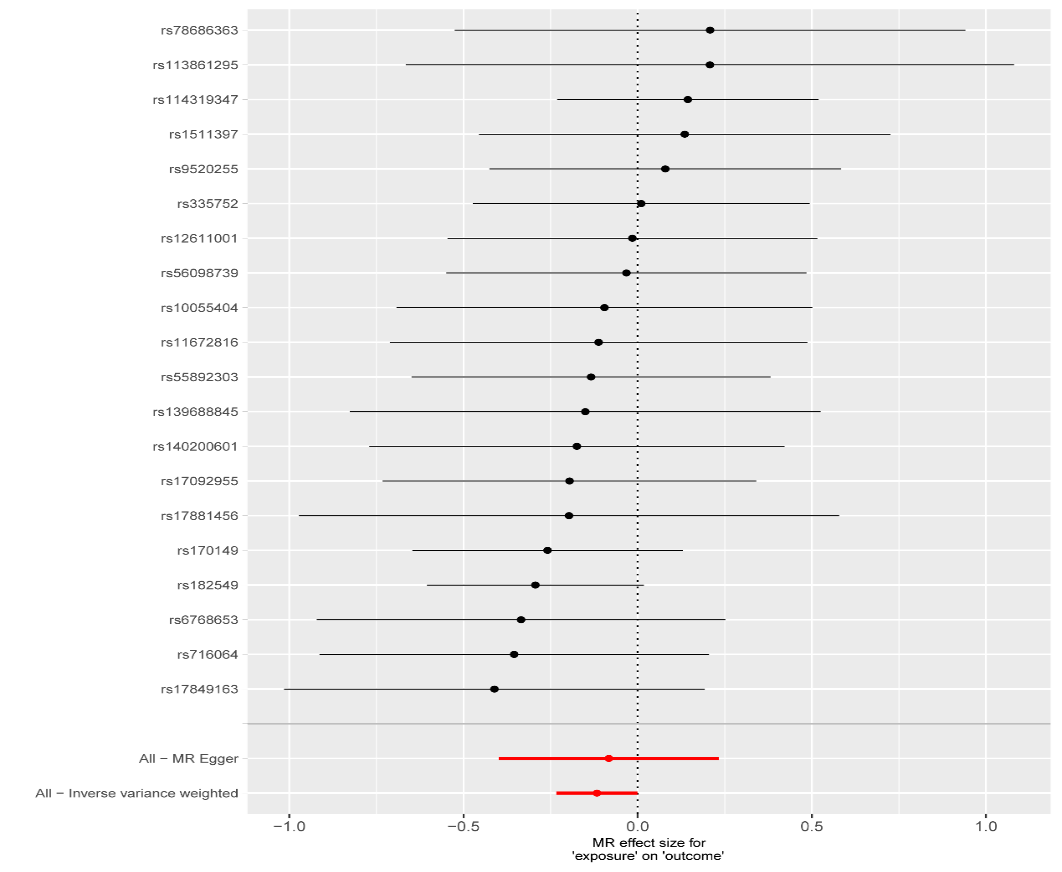

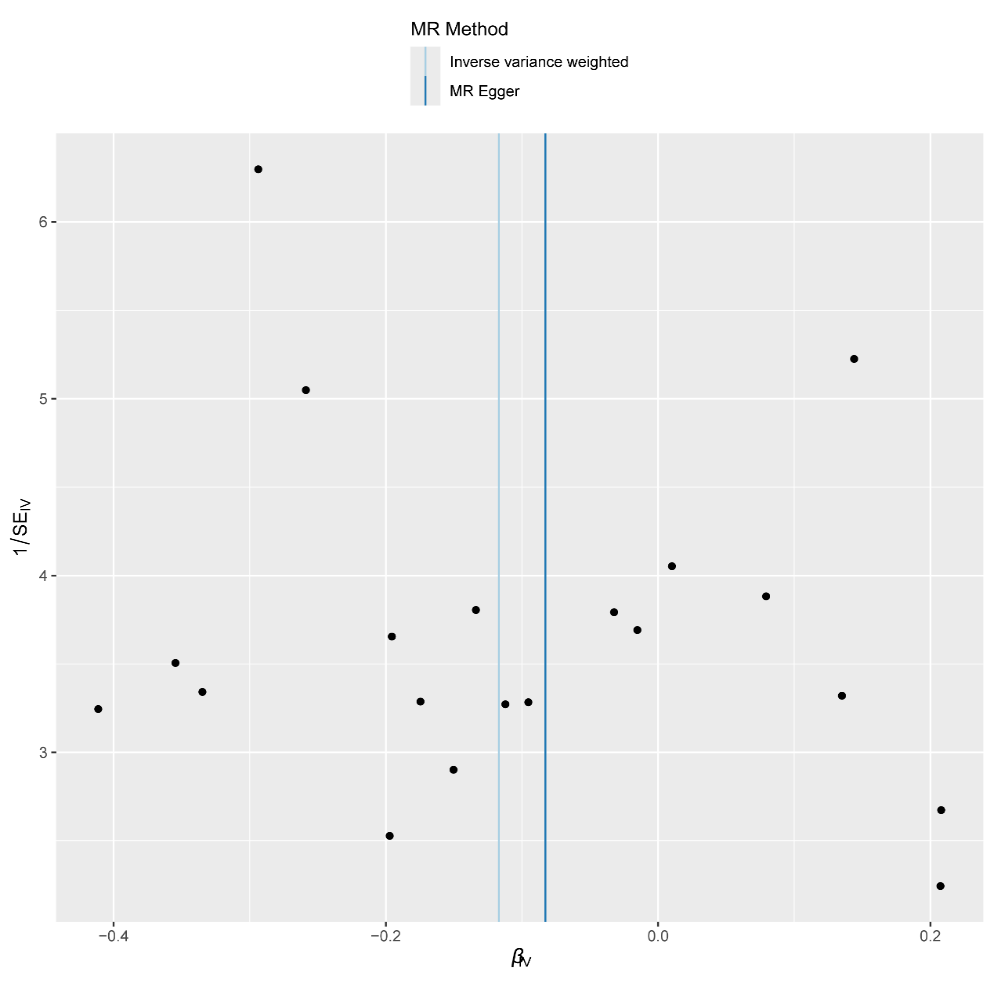


**(A) (B)**


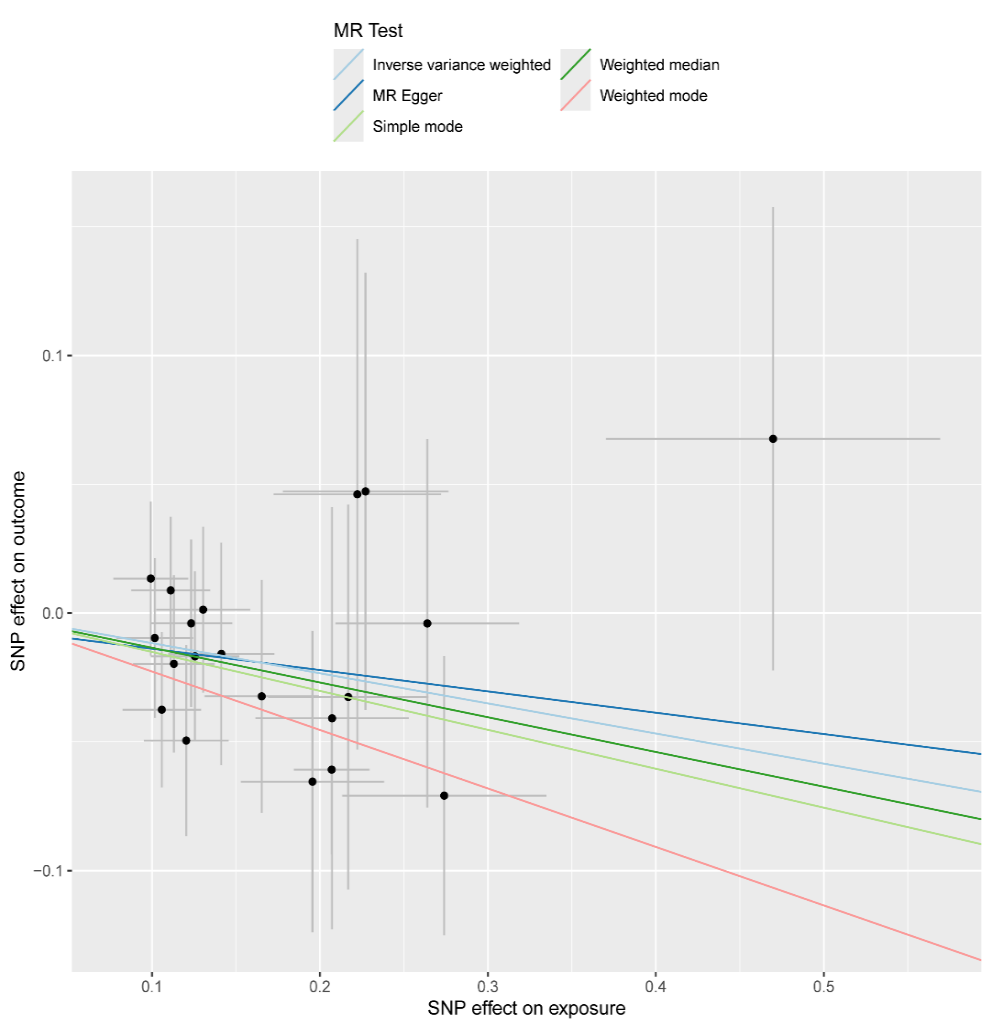

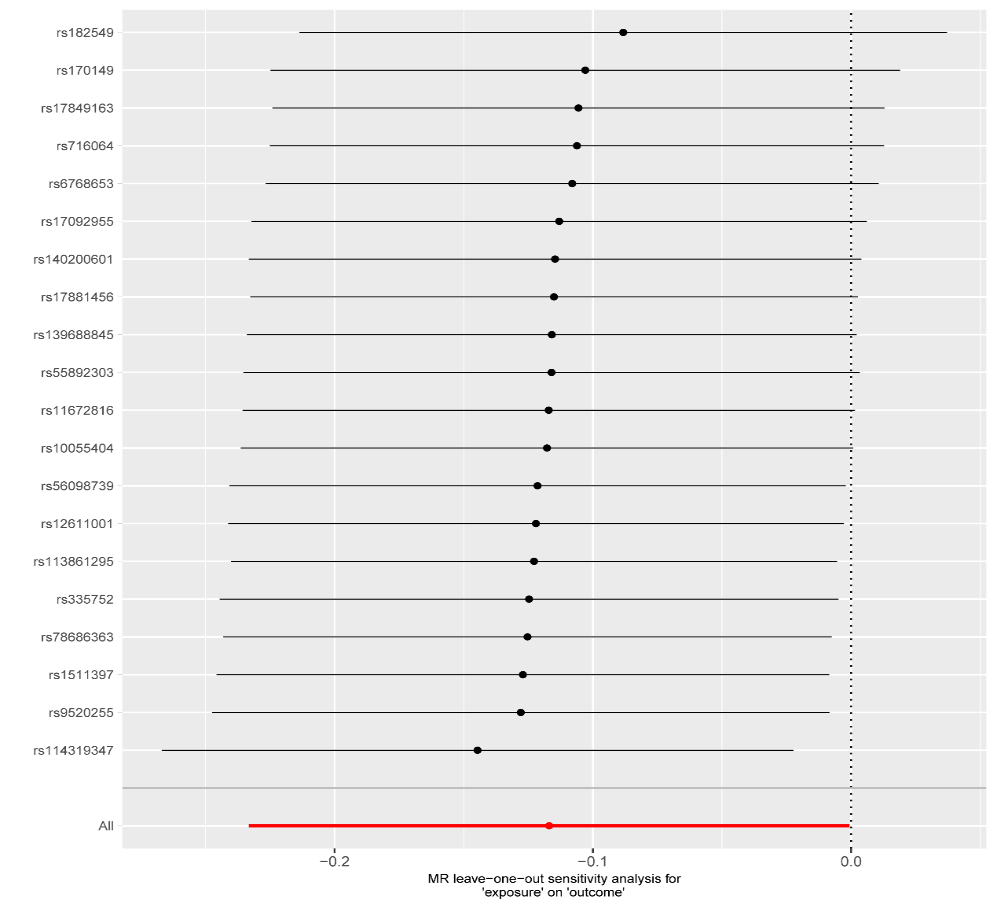


**(C) (D)**

### Bifidobacterium bifidum on UC.


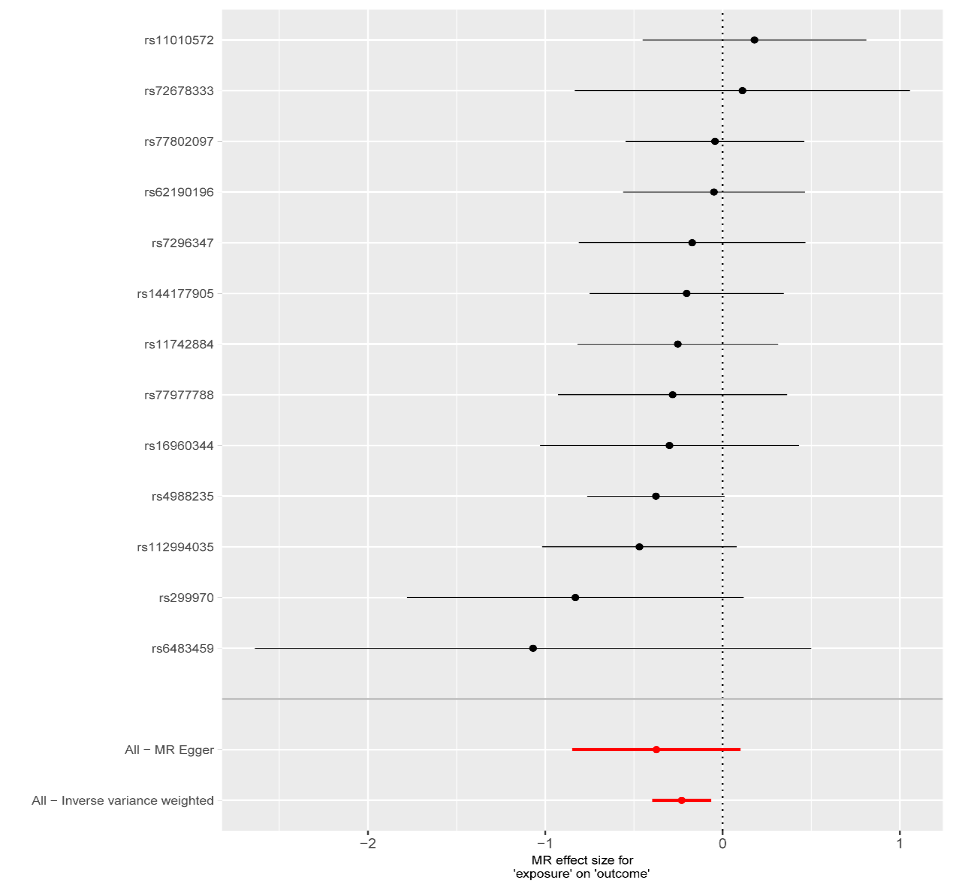

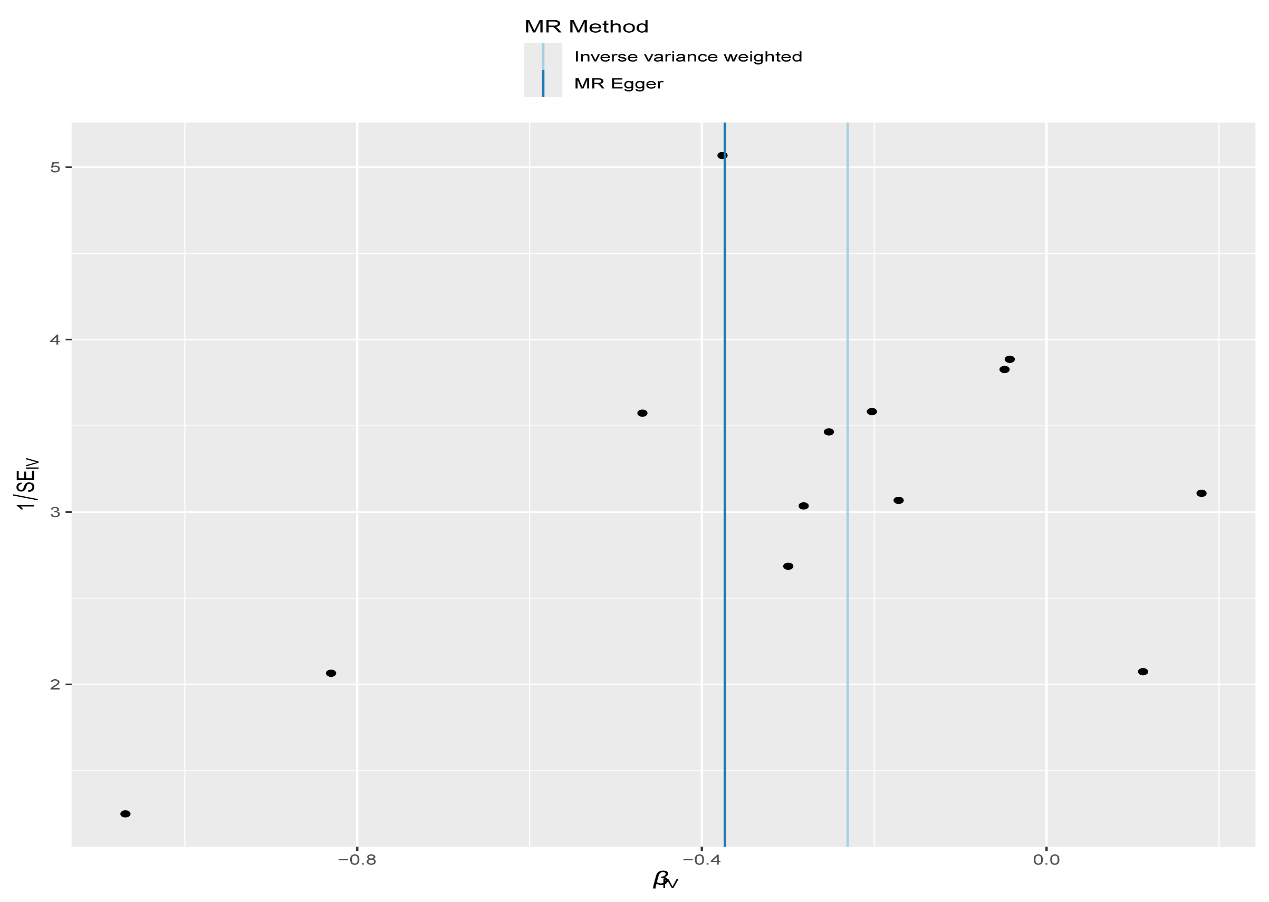


**(A) (B)**


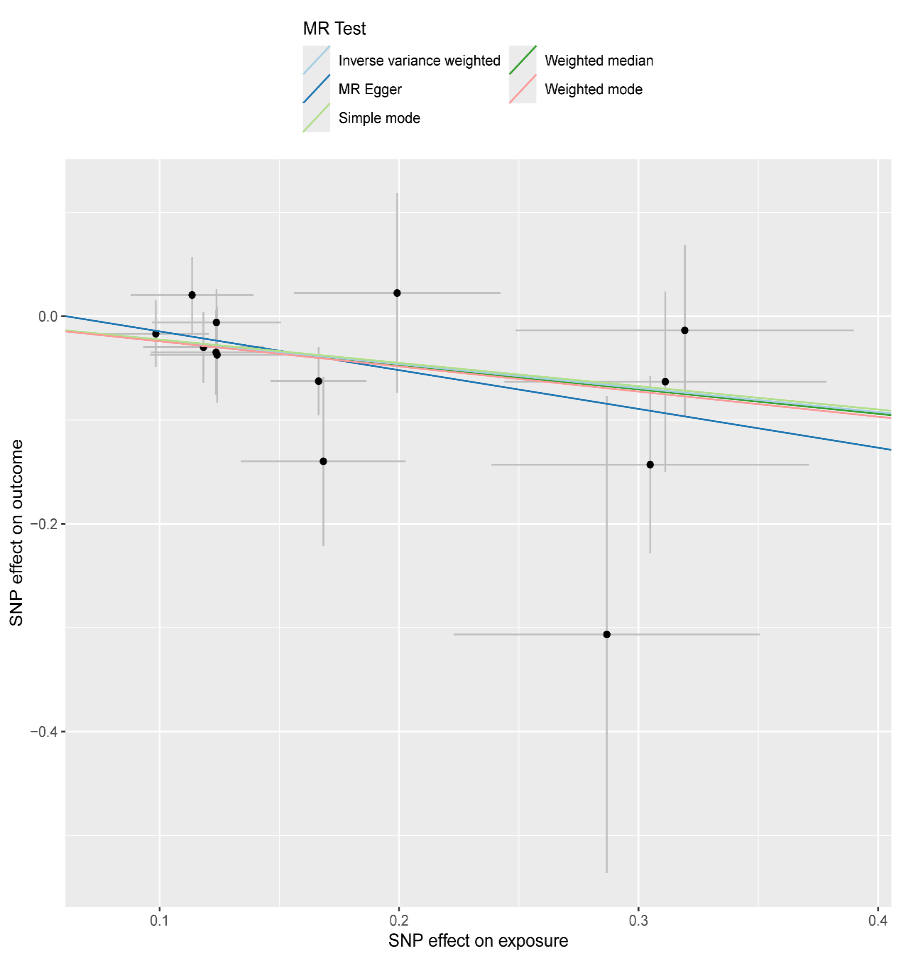

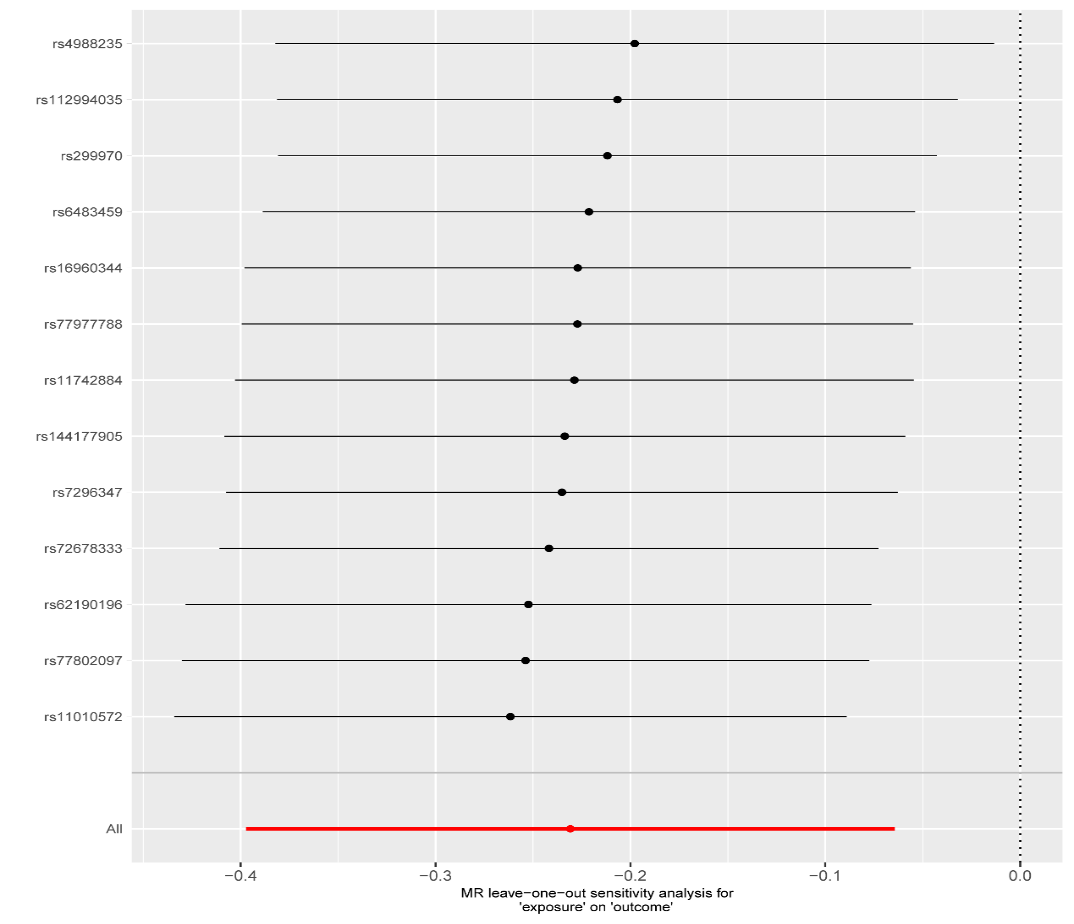


**(C) (D)**

### Bifidobacterium pseudocatenulatum on UC.


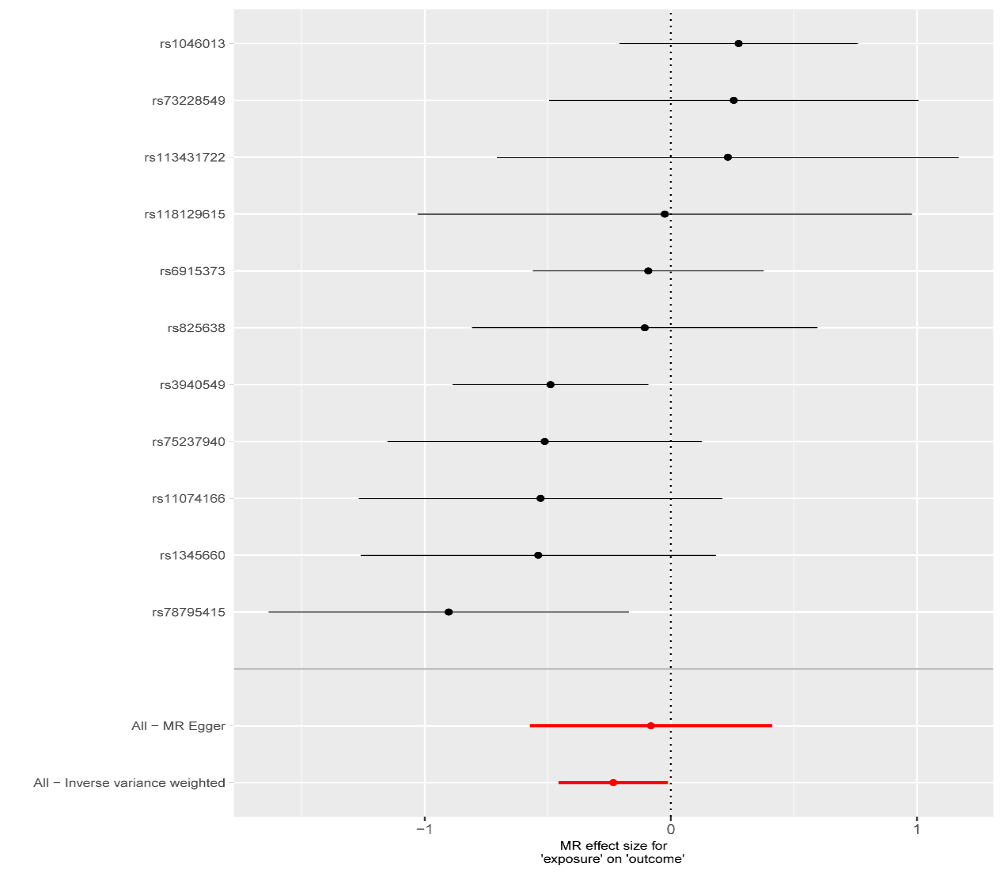

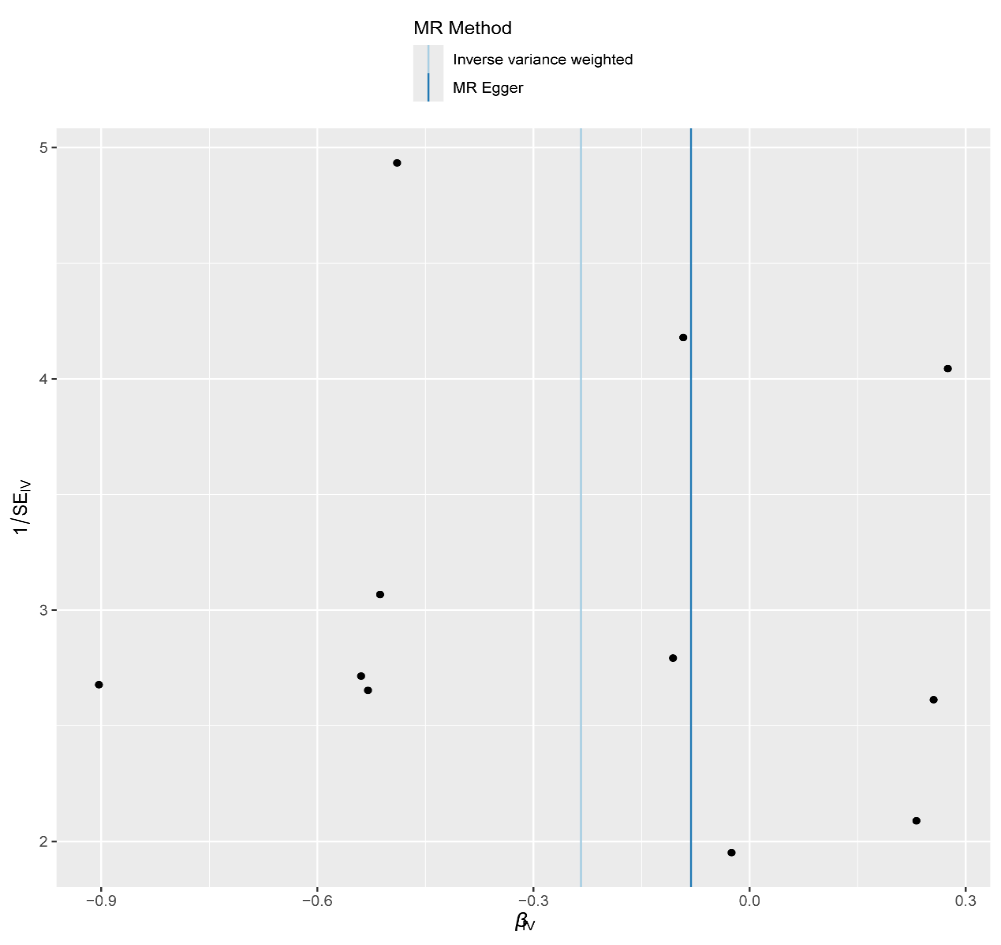


**(A) (B)**


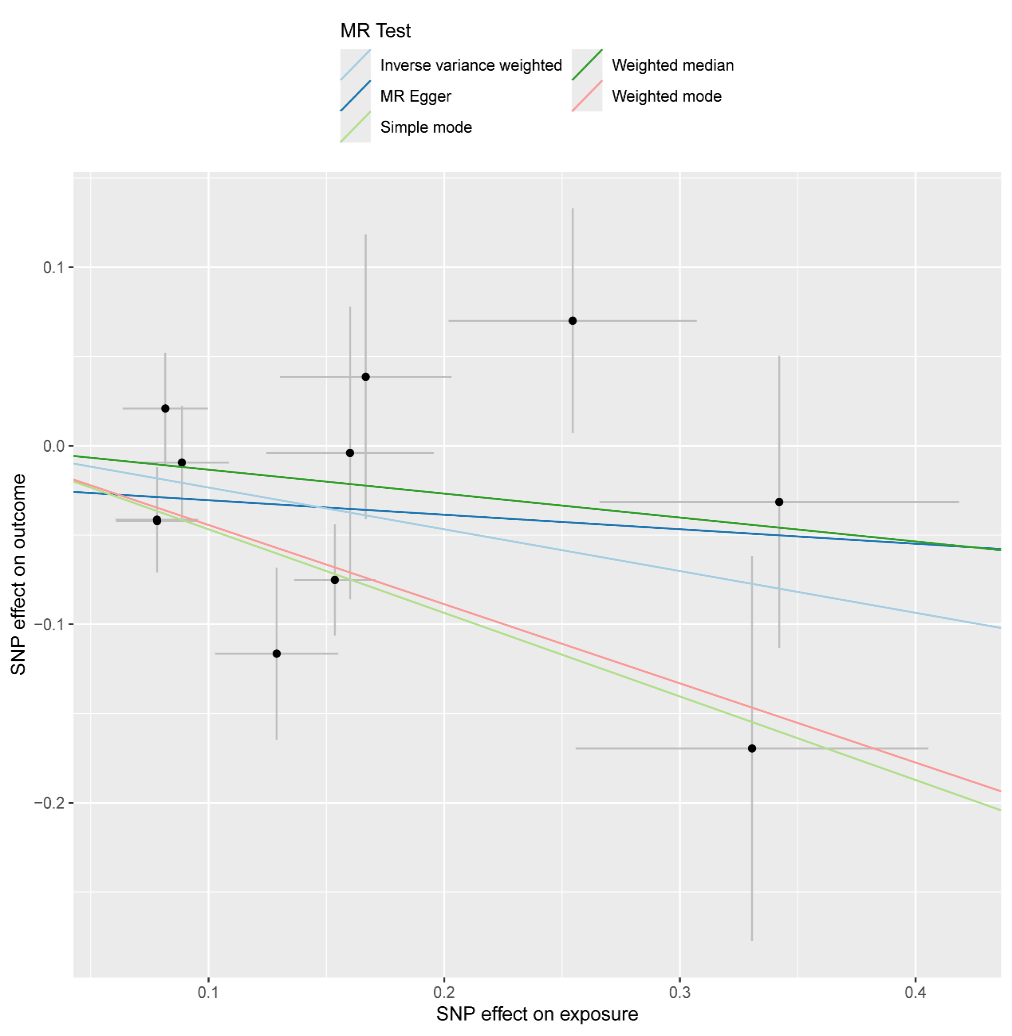

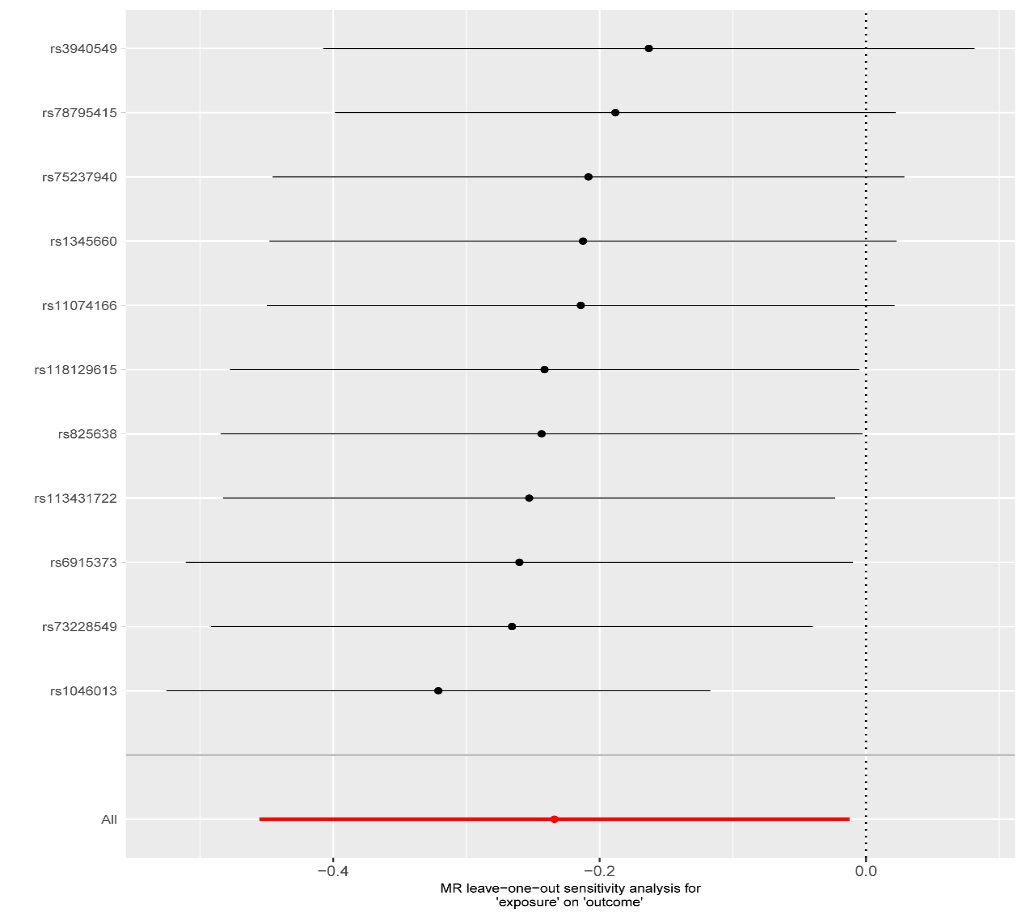


**(C) (D)**

### CAG-822 sp000432855 on UC.


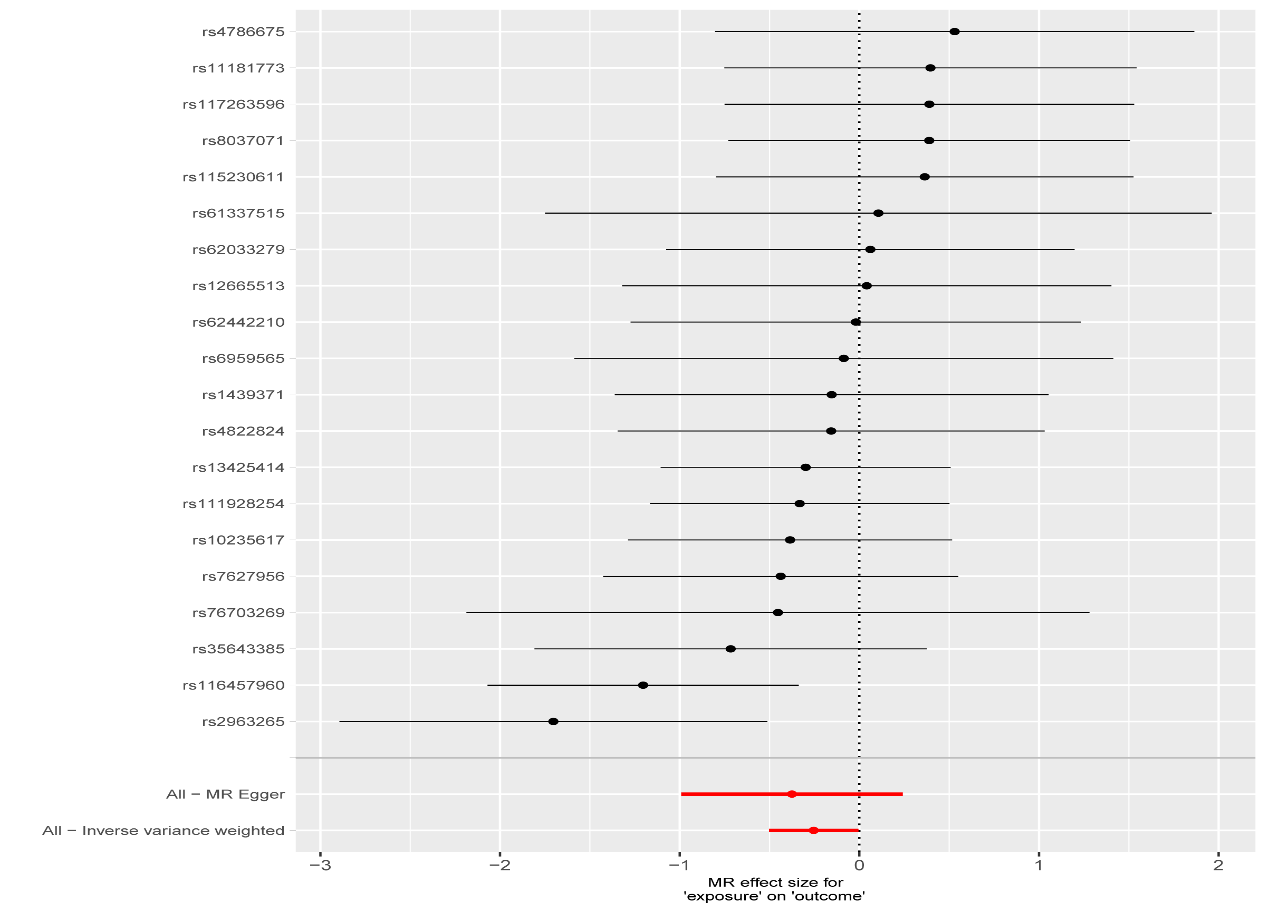

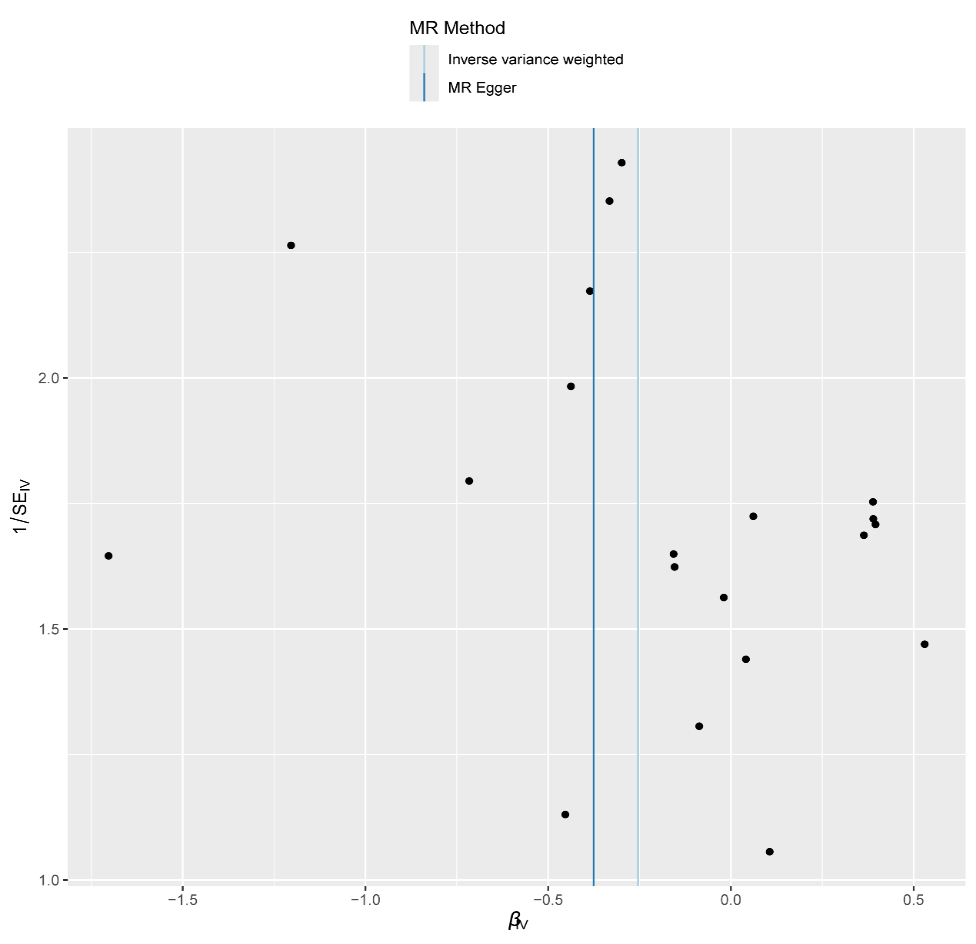


**(A) (B)**


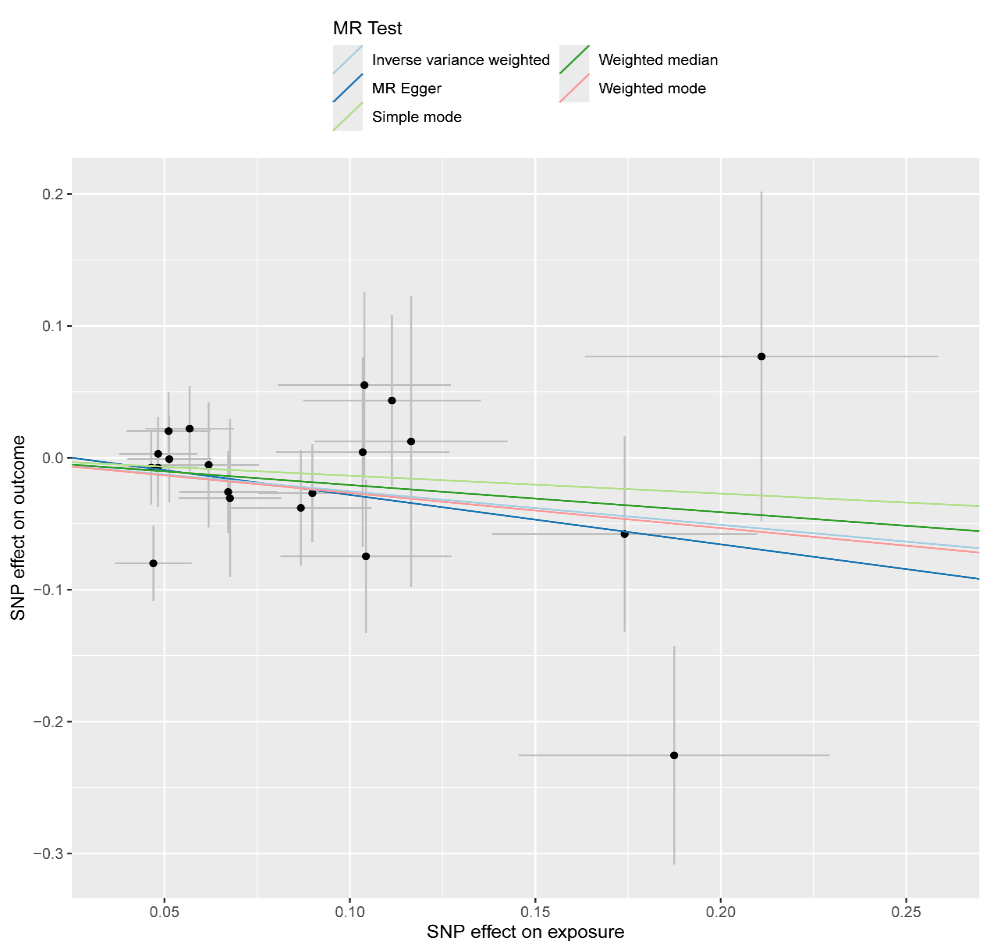

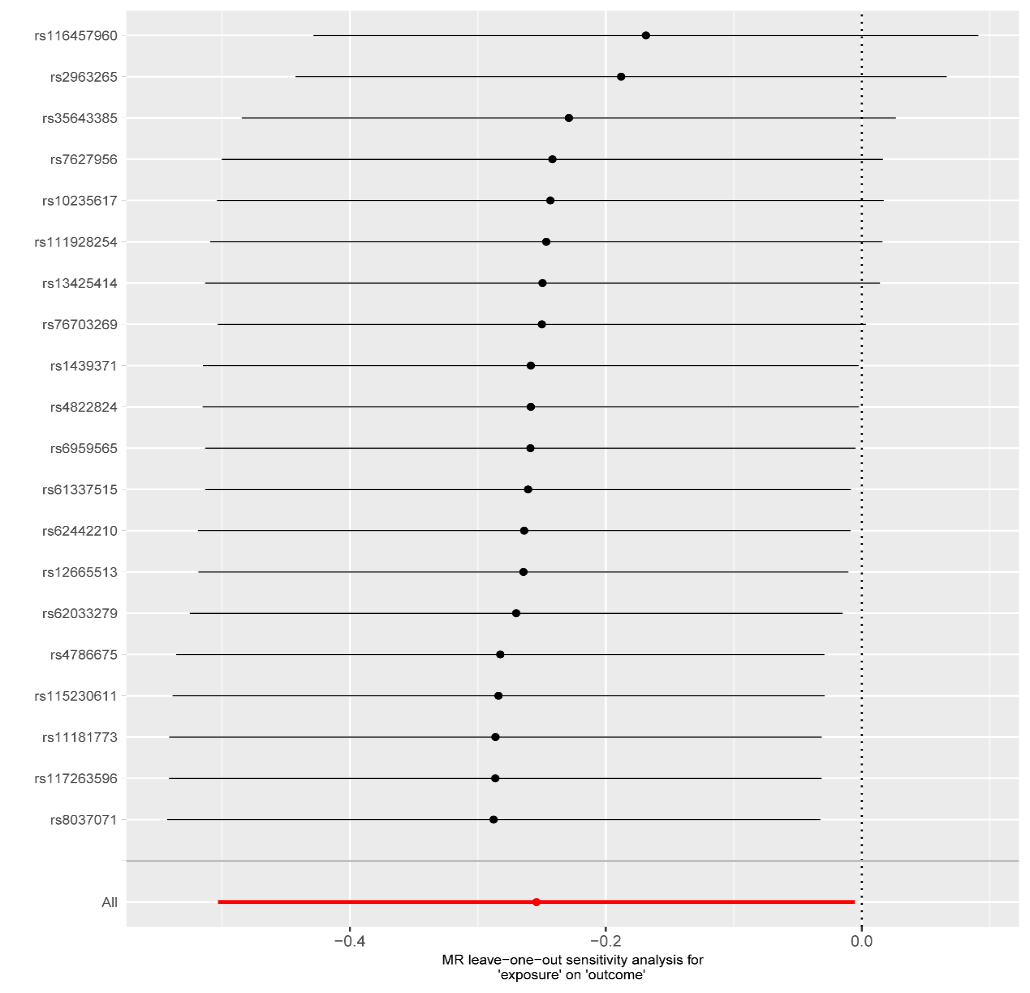


**(C) (D)**

### Desulfovibrio piger on UC.


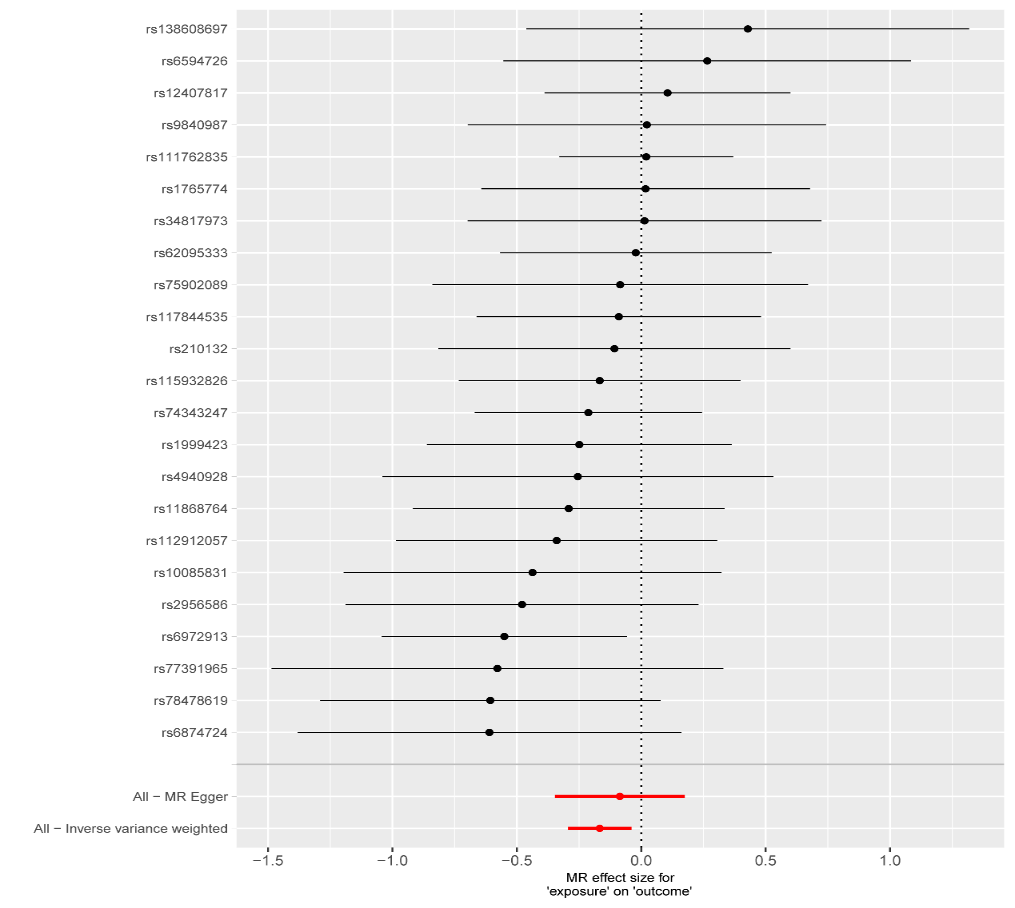

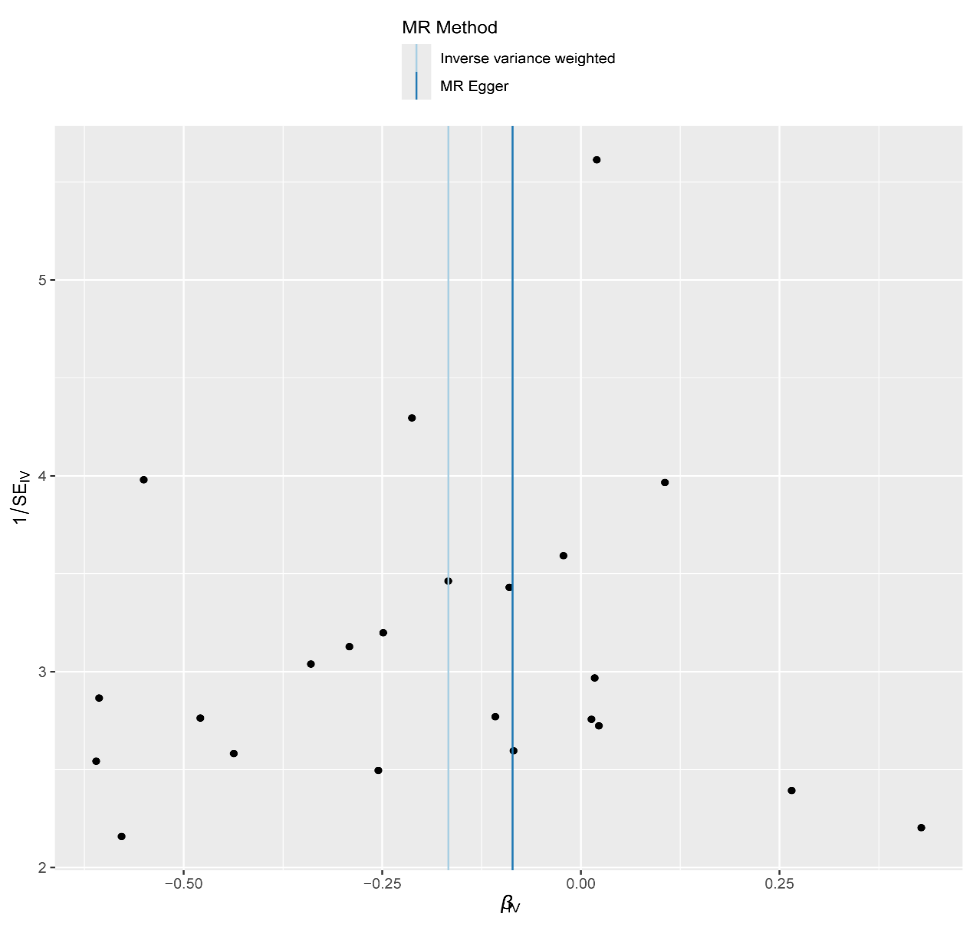


**(A) (B)_**


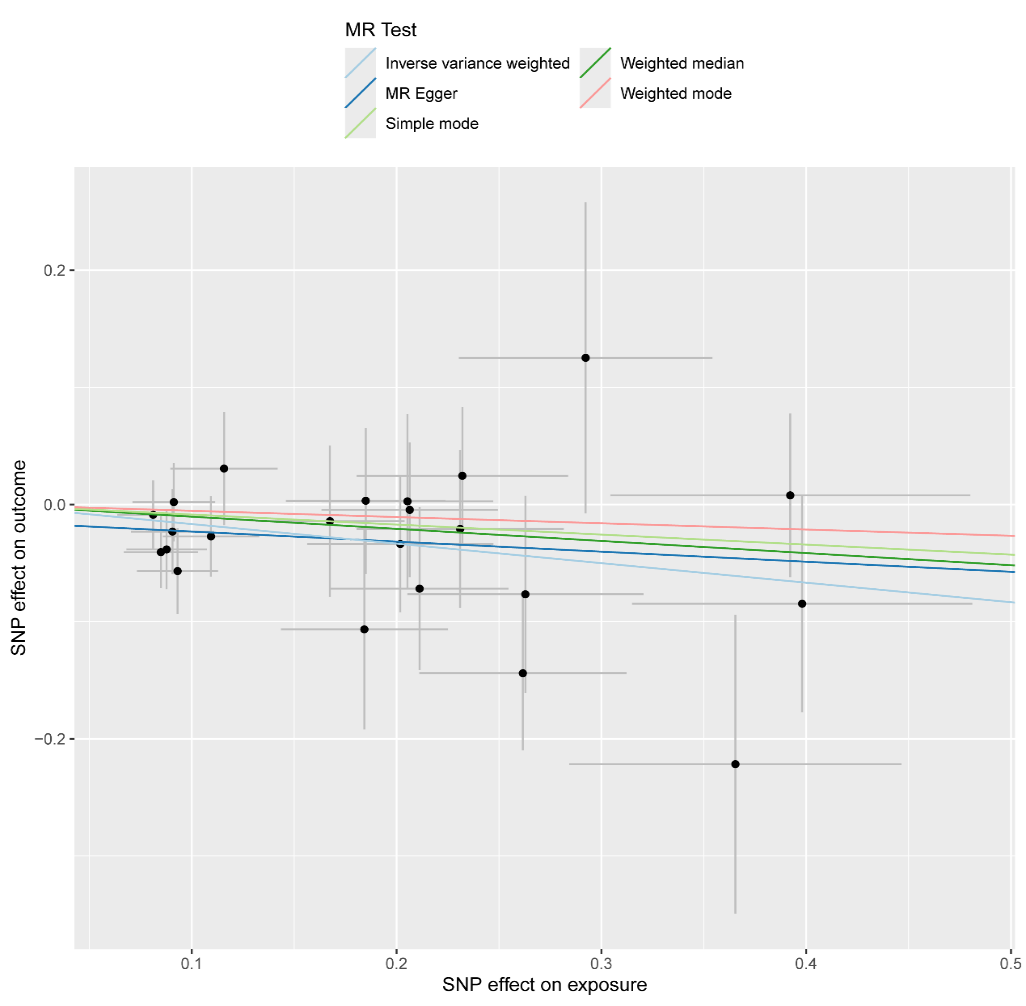

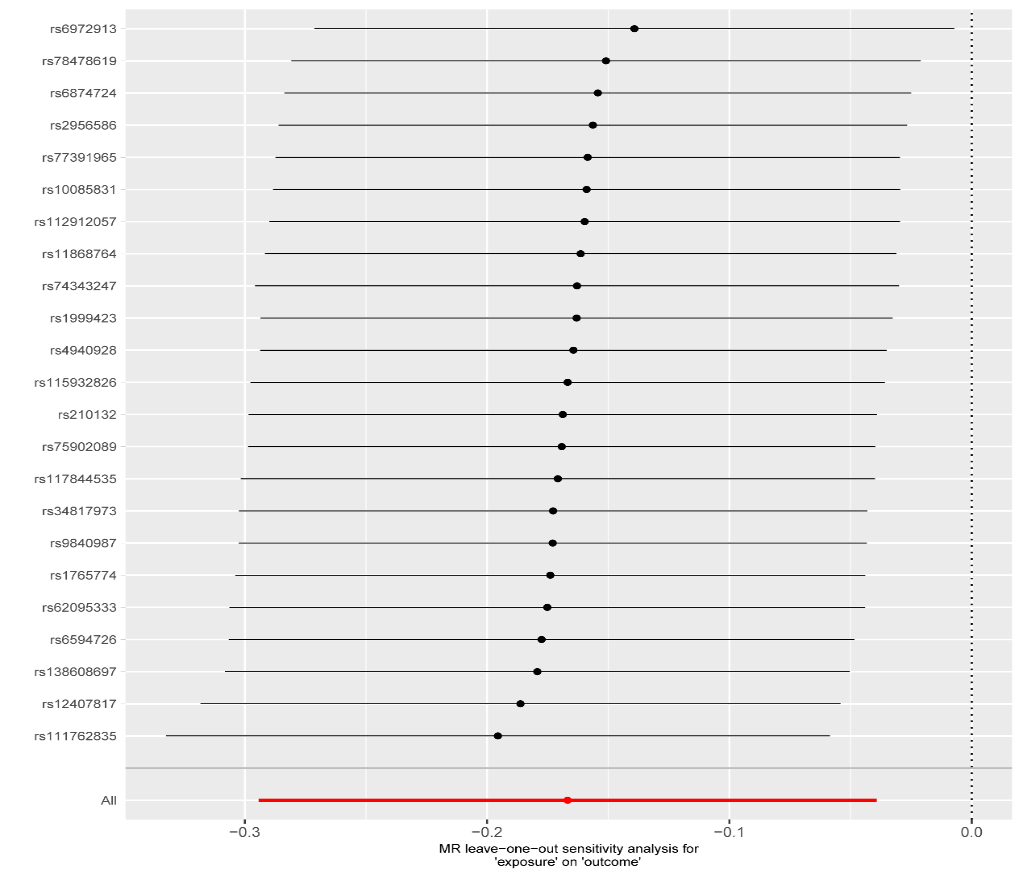


**(C) (D)**

### Enterococcus A on UC.


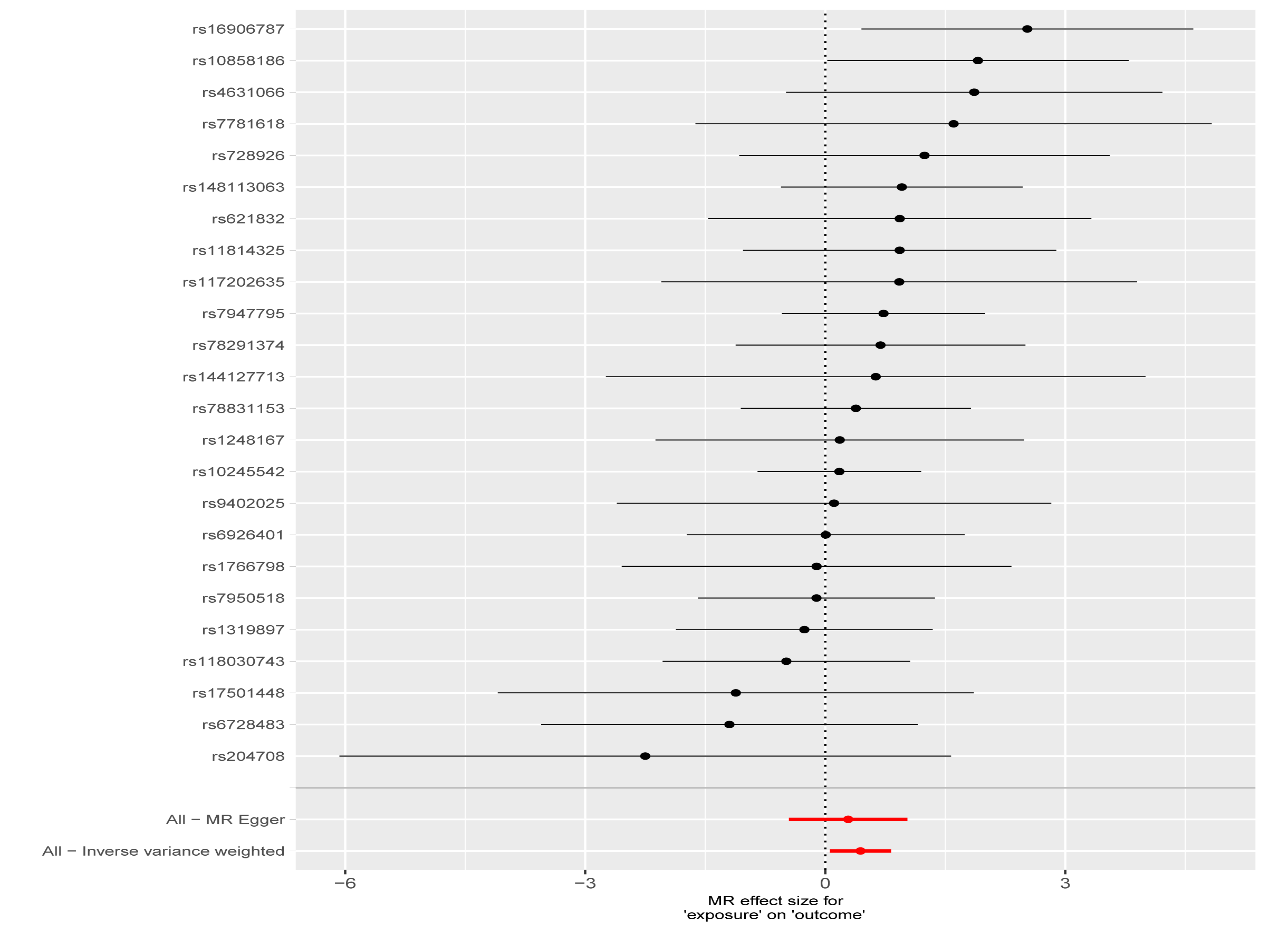

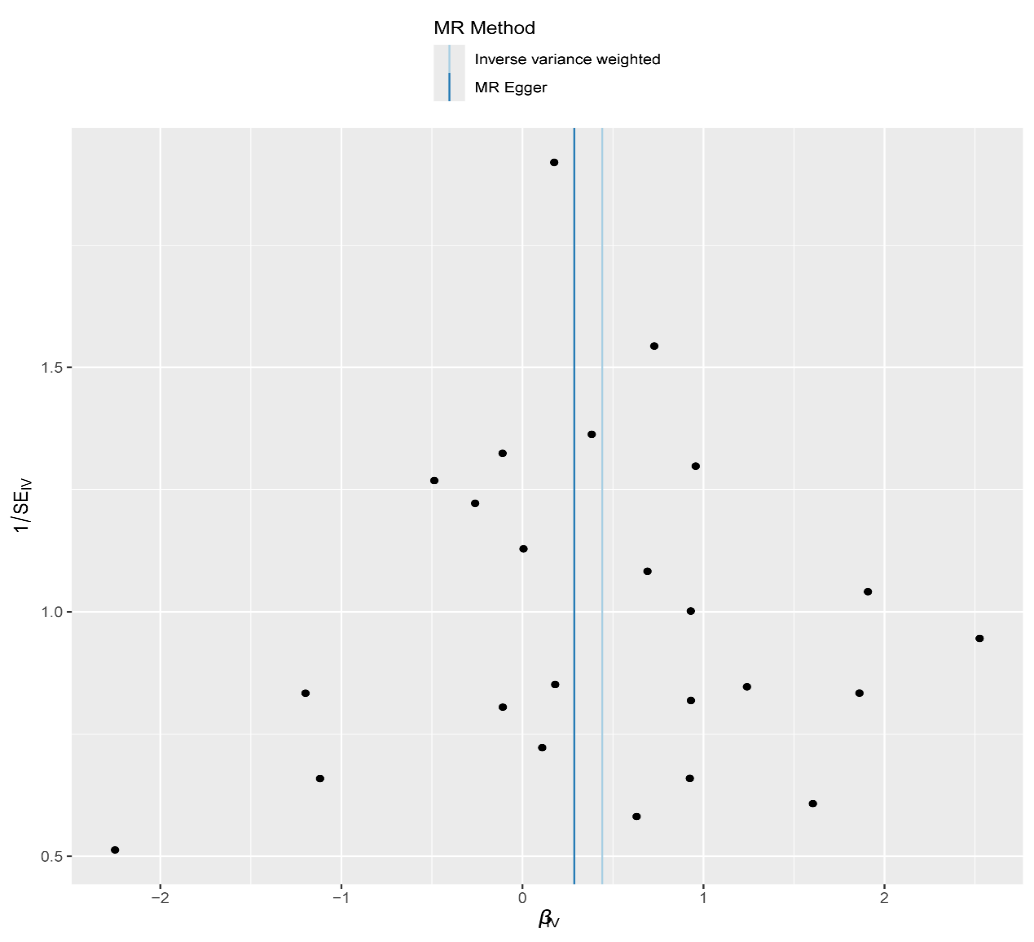


**(A) (B)**


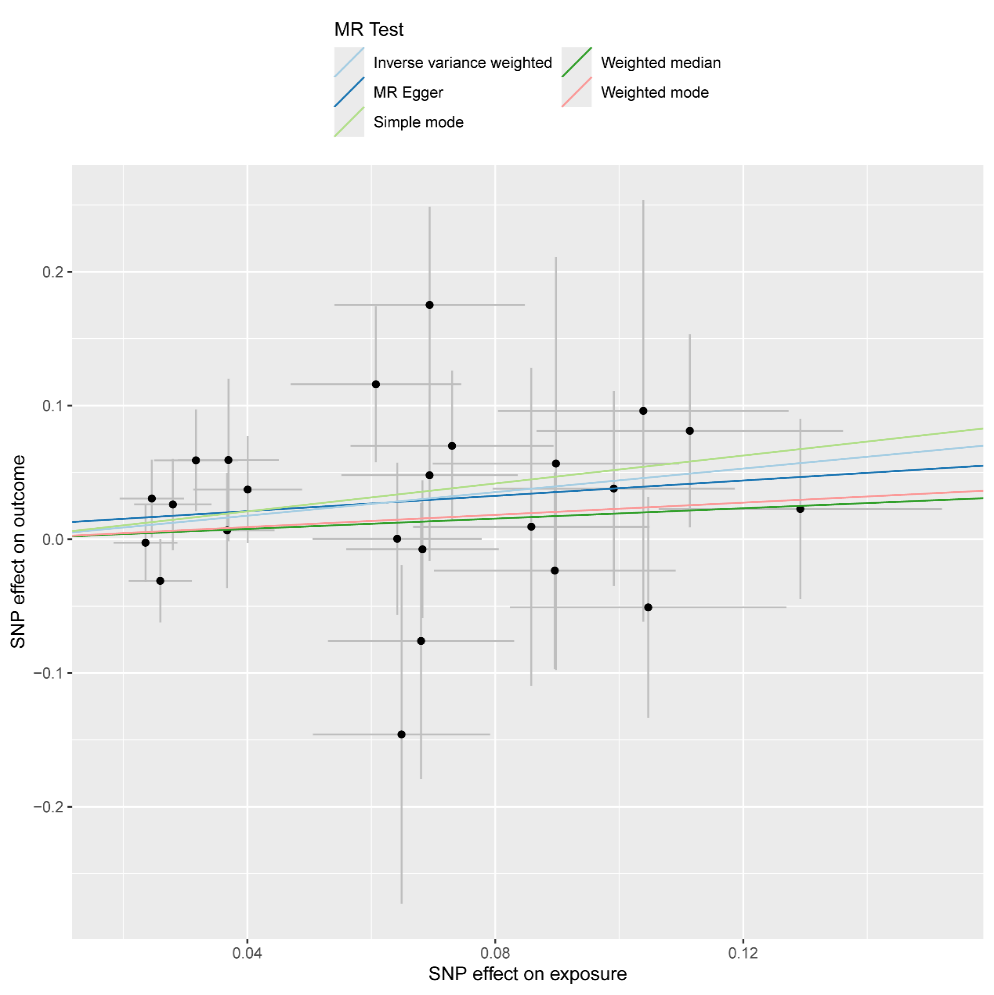

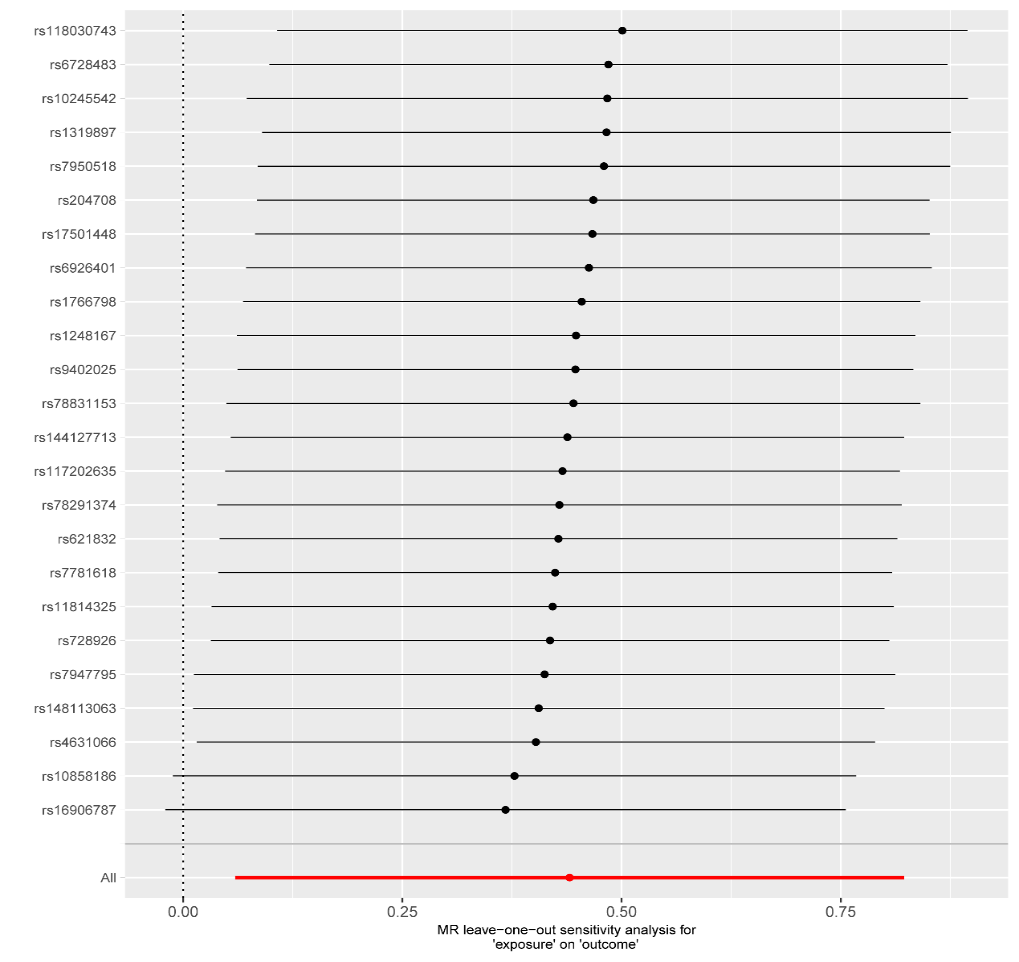


**(C) (D)**

### Eubacterium R coprostanoligenes on UC.


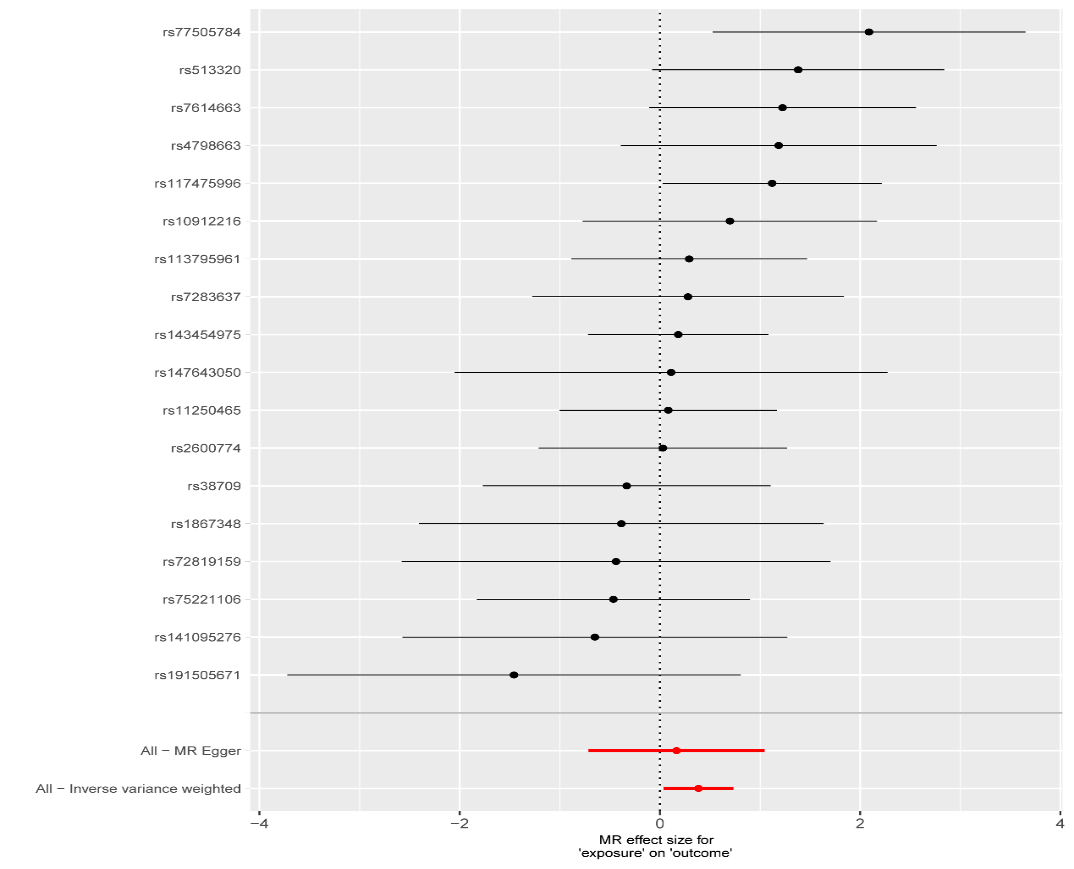

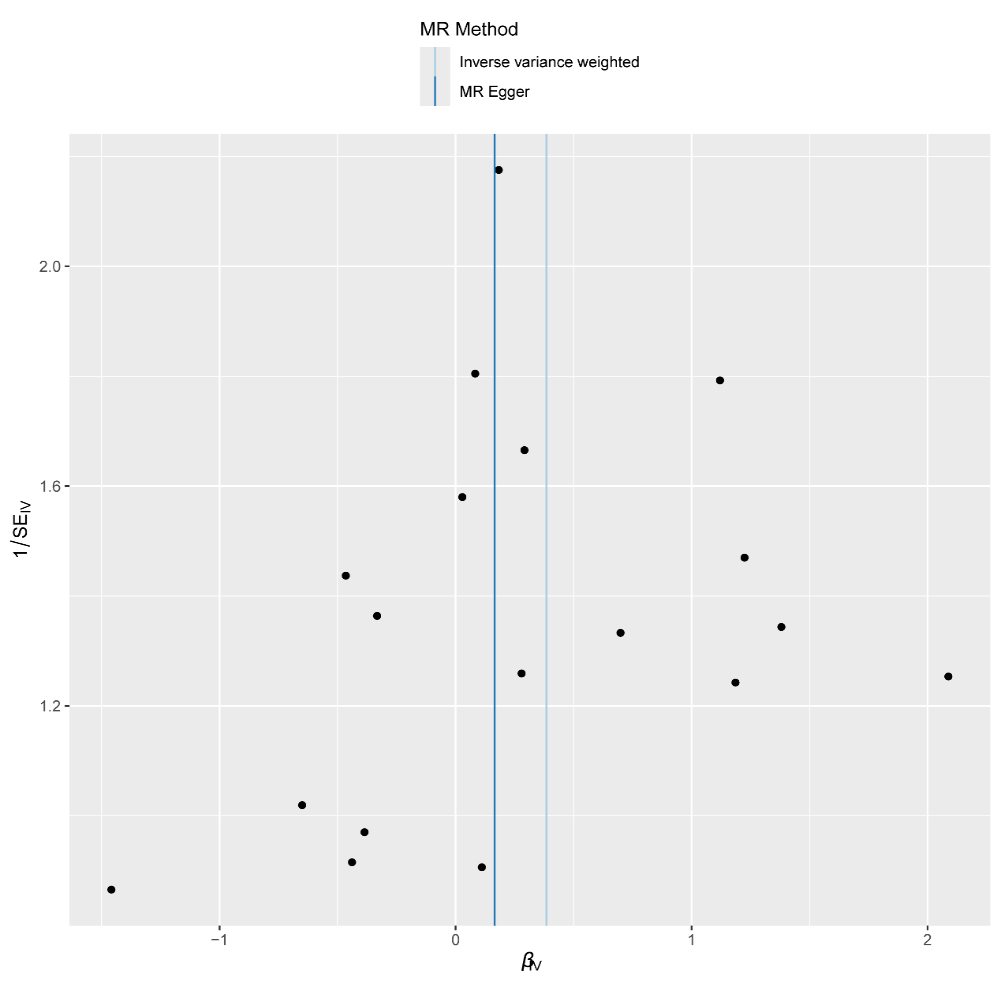


**（A） (B)**


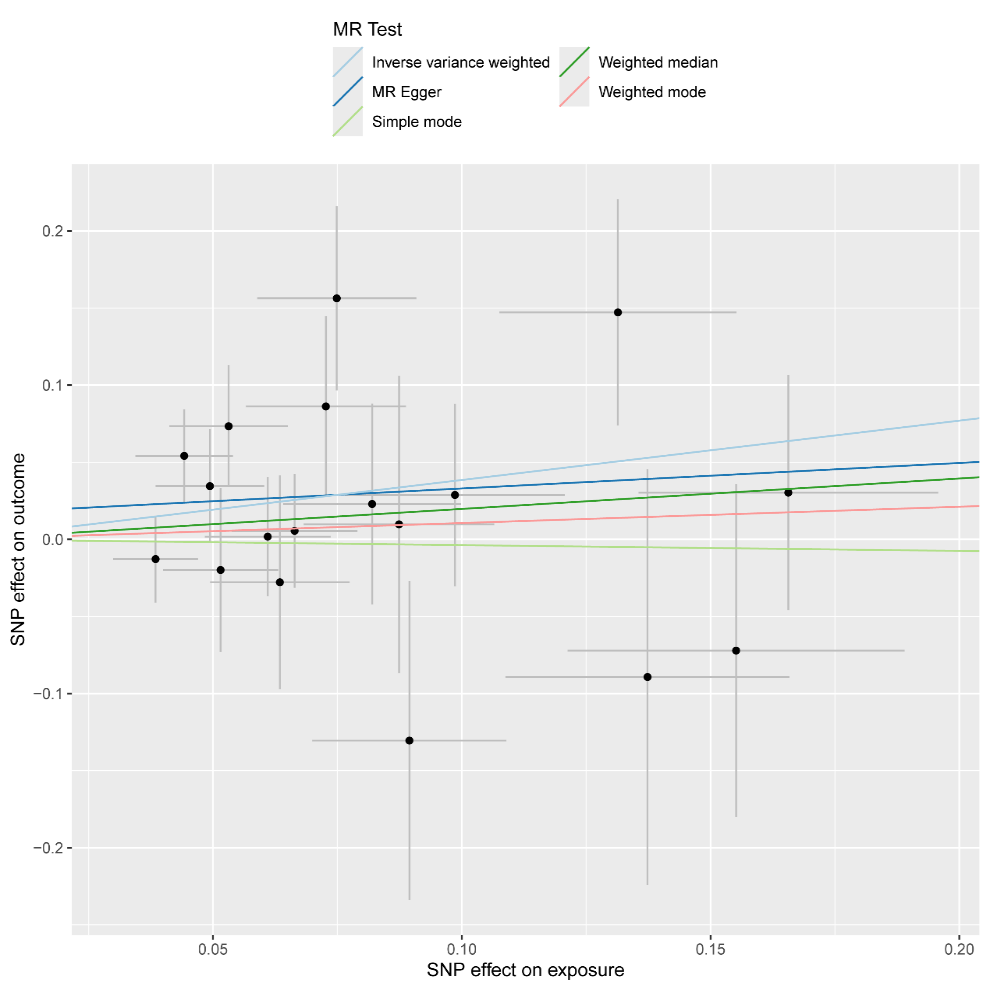

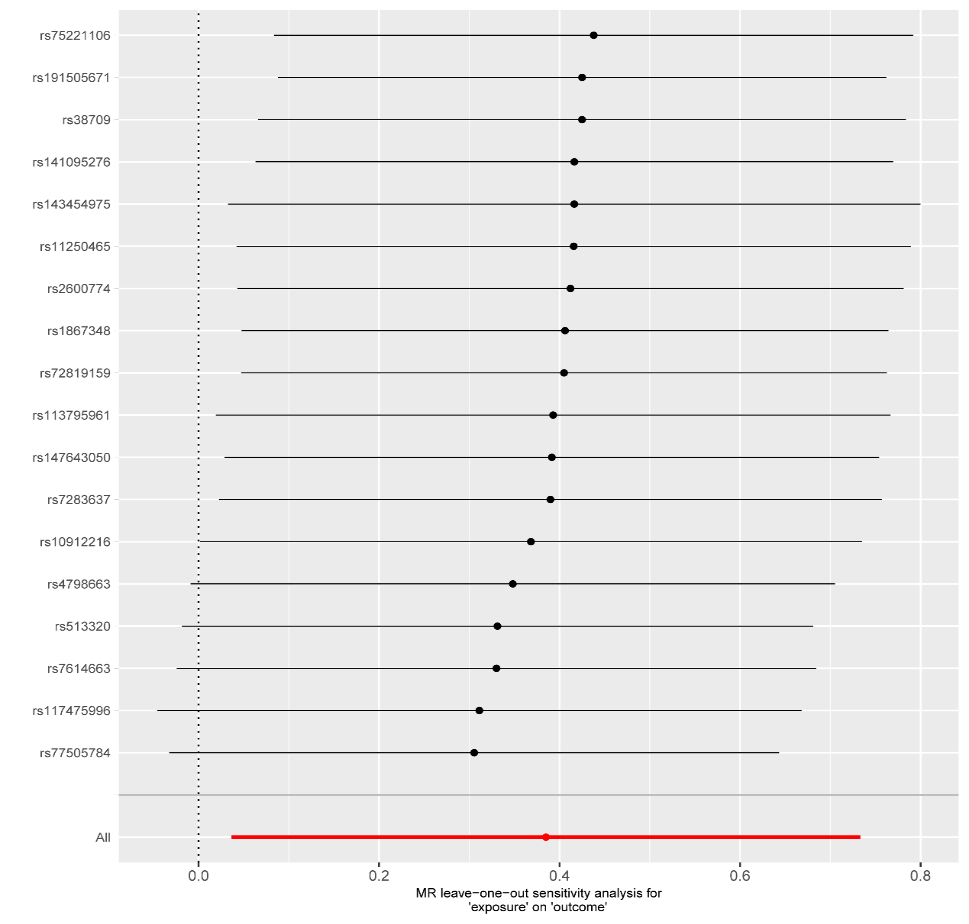


**(C) (D)**

### Faecalicatena lactaris on UC.


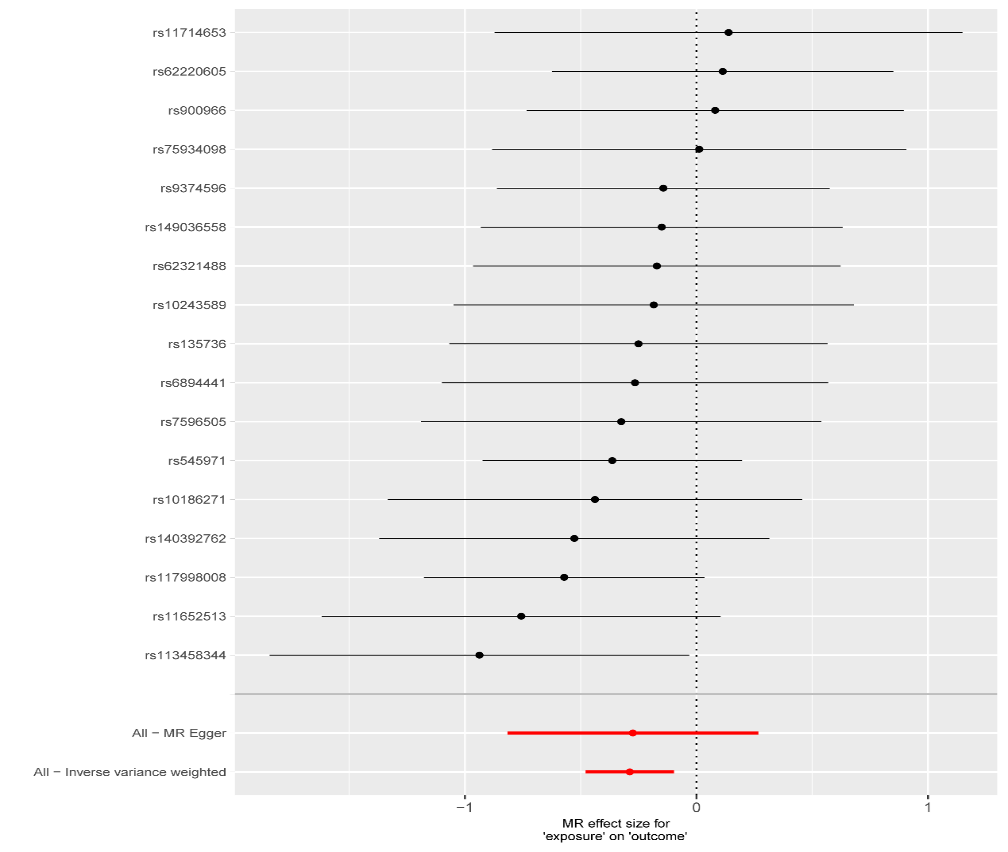

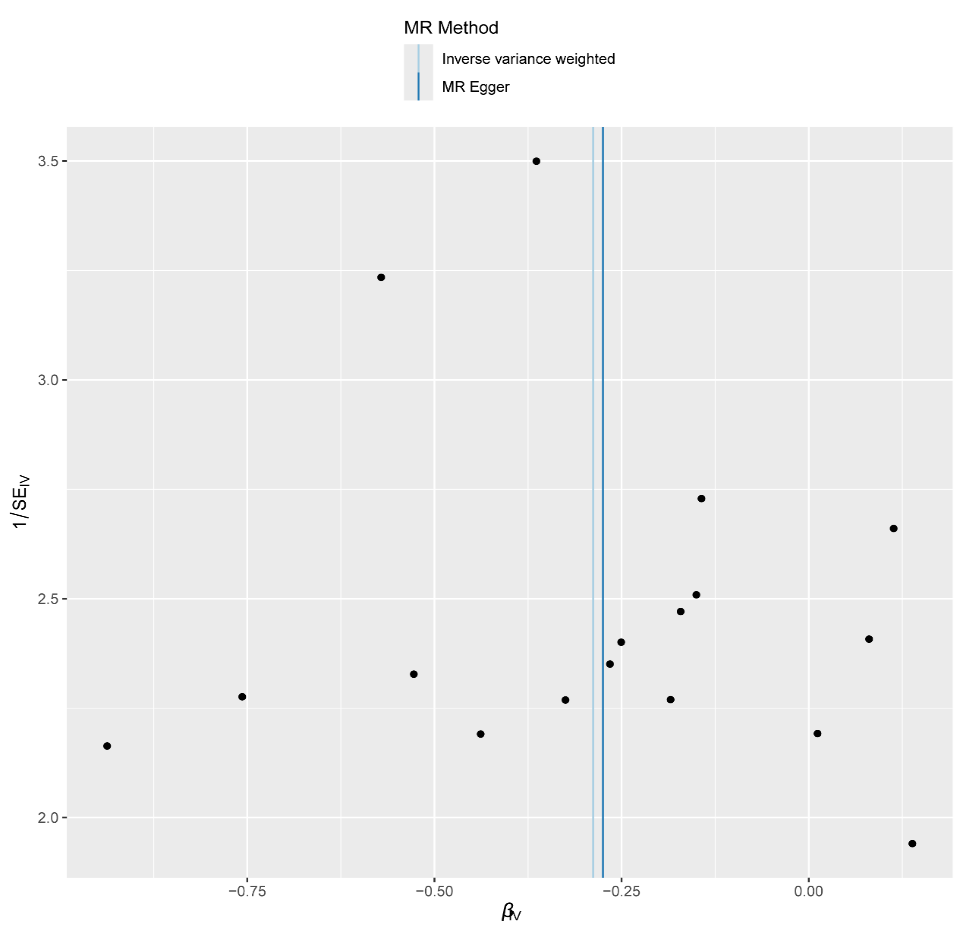


**(A) (B)**


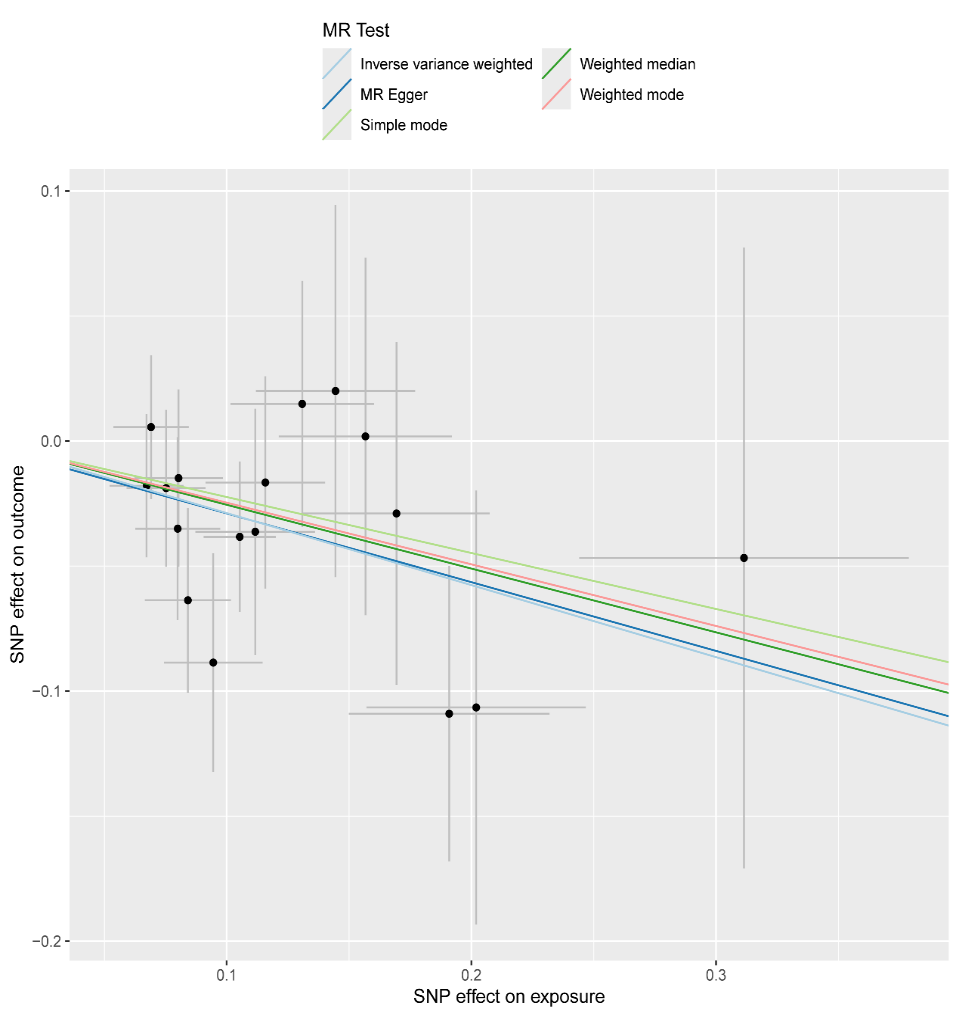

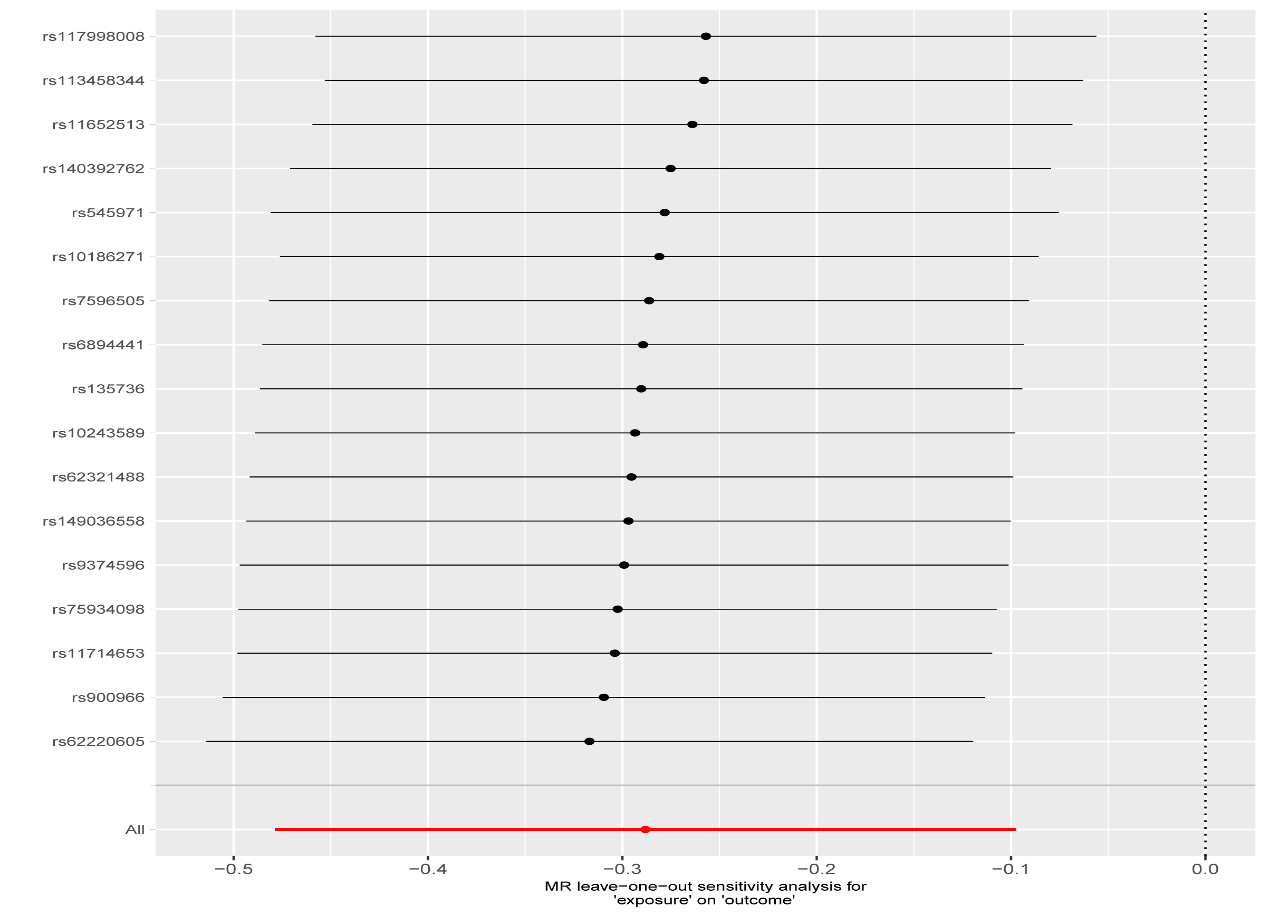


**(C) (D)**

### Faecalicatena sp002161355 on UC.


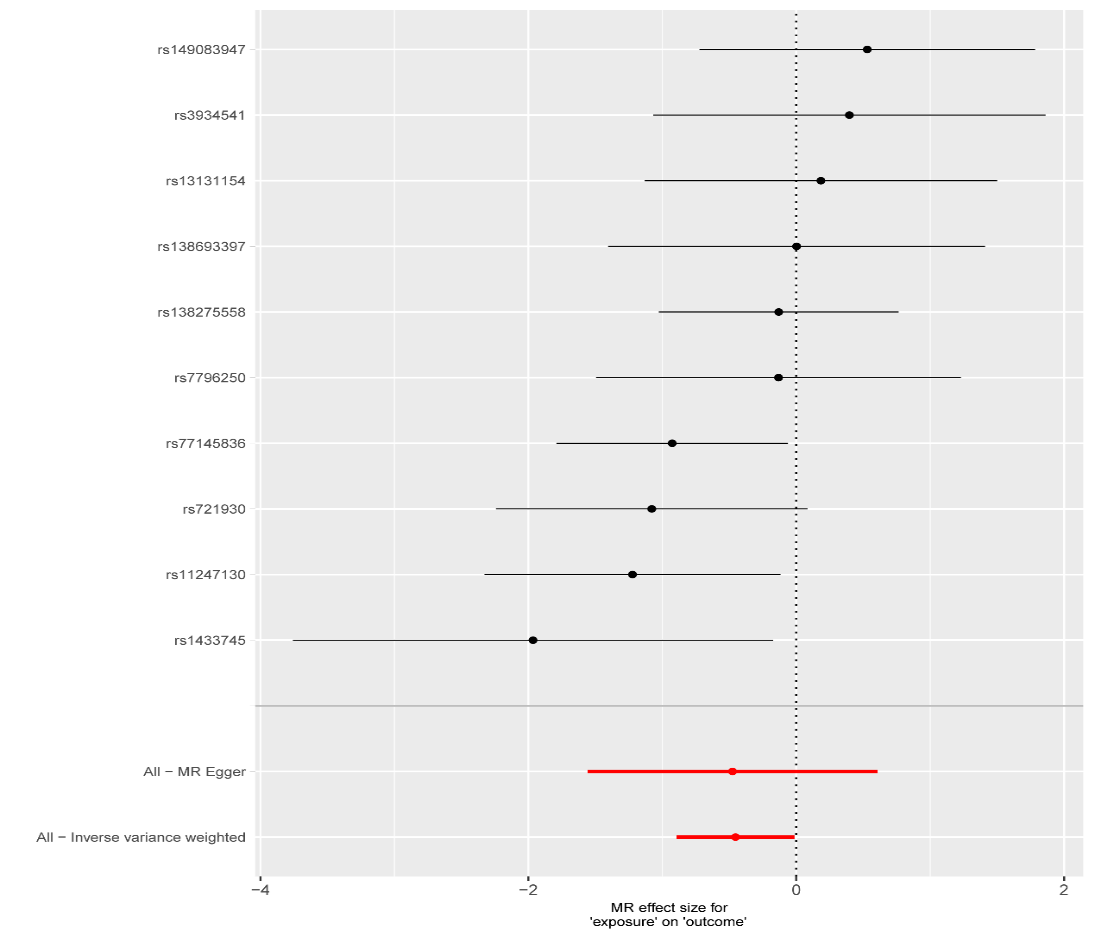

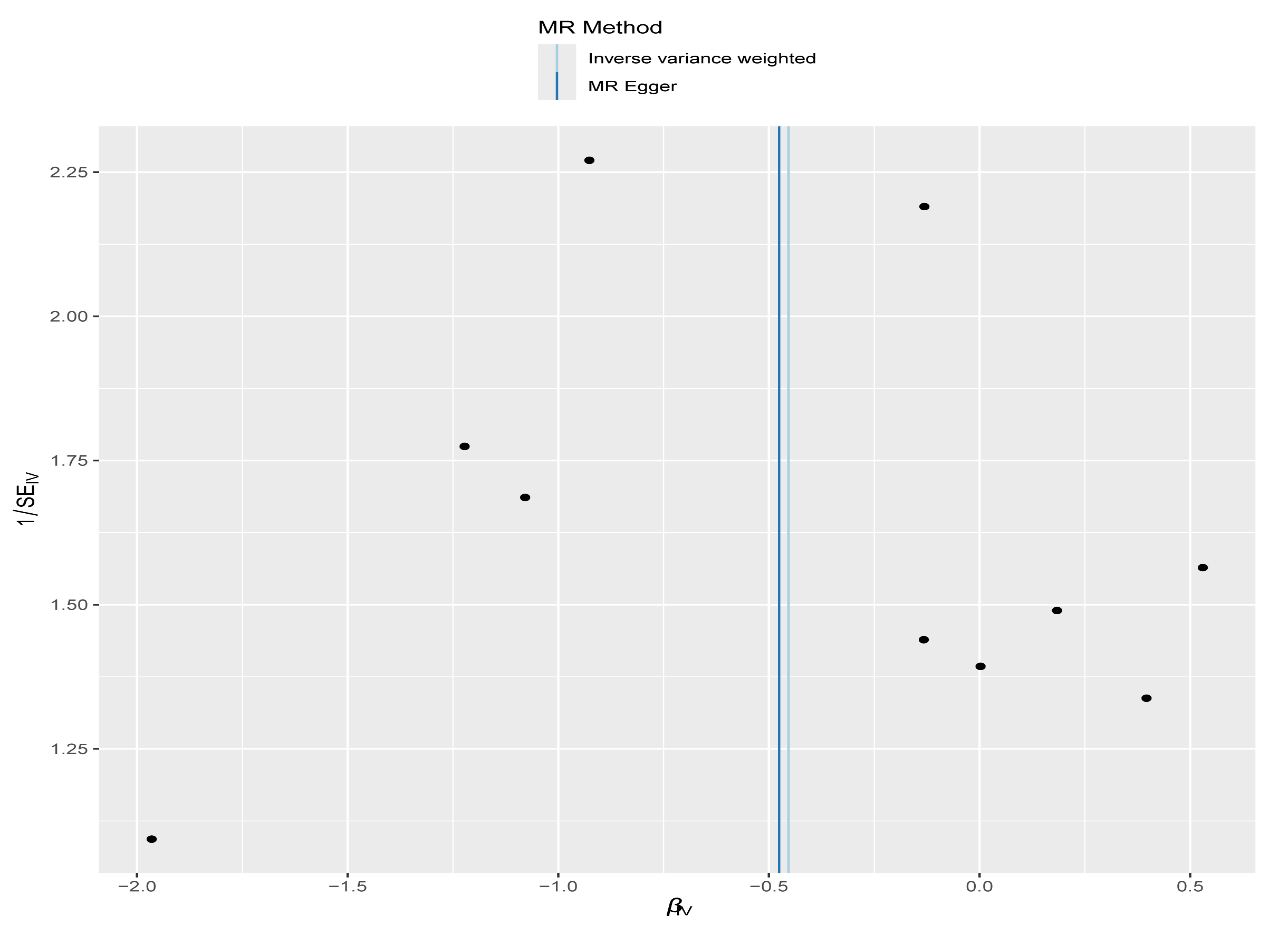


**(A) (B)**


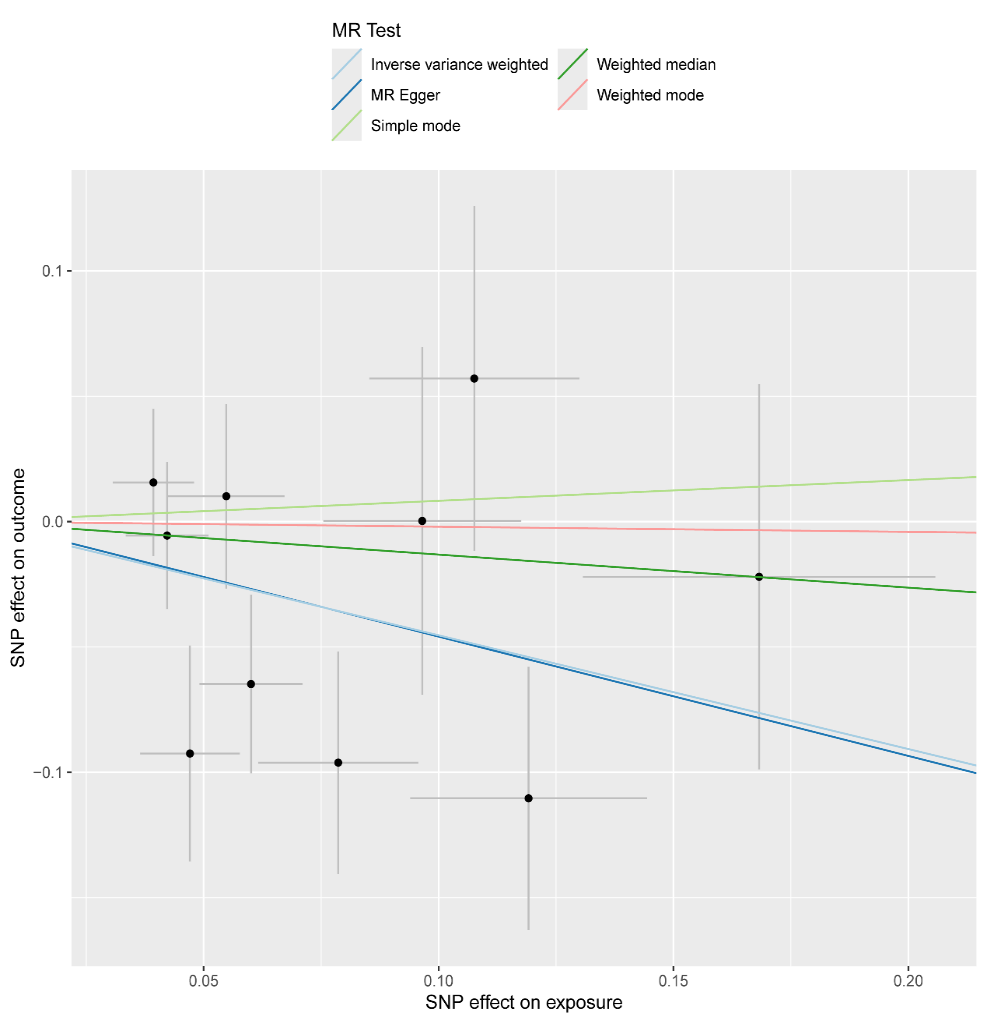

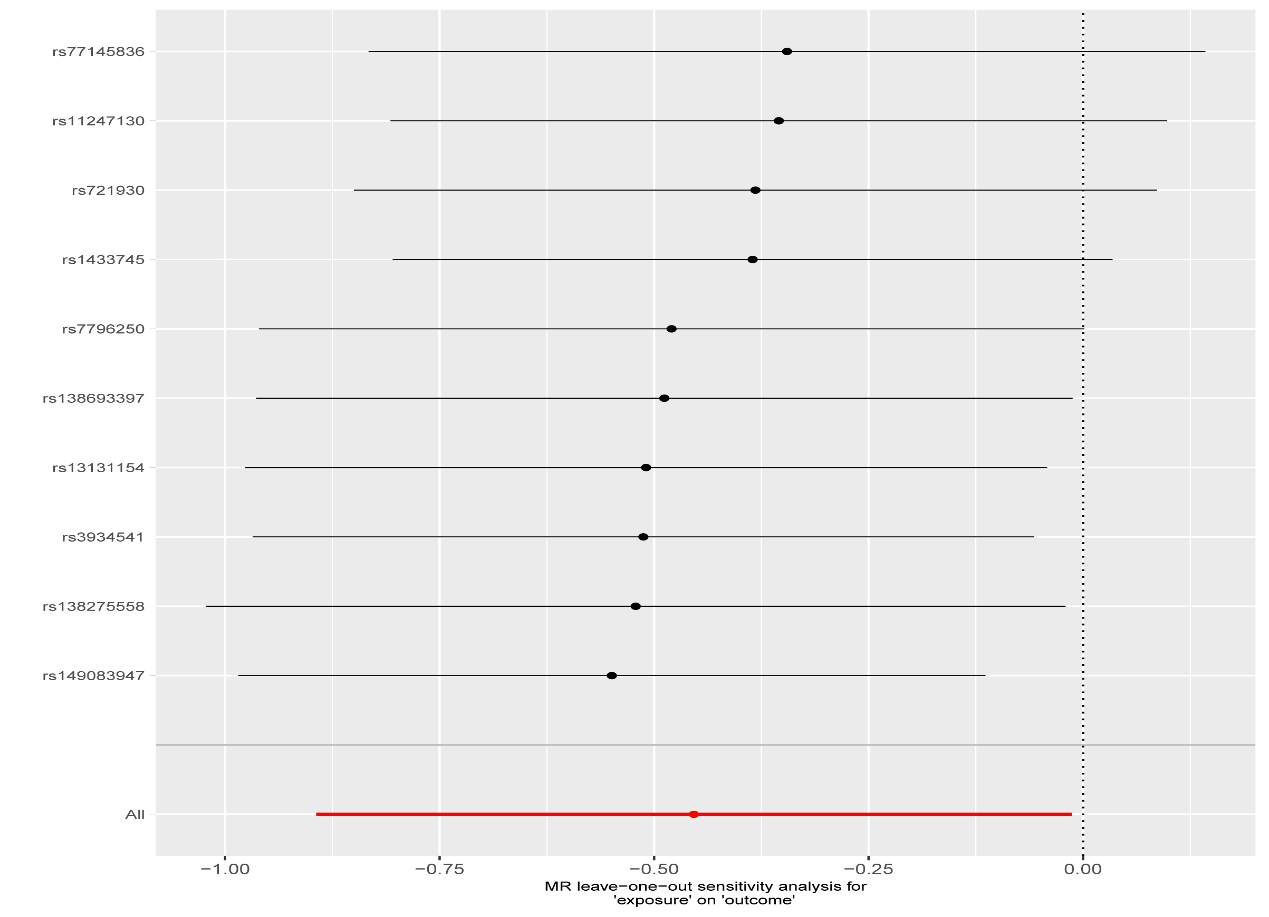


**(C) (D)**

### Lentimicrobiaceae on UC.


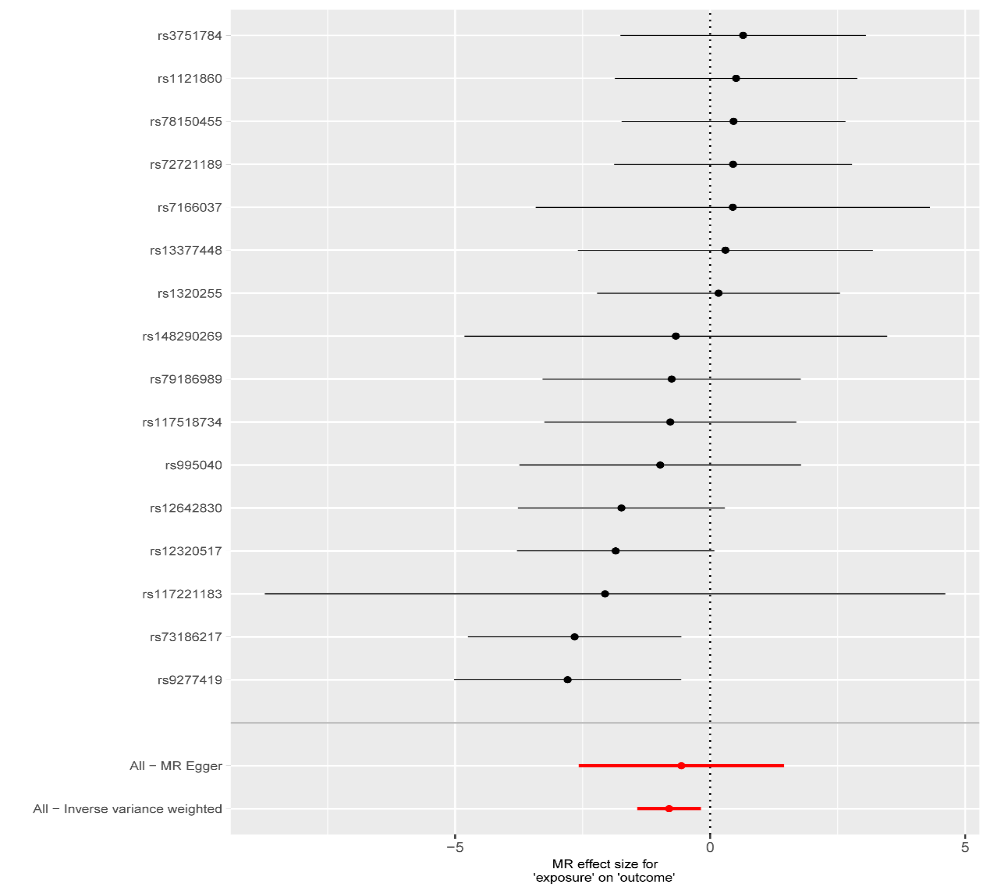

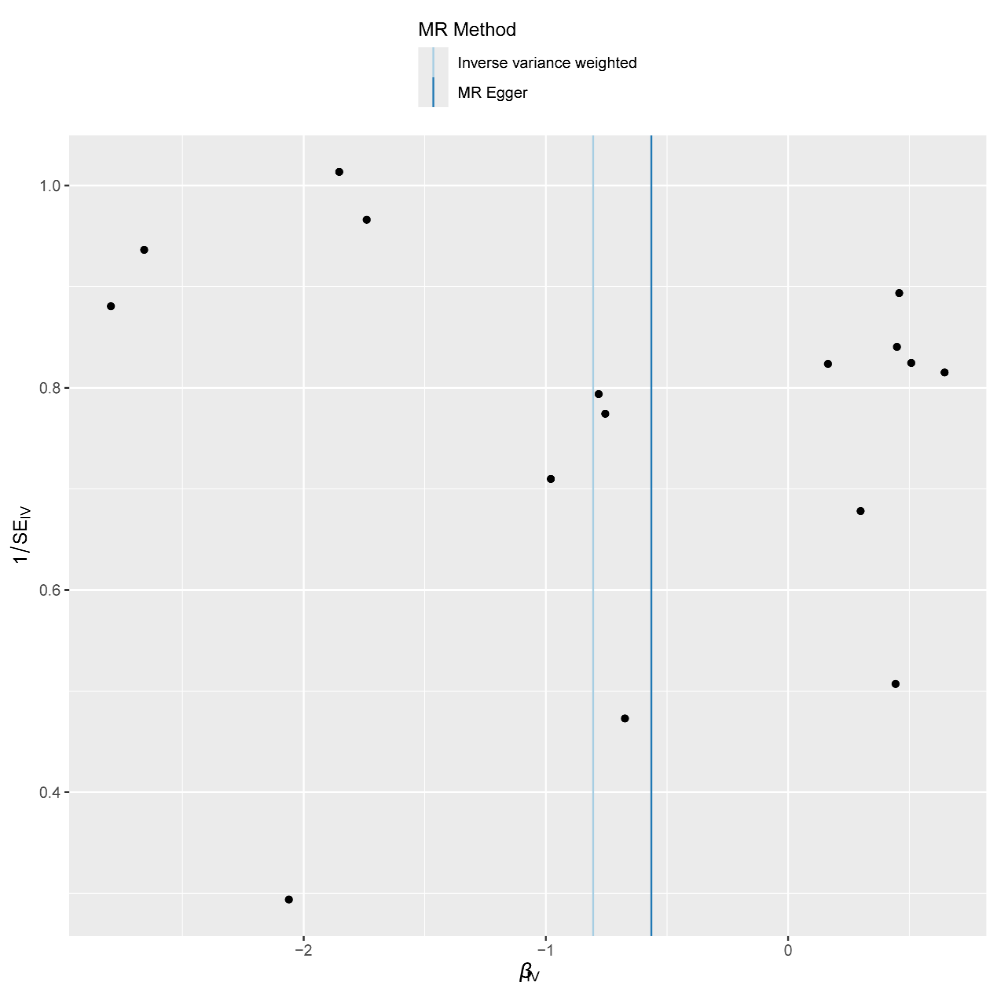


**(A) (B)**


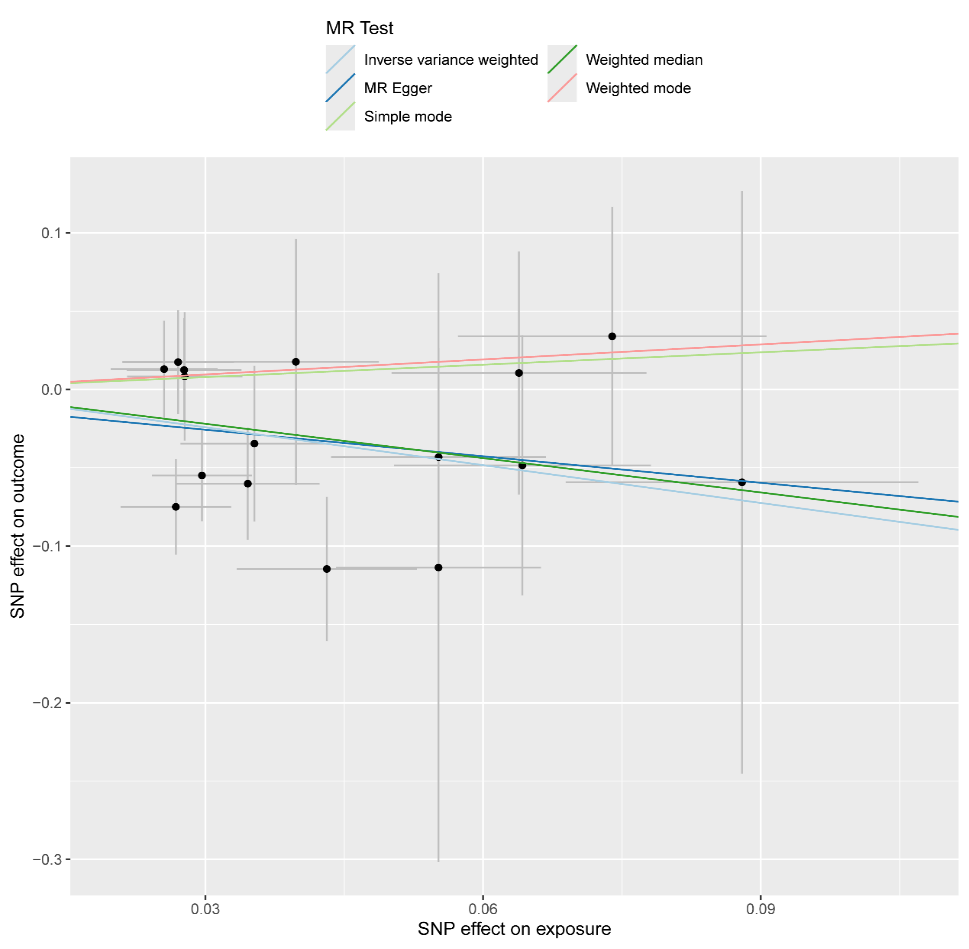

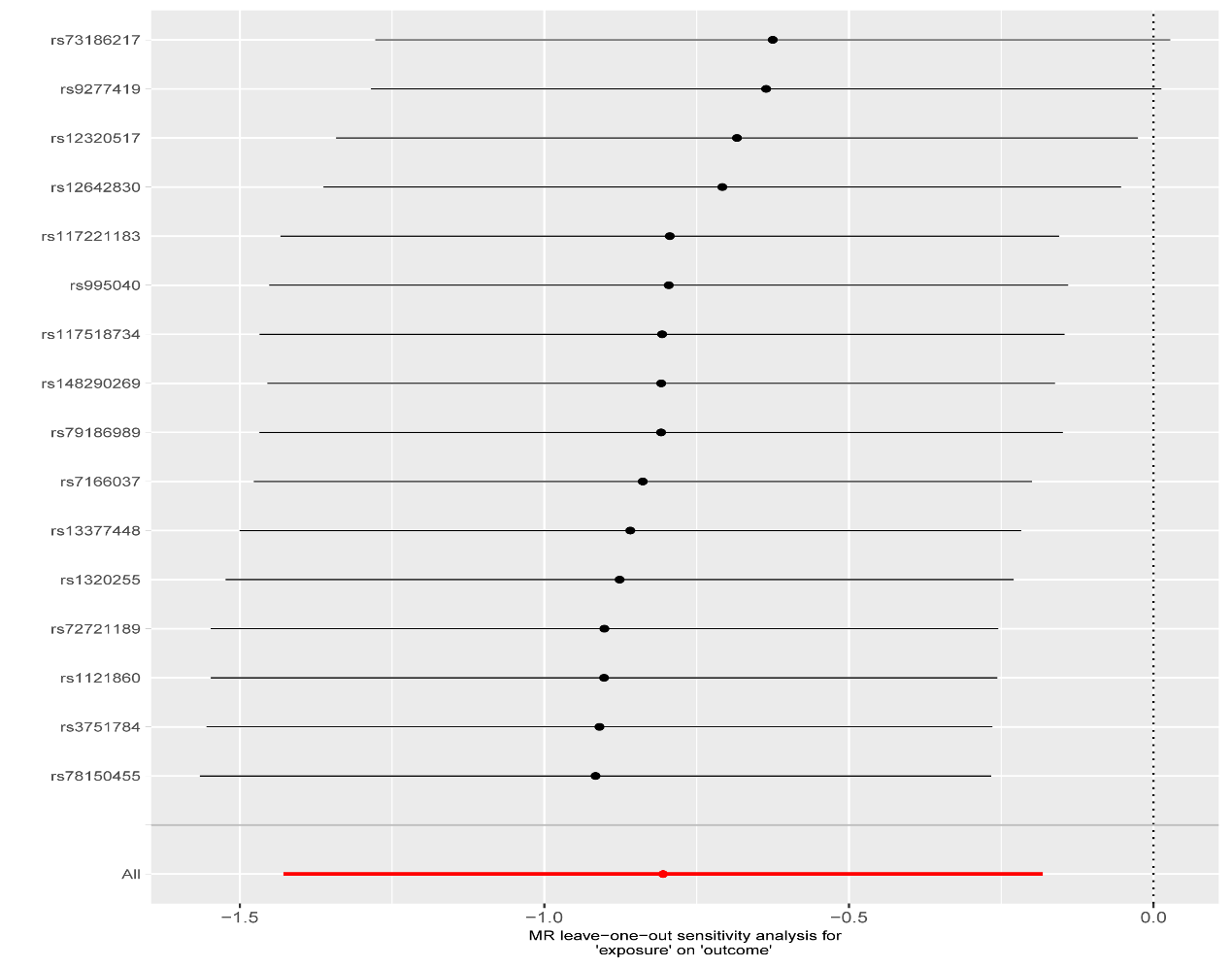


**(C) (D)**

### Megasphaera sp900066485 on UC.


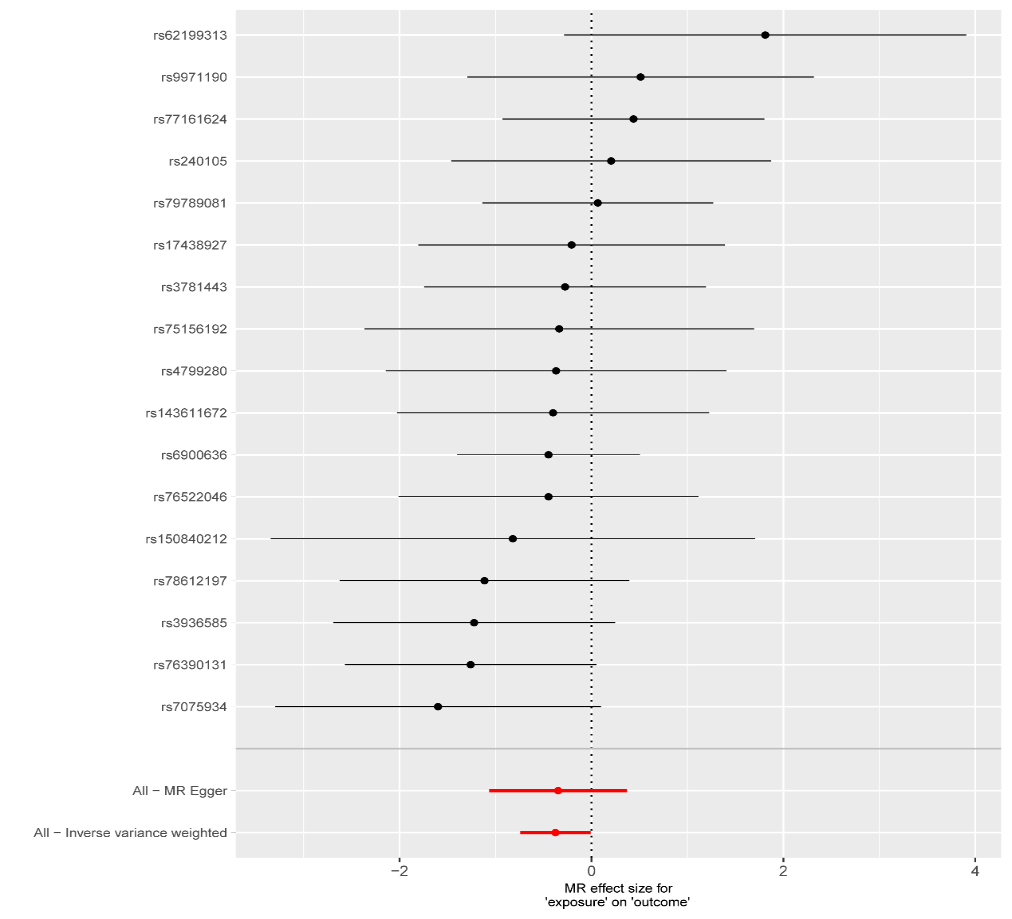

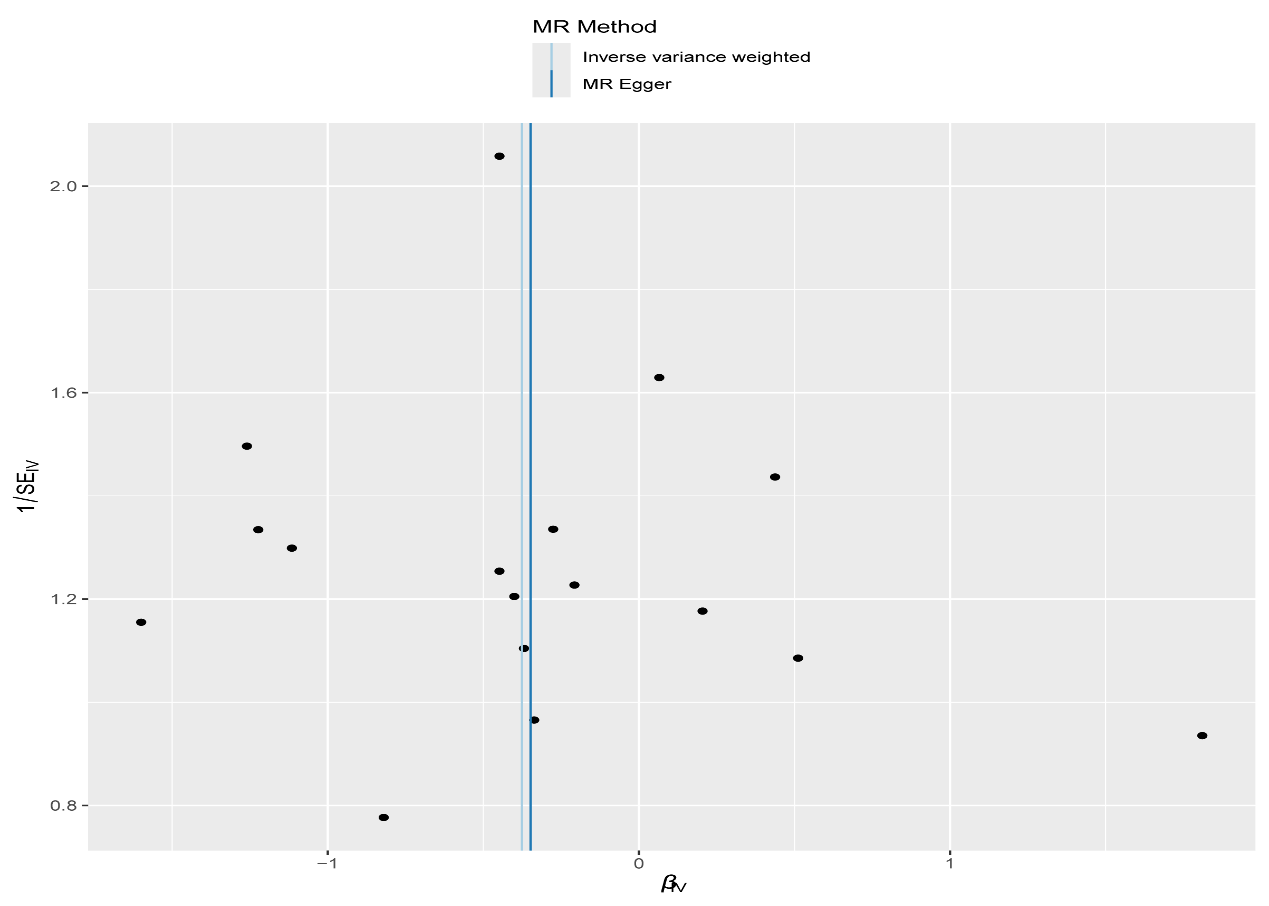


**(A) (B)**


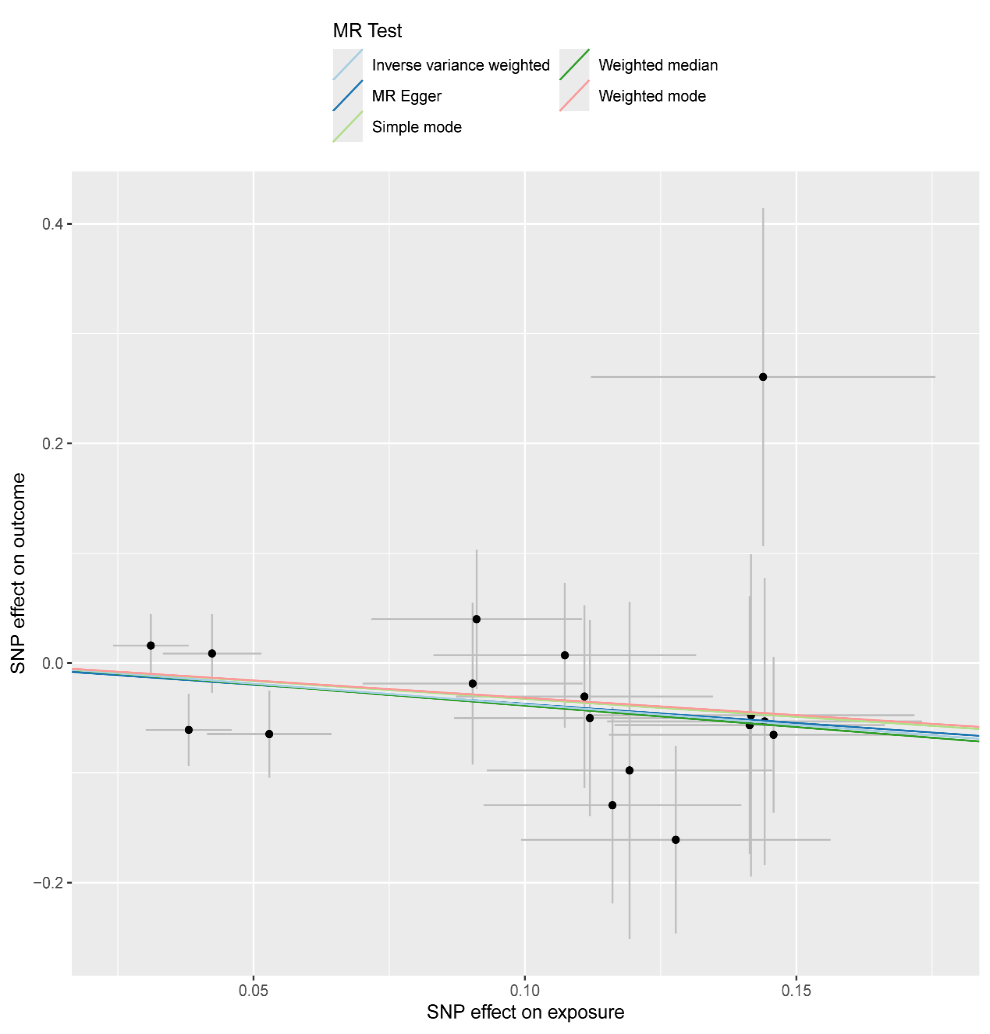

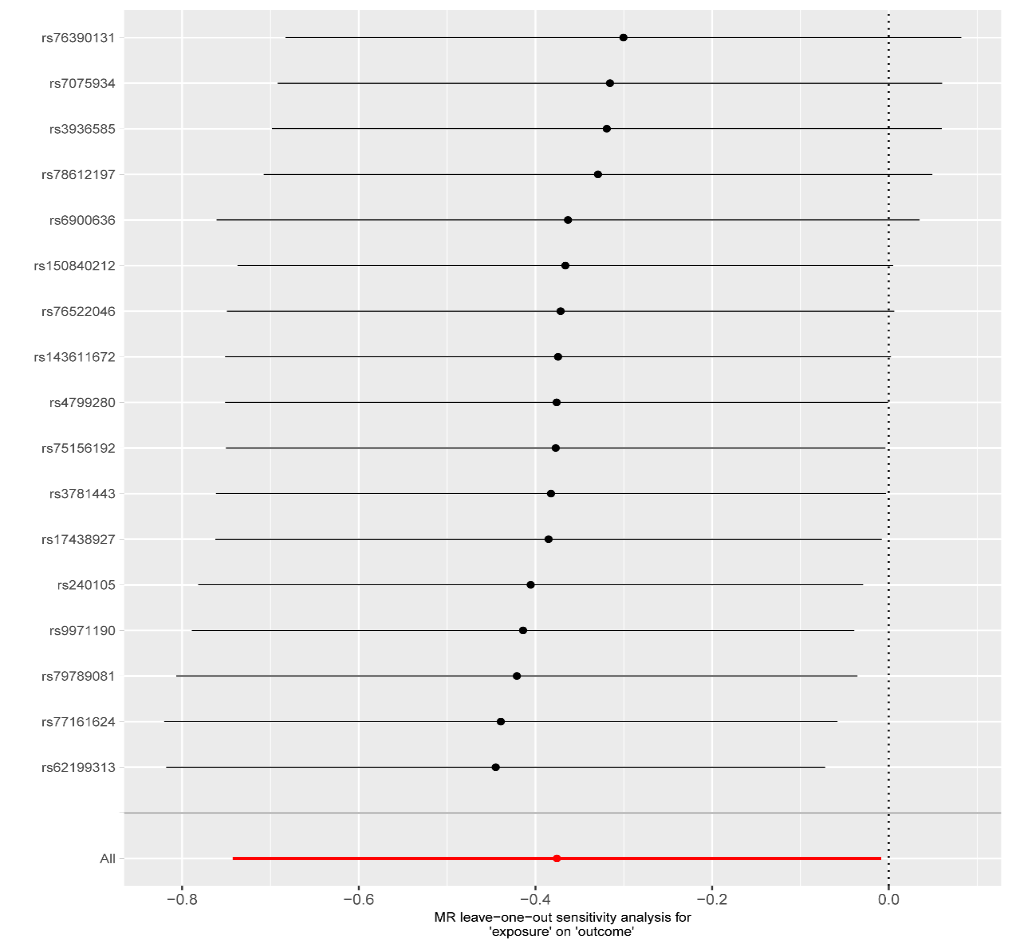


**(C) (D)**

### NK4A144 on UC.


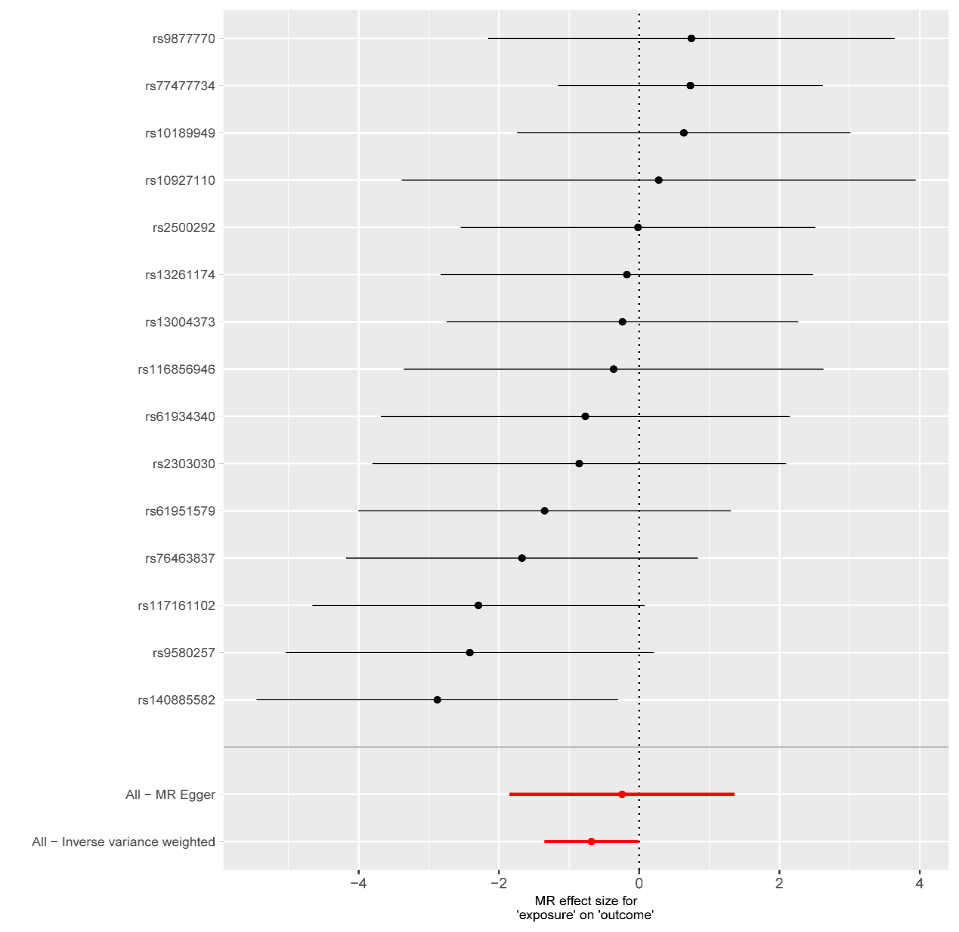

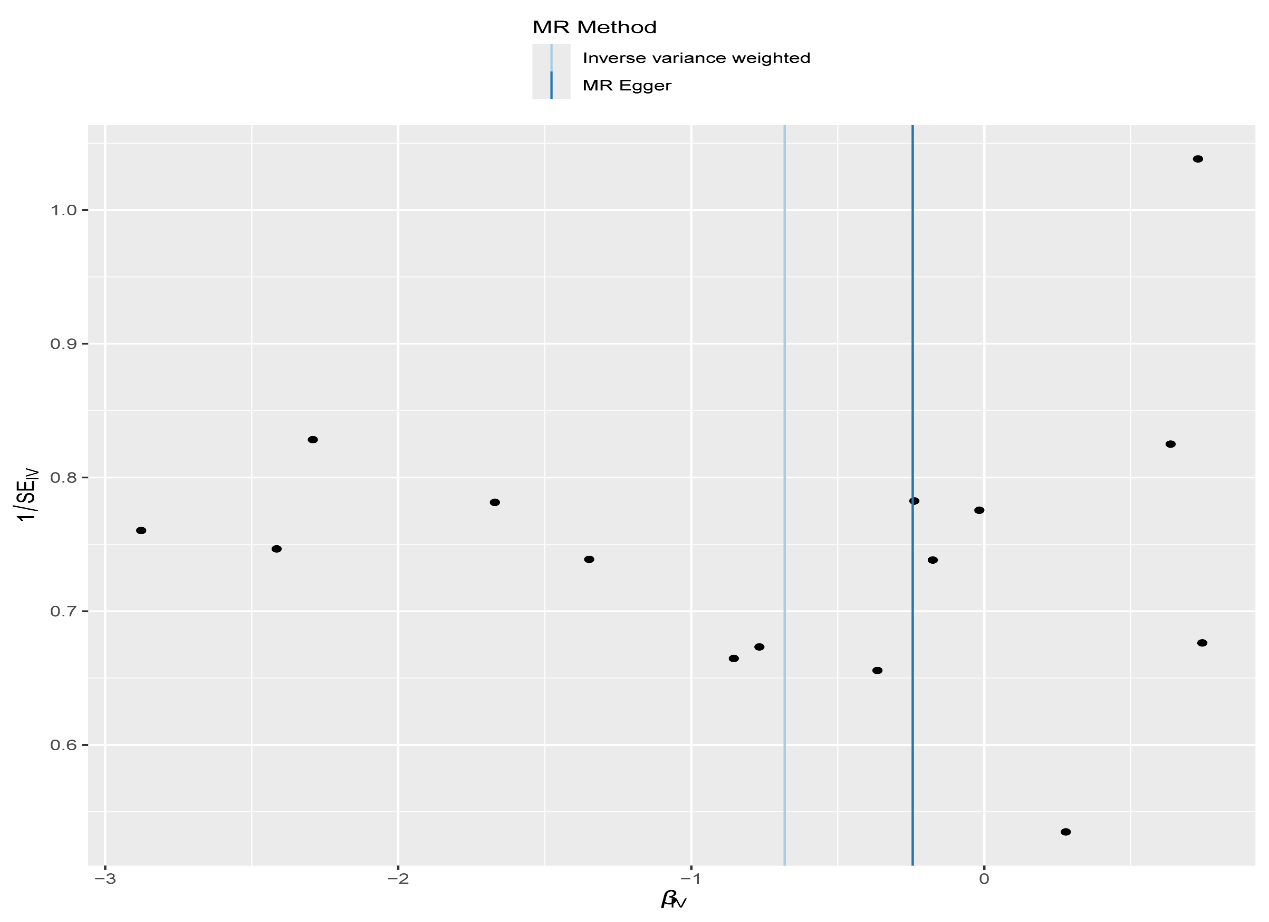


**(A) (B)**


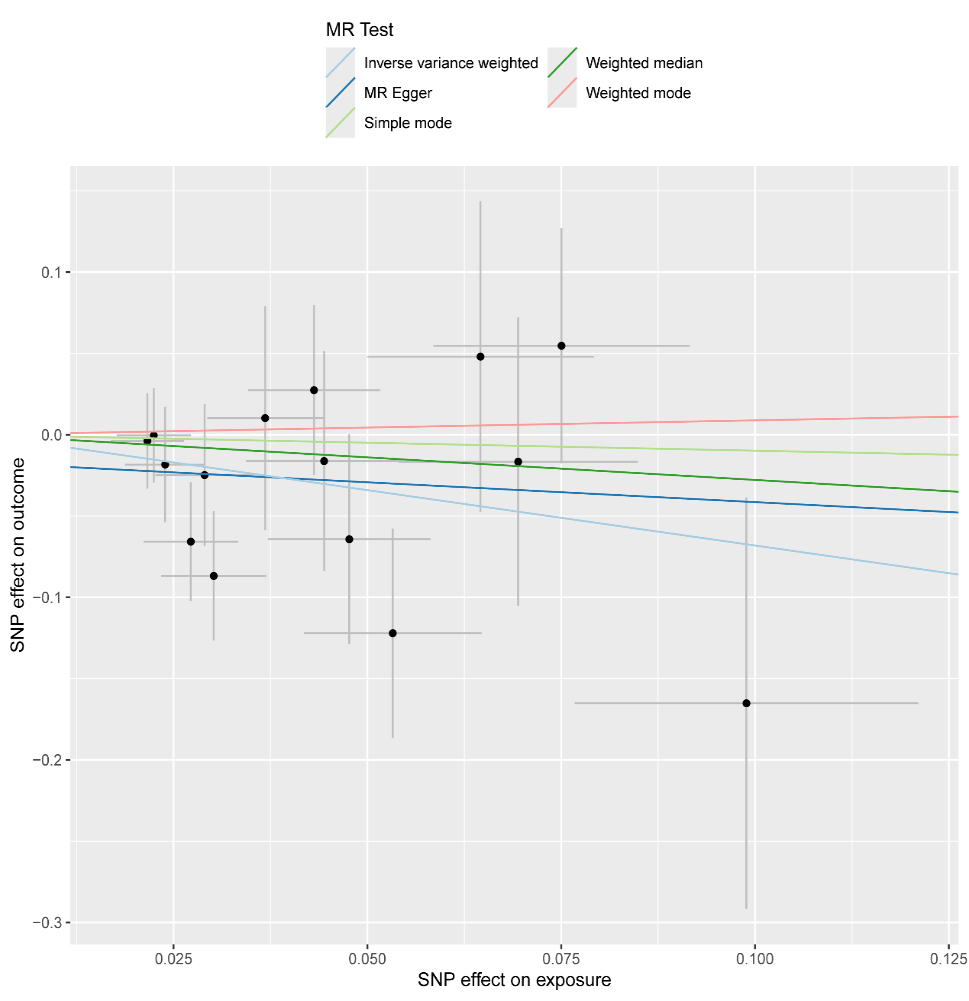

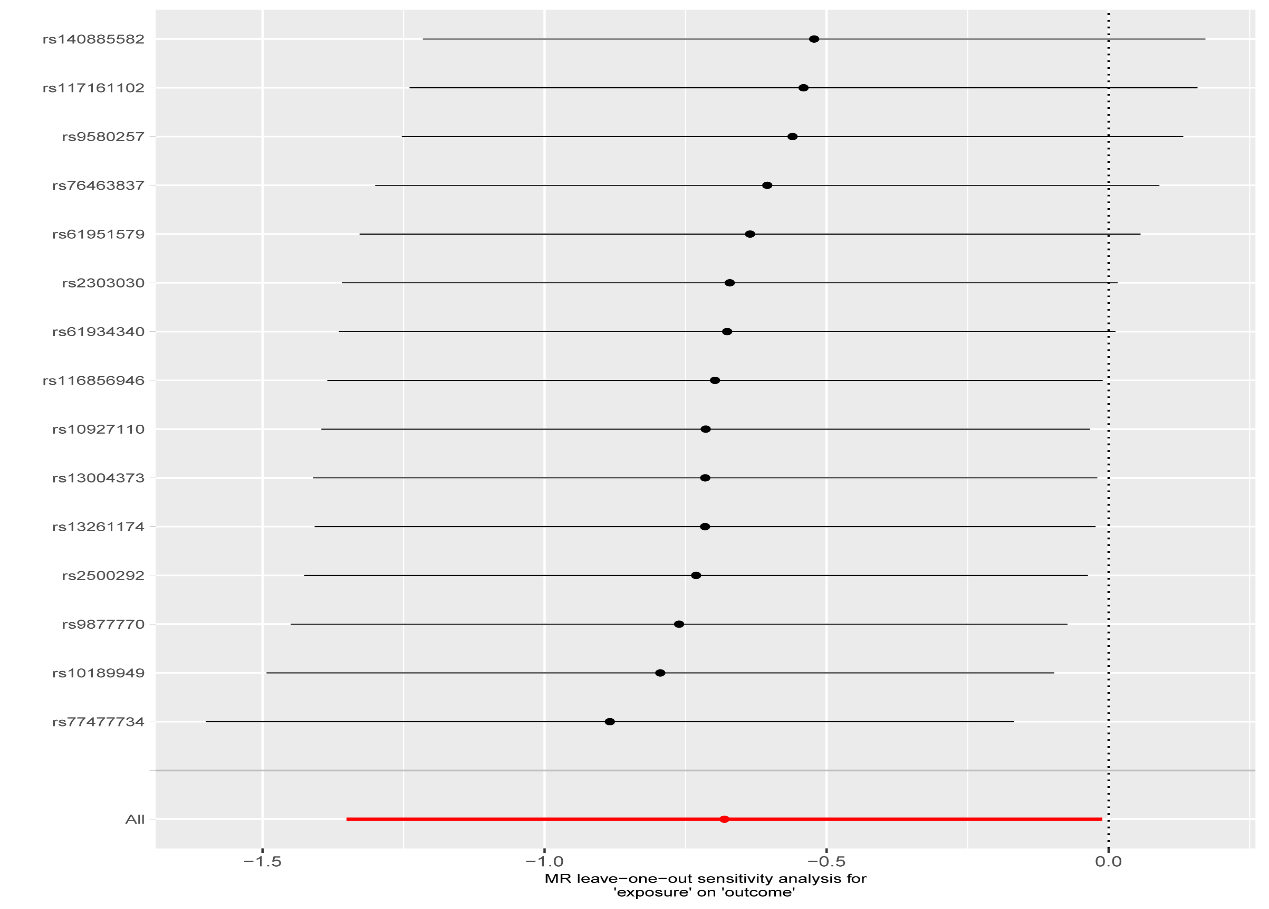


**(C) (D)**

### Provencibacterium massiliense on UC.


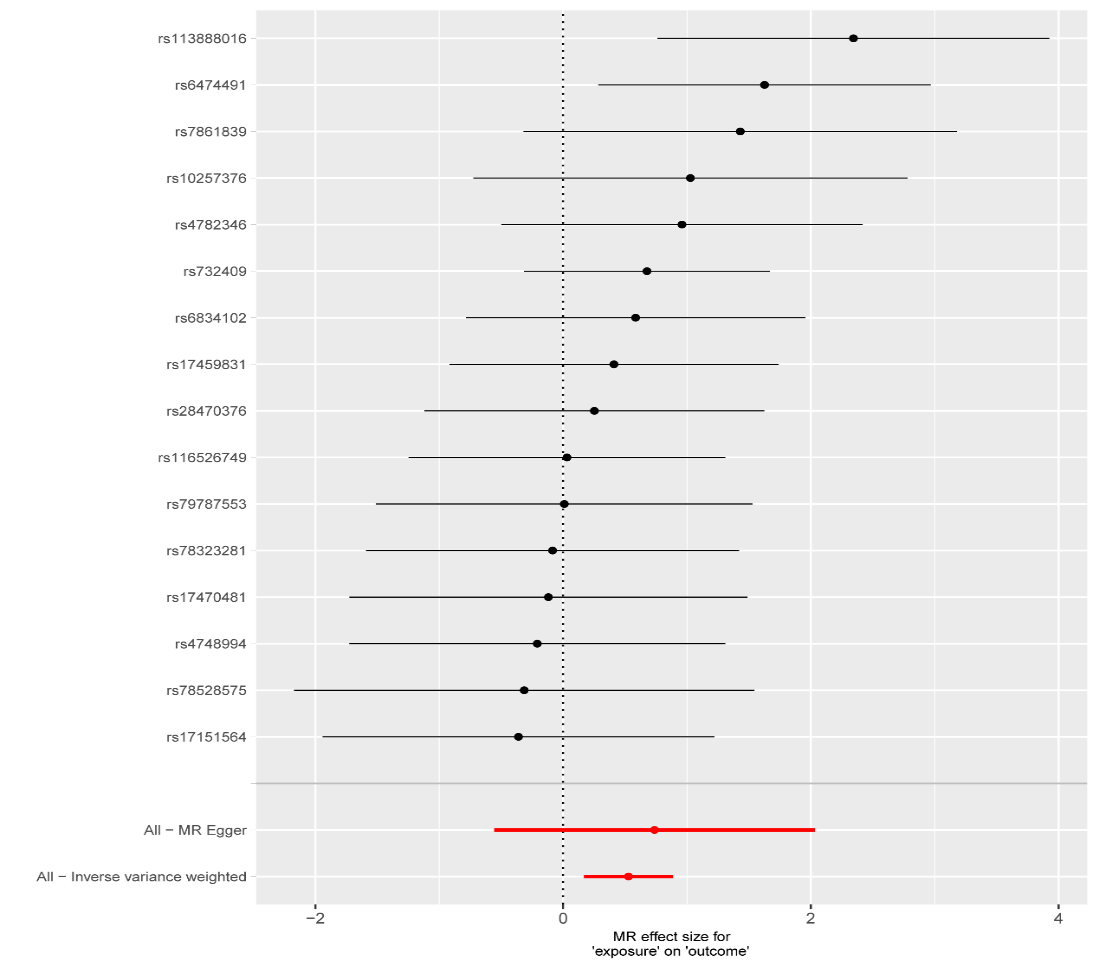

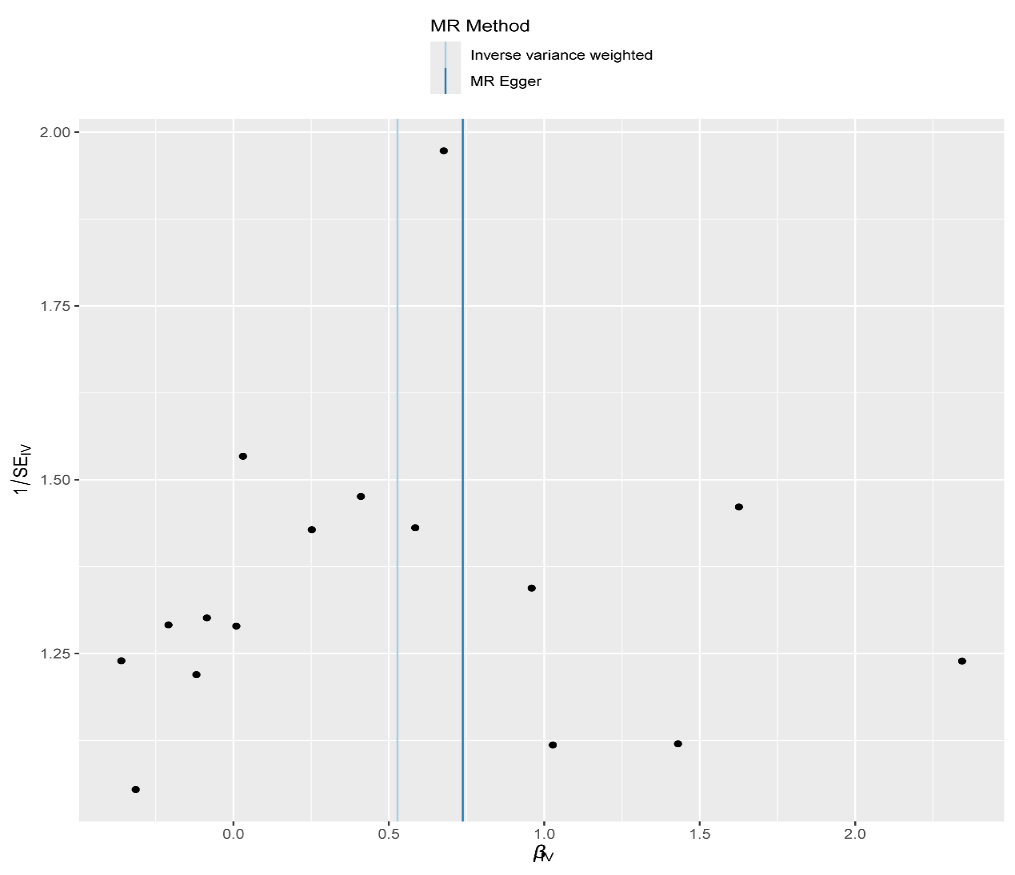


**(A) (B)**


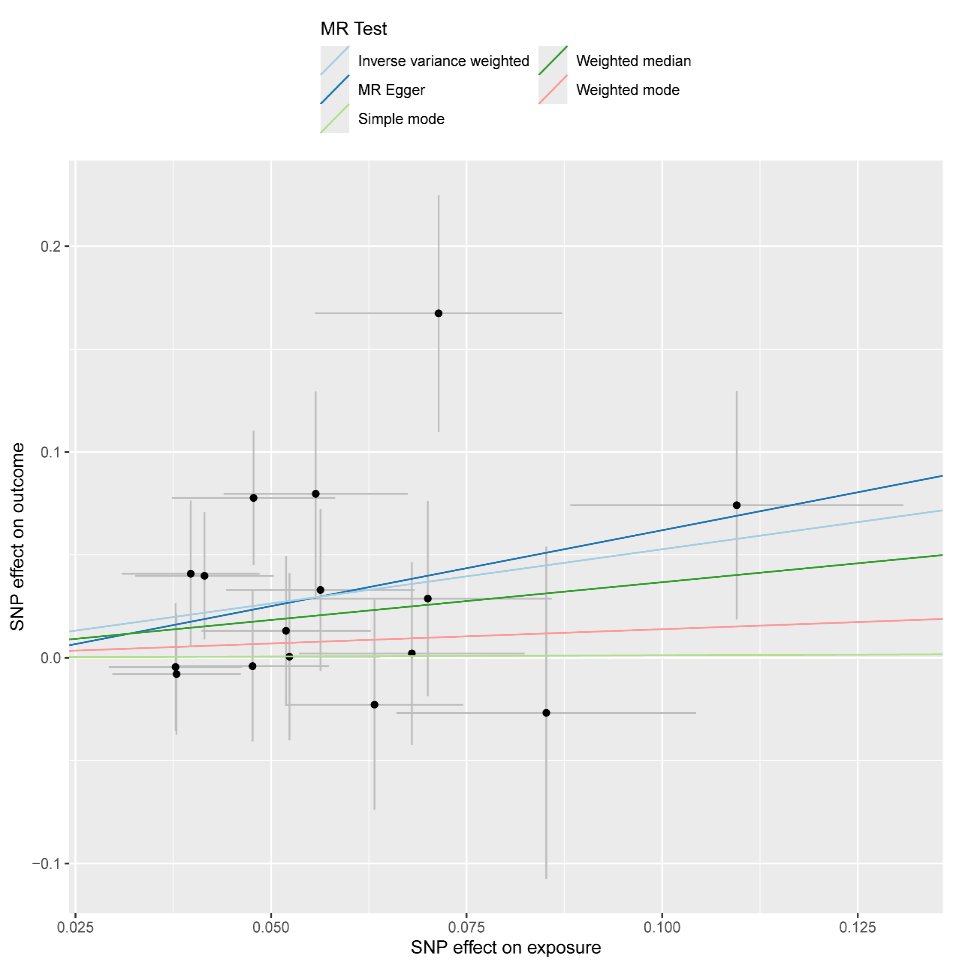

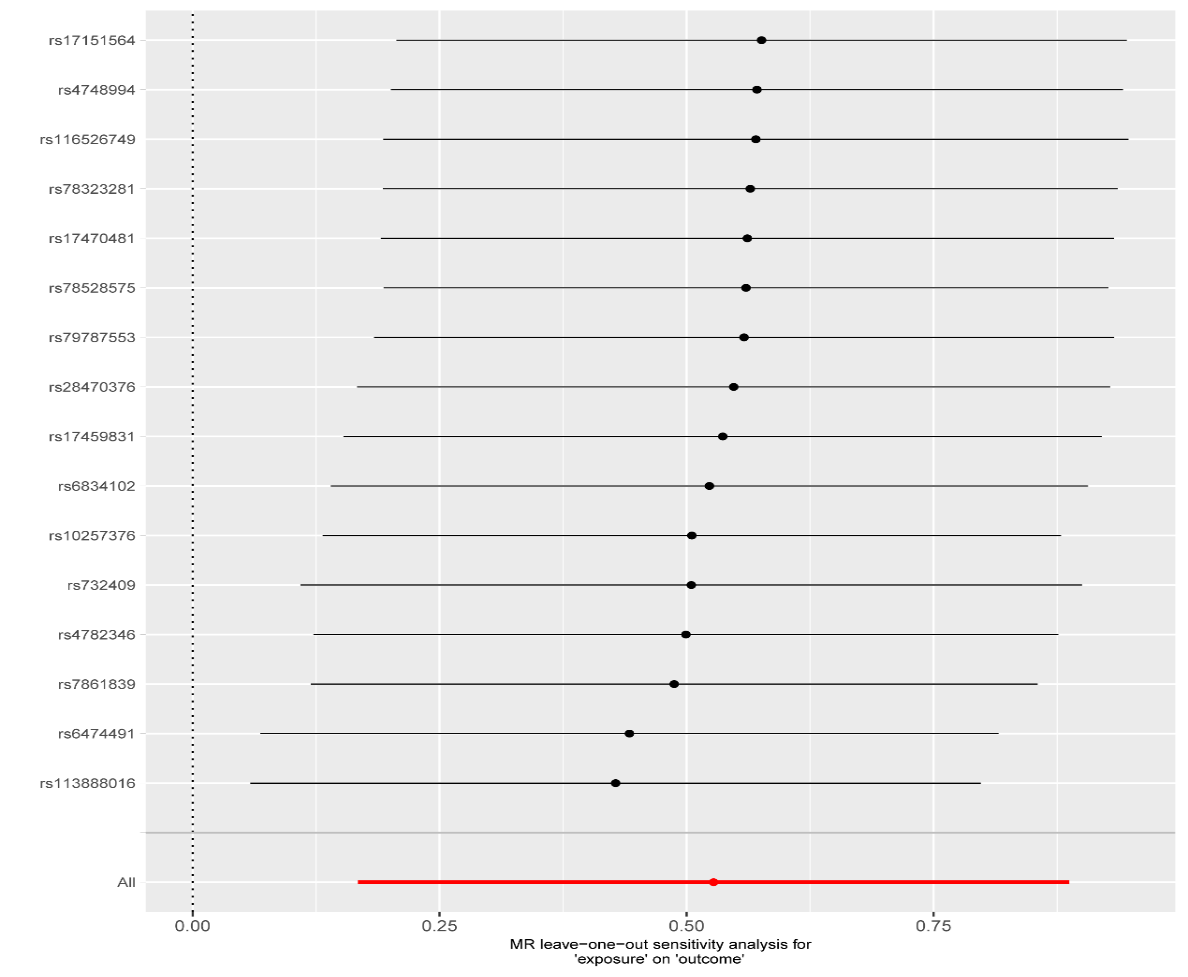


**(C) (D)**

### Provencibacterium on UC.


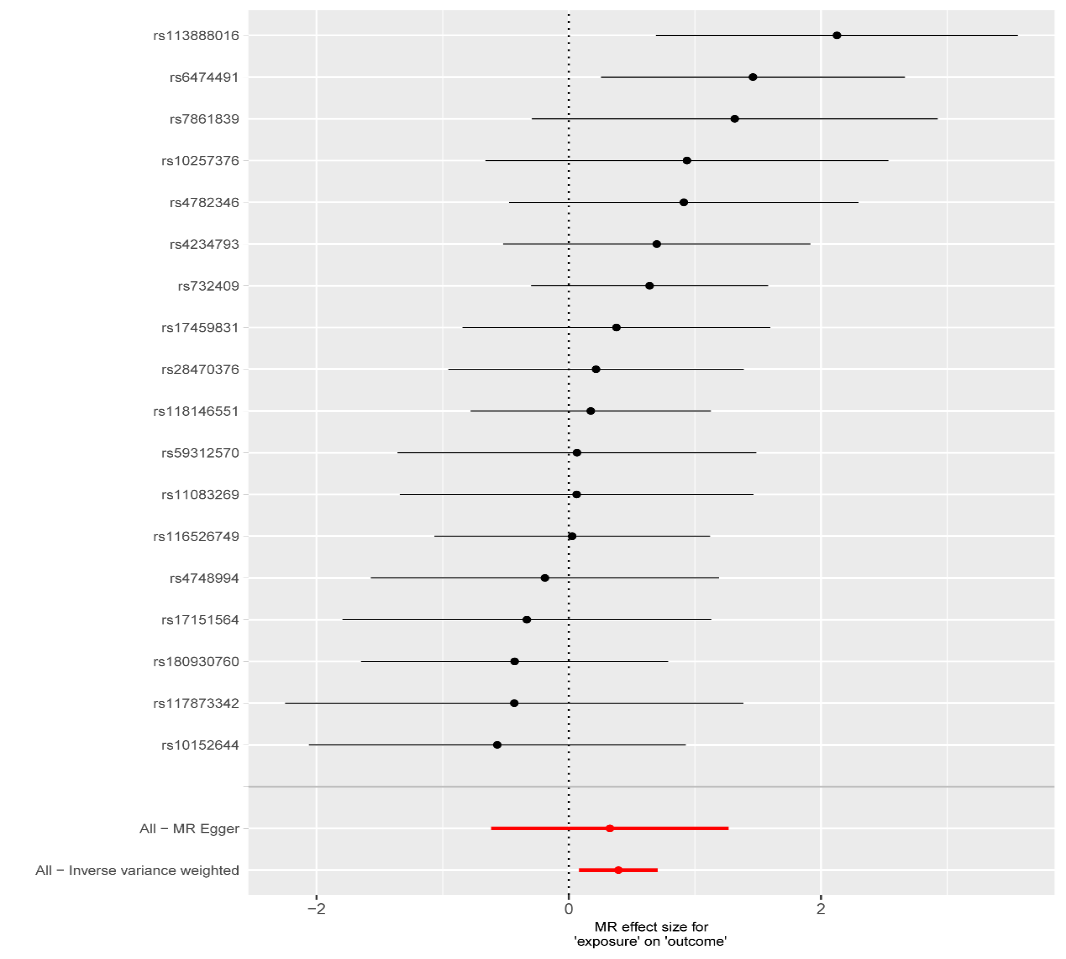

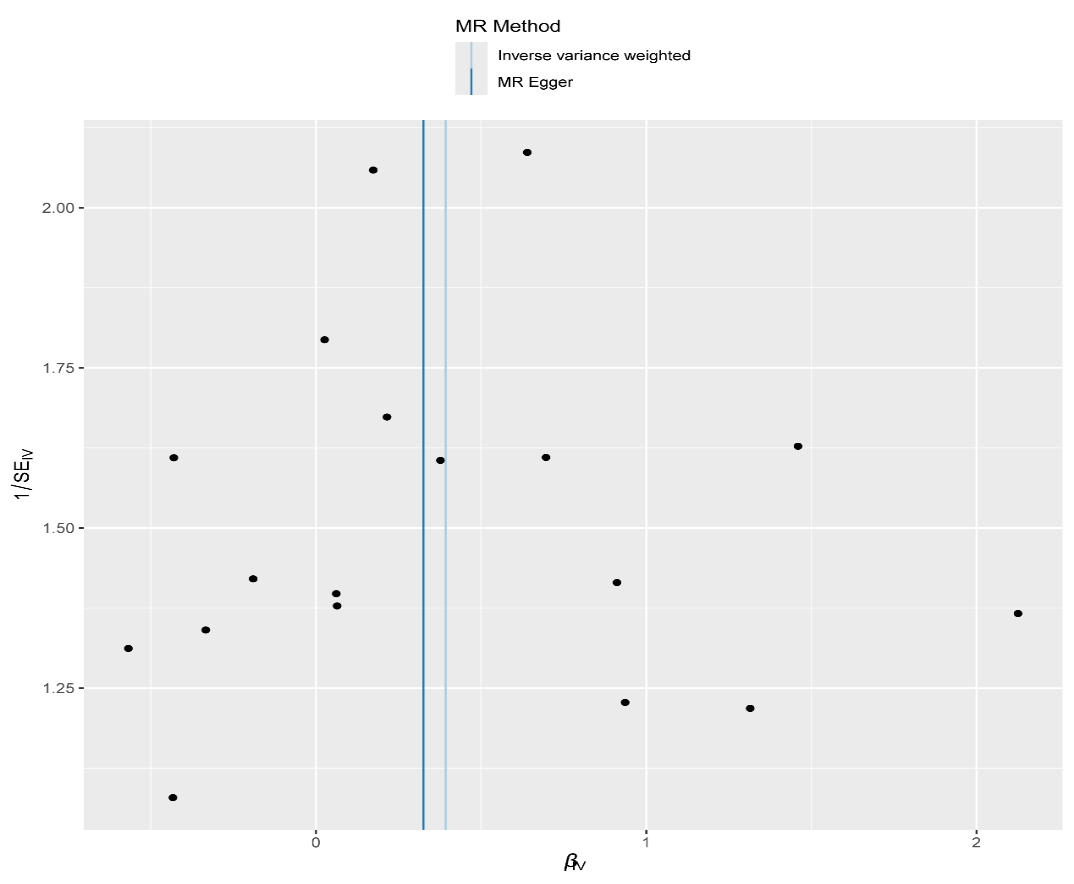


**(A) (B)**


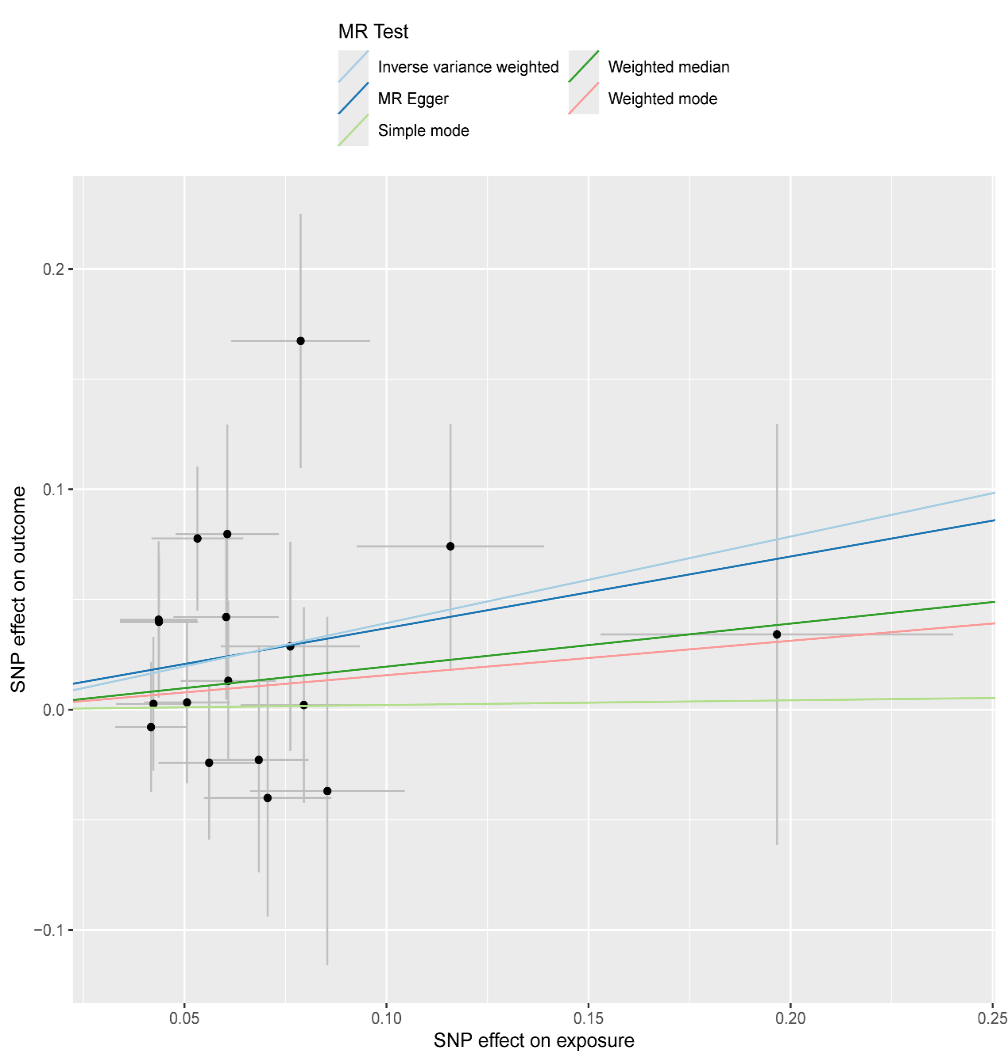

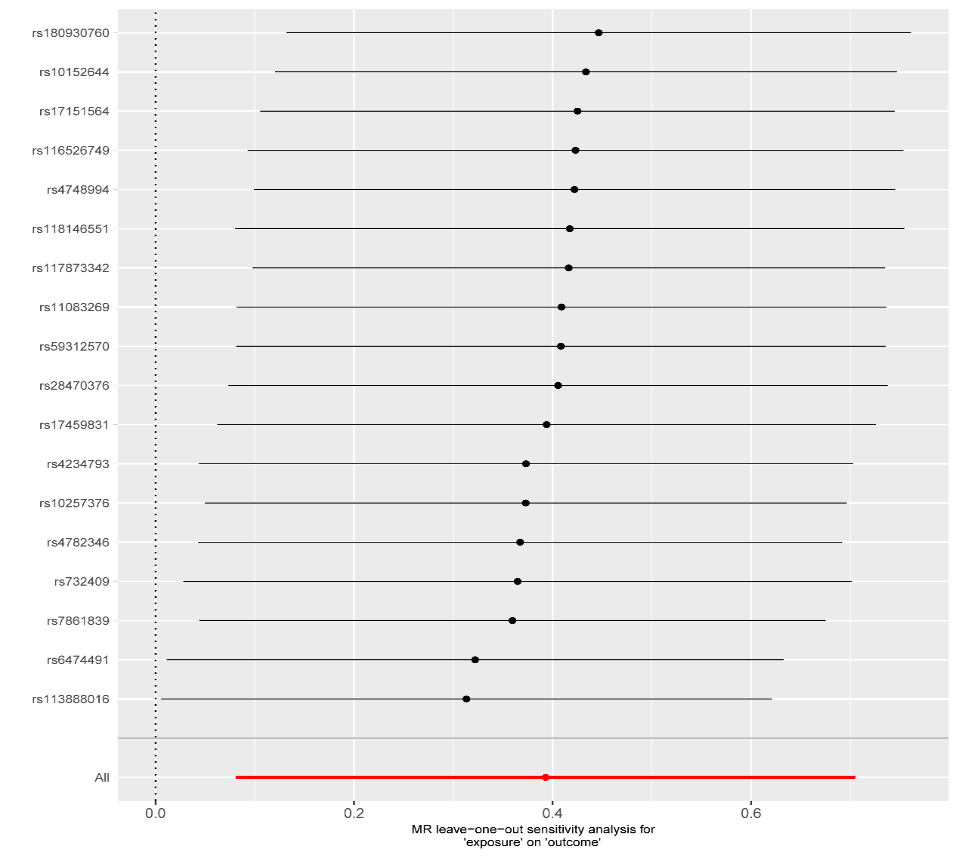


**(C) (D)**

### SAR324 on UC.


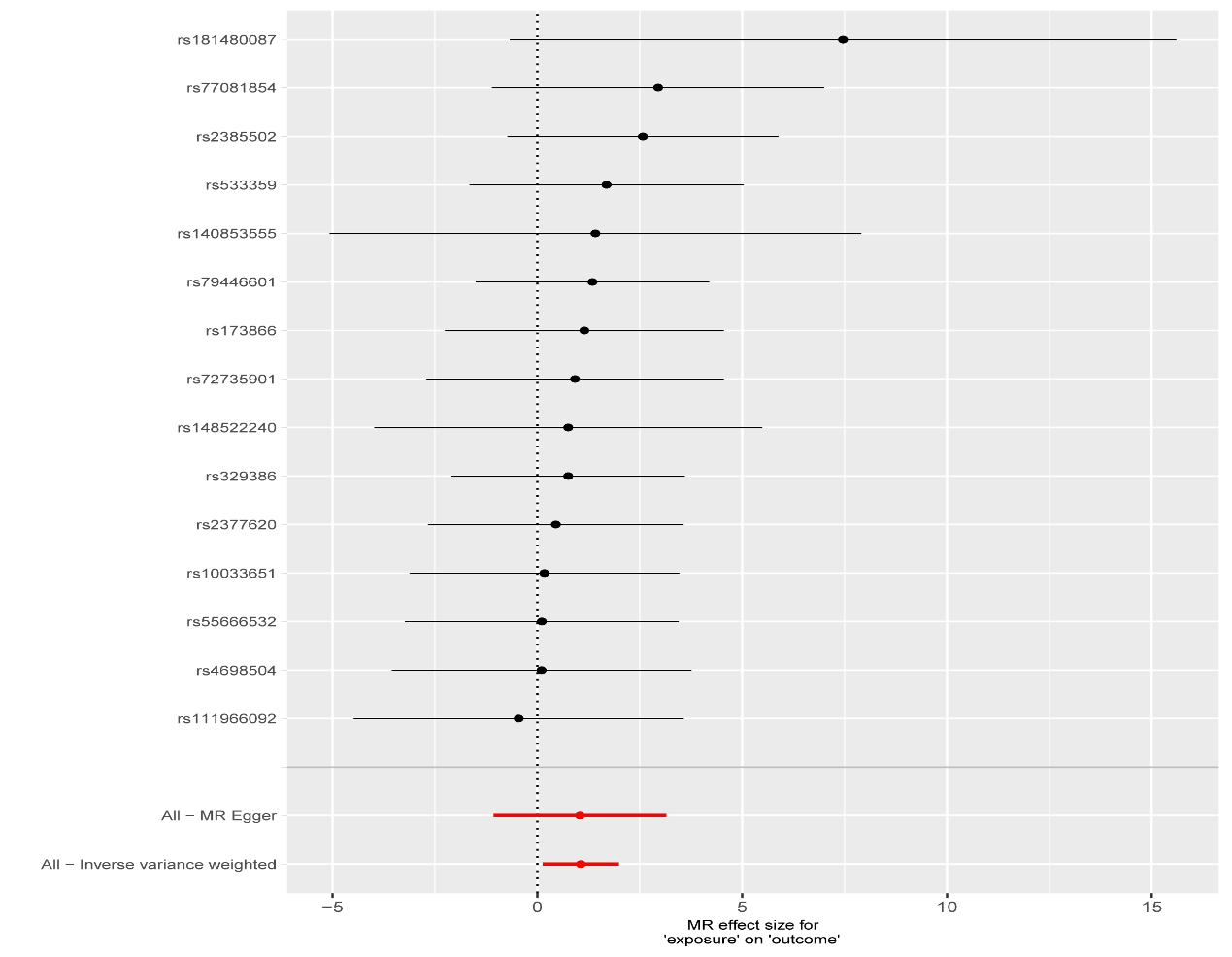

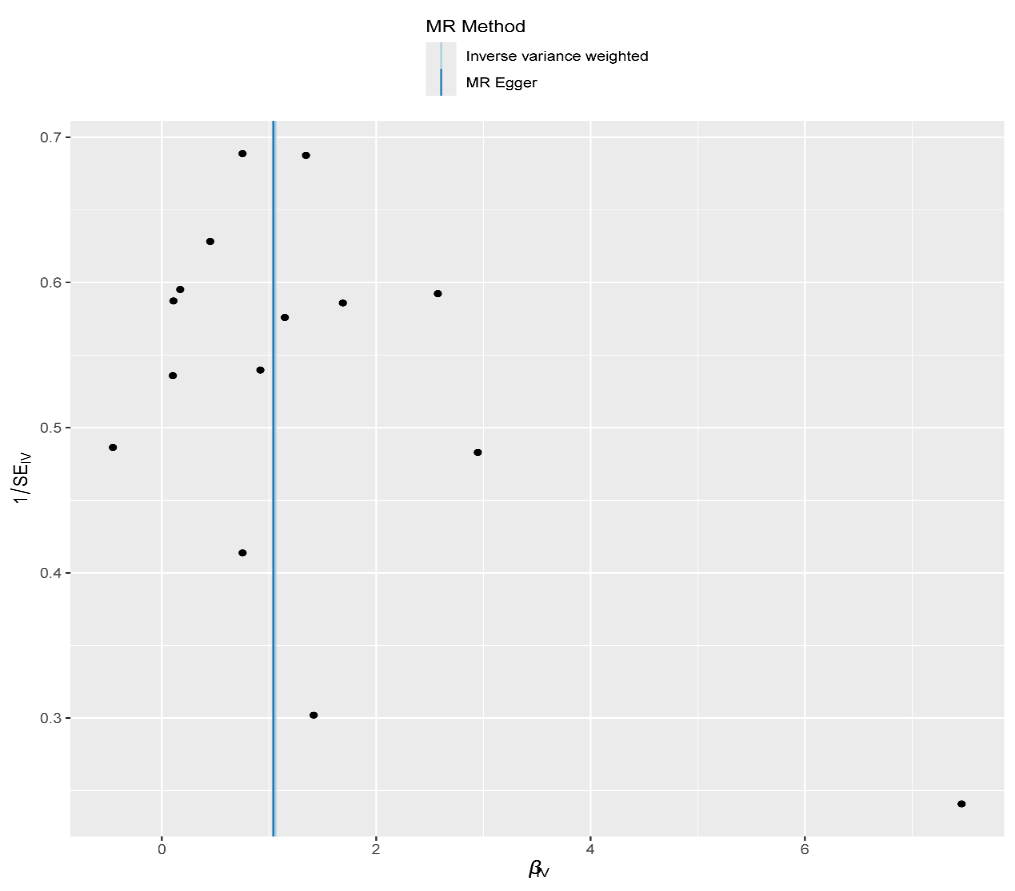


**(A) (B)**


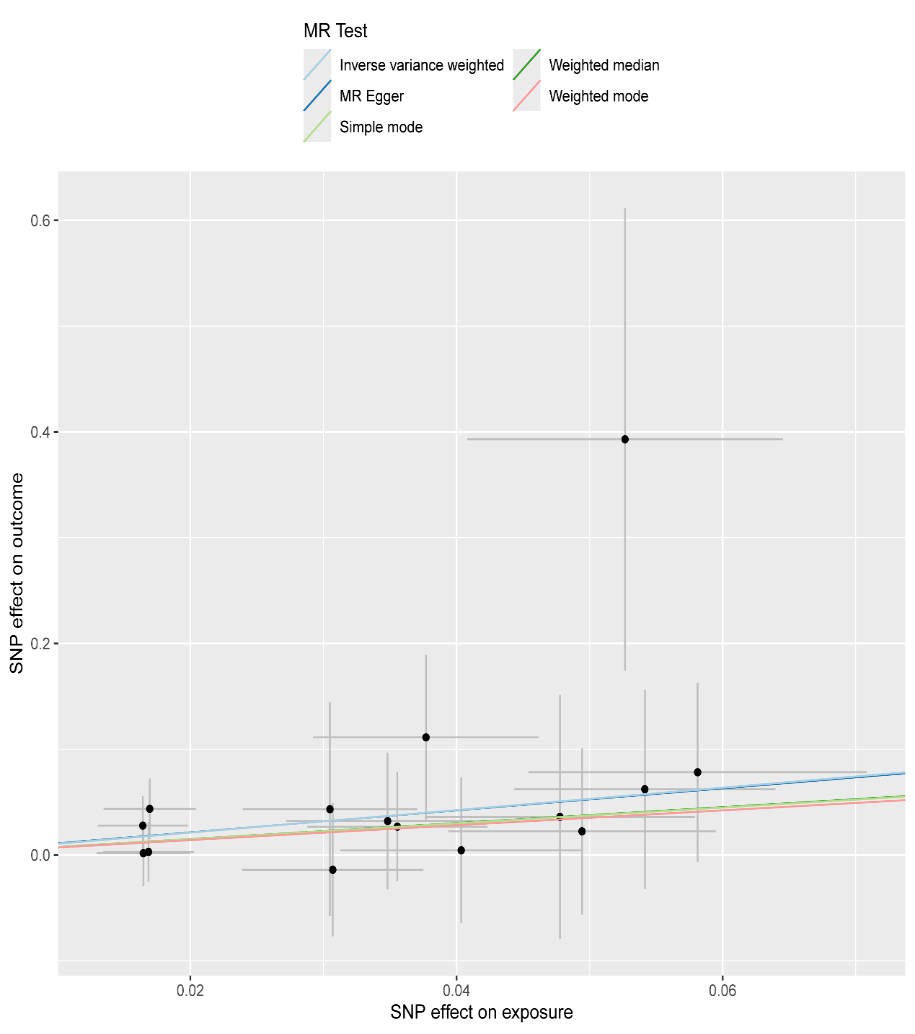

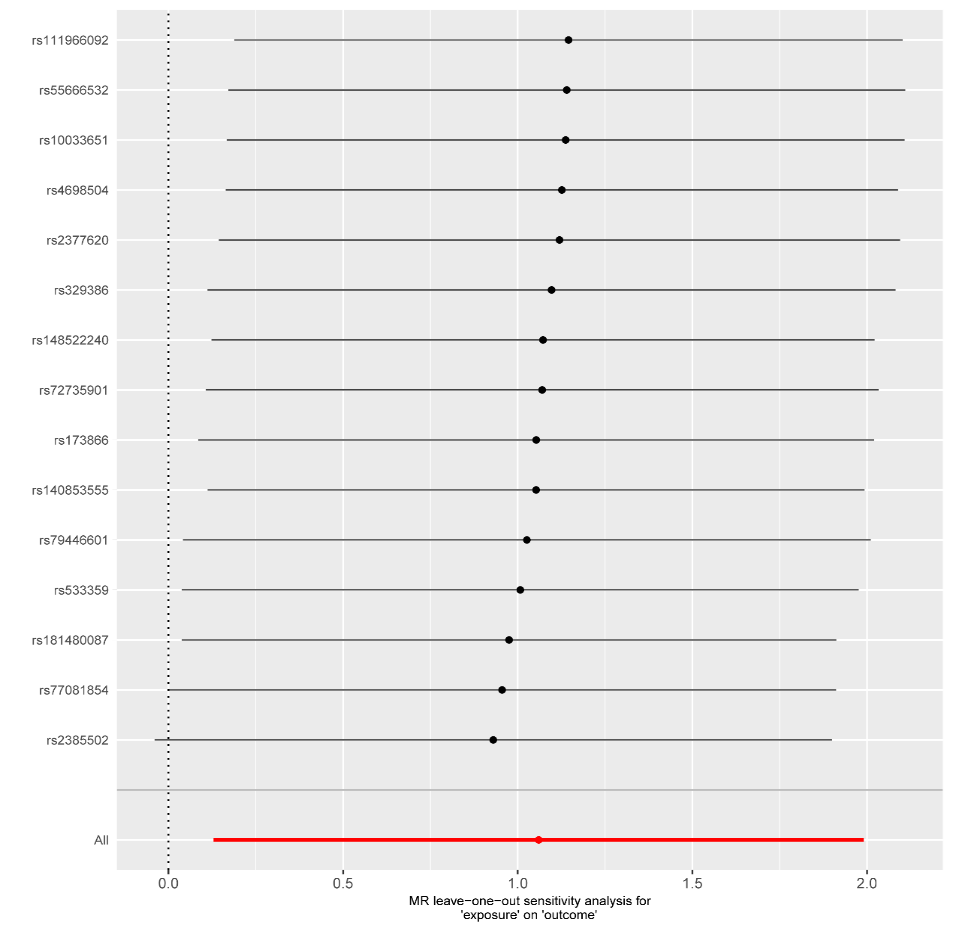


**(C) (D)**

### Thioalkalivibrionaceae on UC.


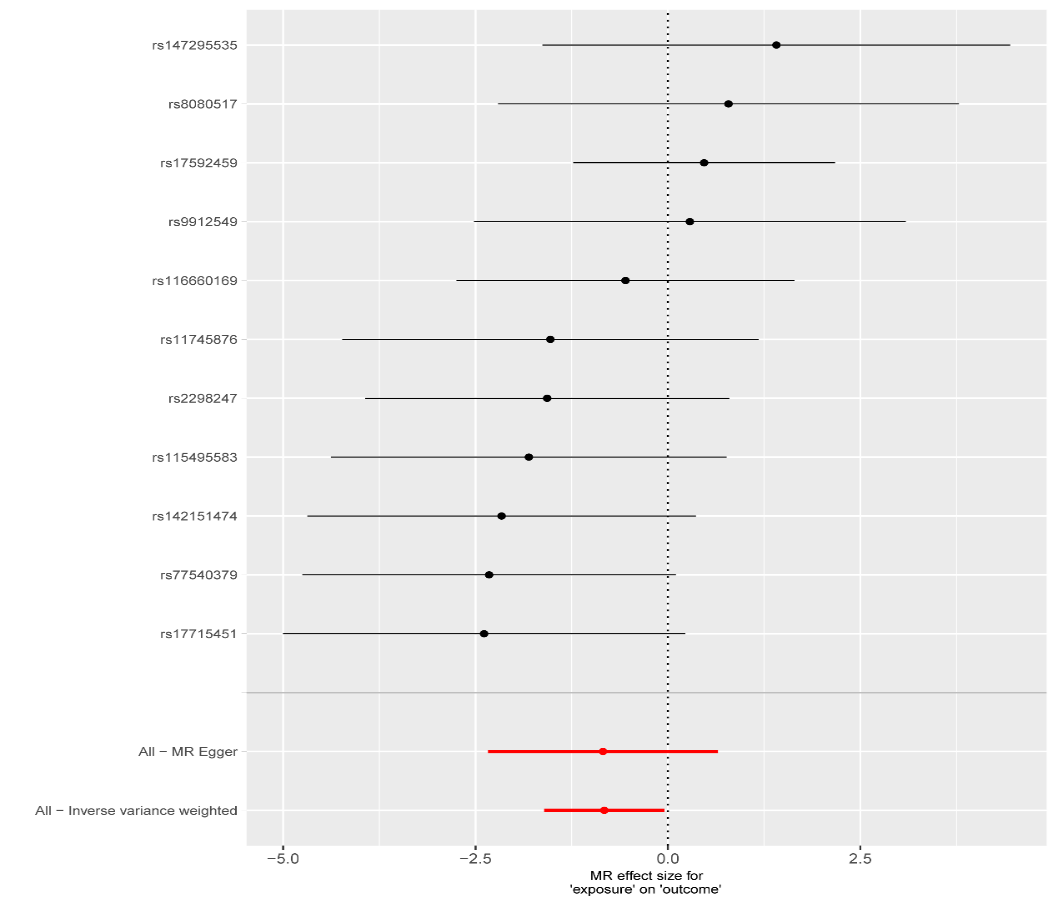

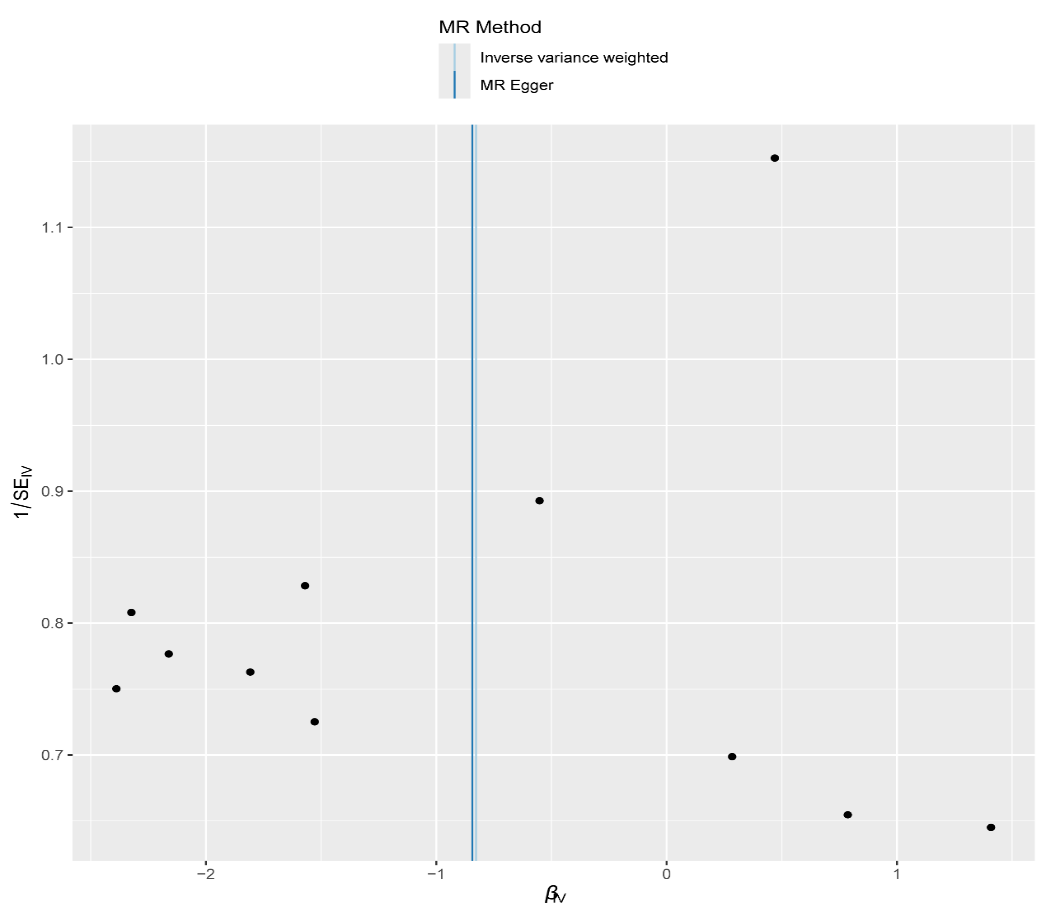


**(A) (B)**


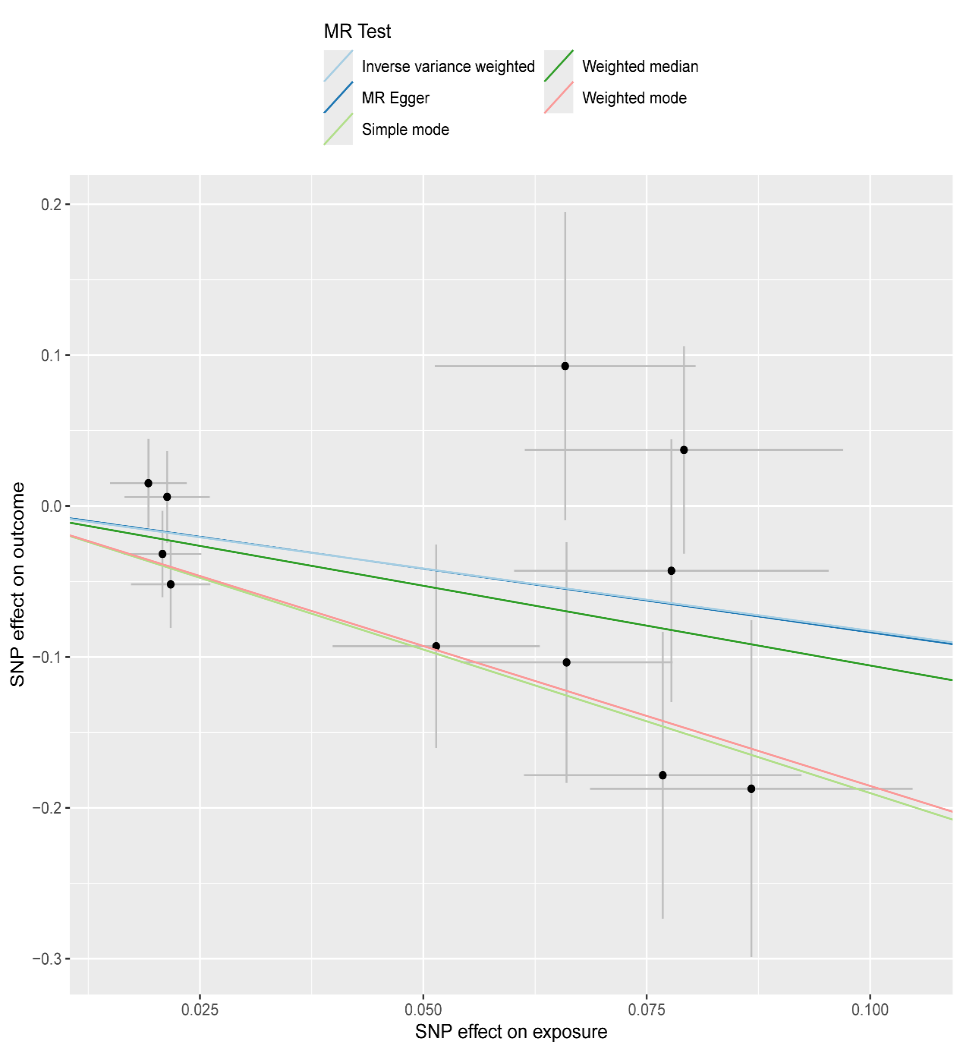

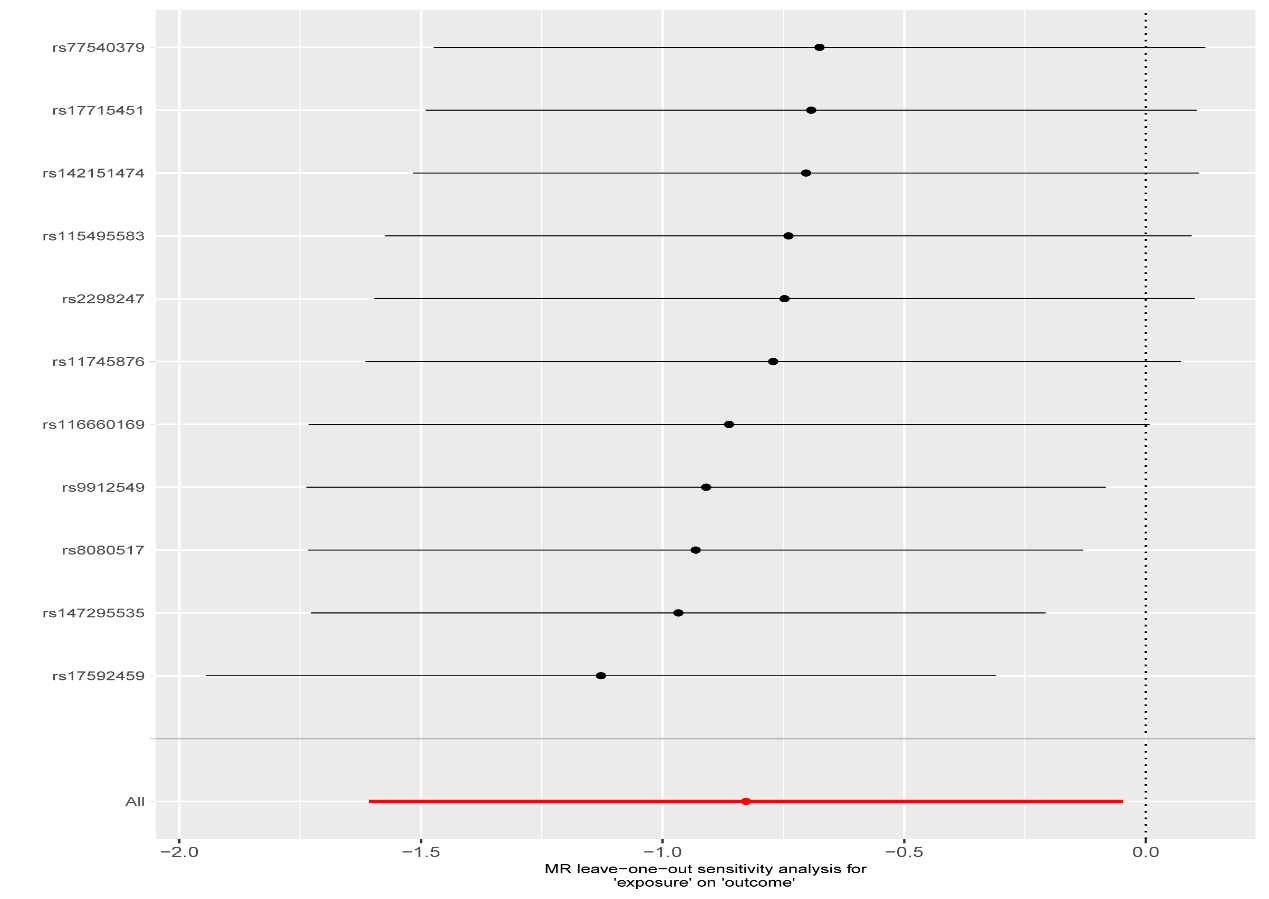


**(C) (D)**

### UBA1066 sp900317515 on UC.


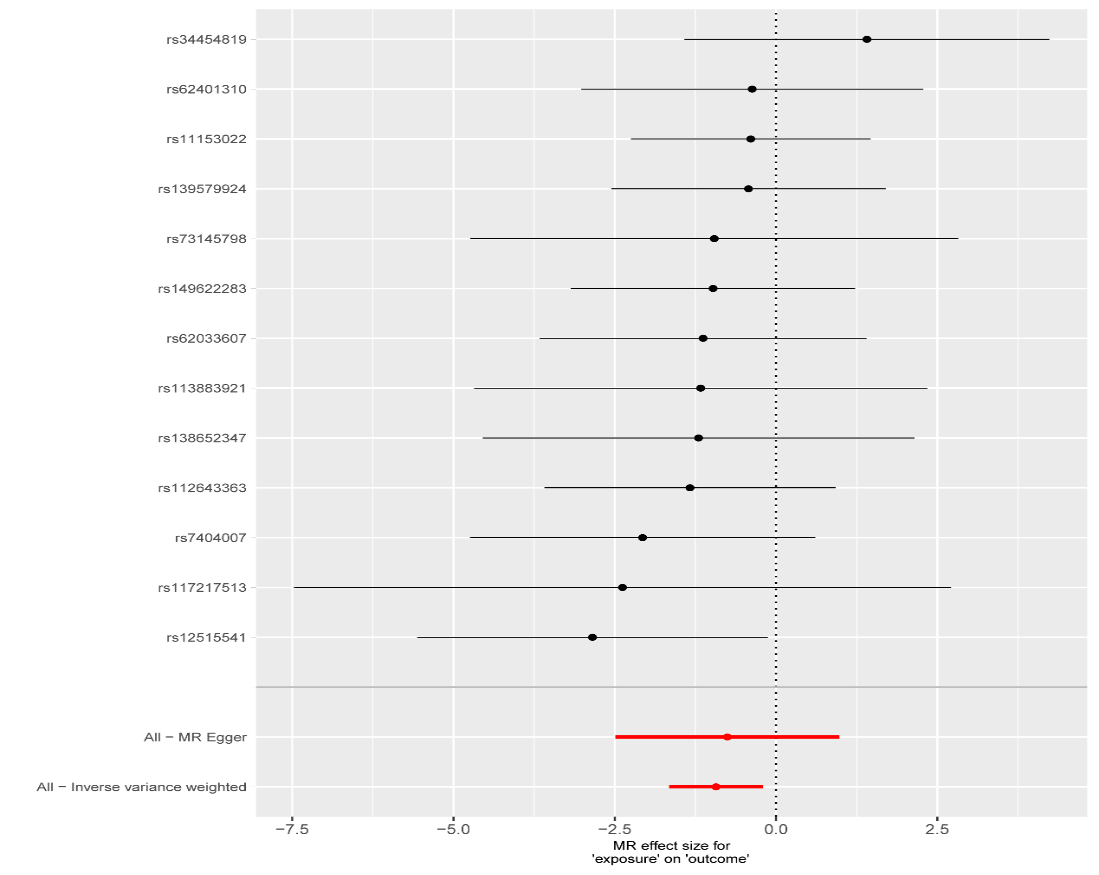

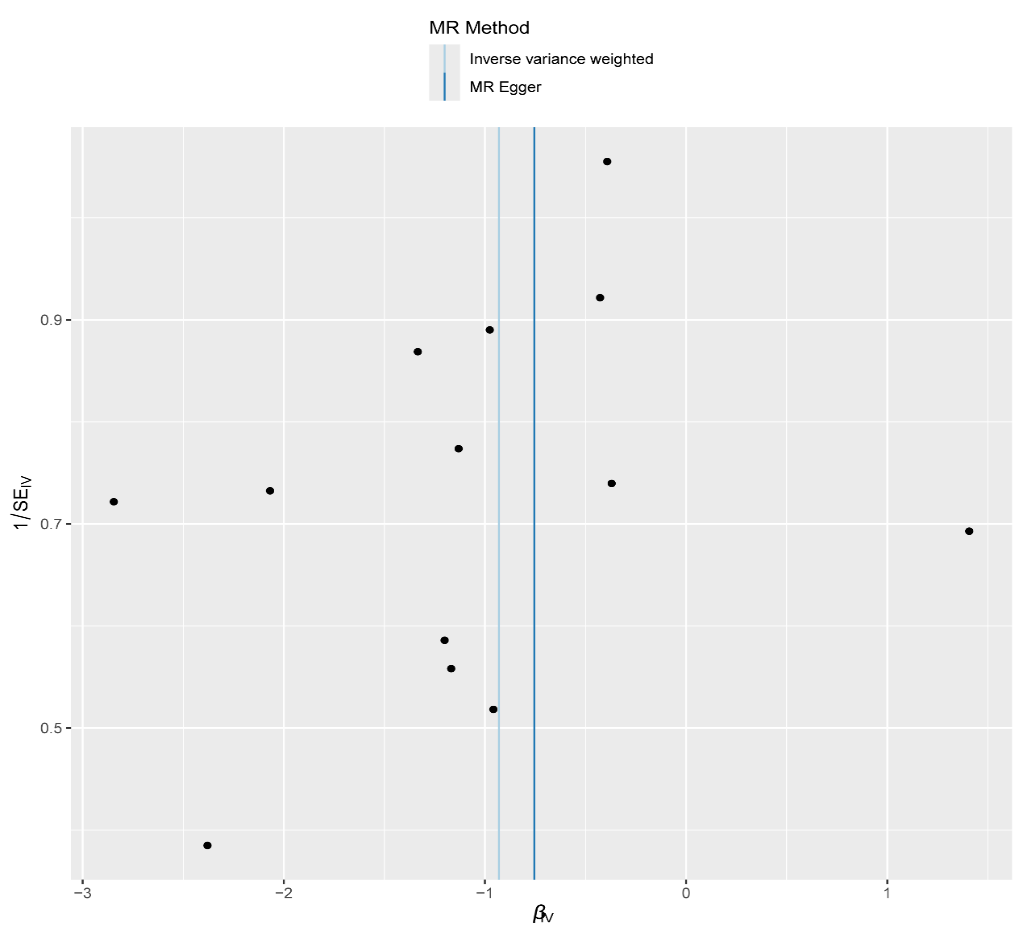


**(A) (B)**


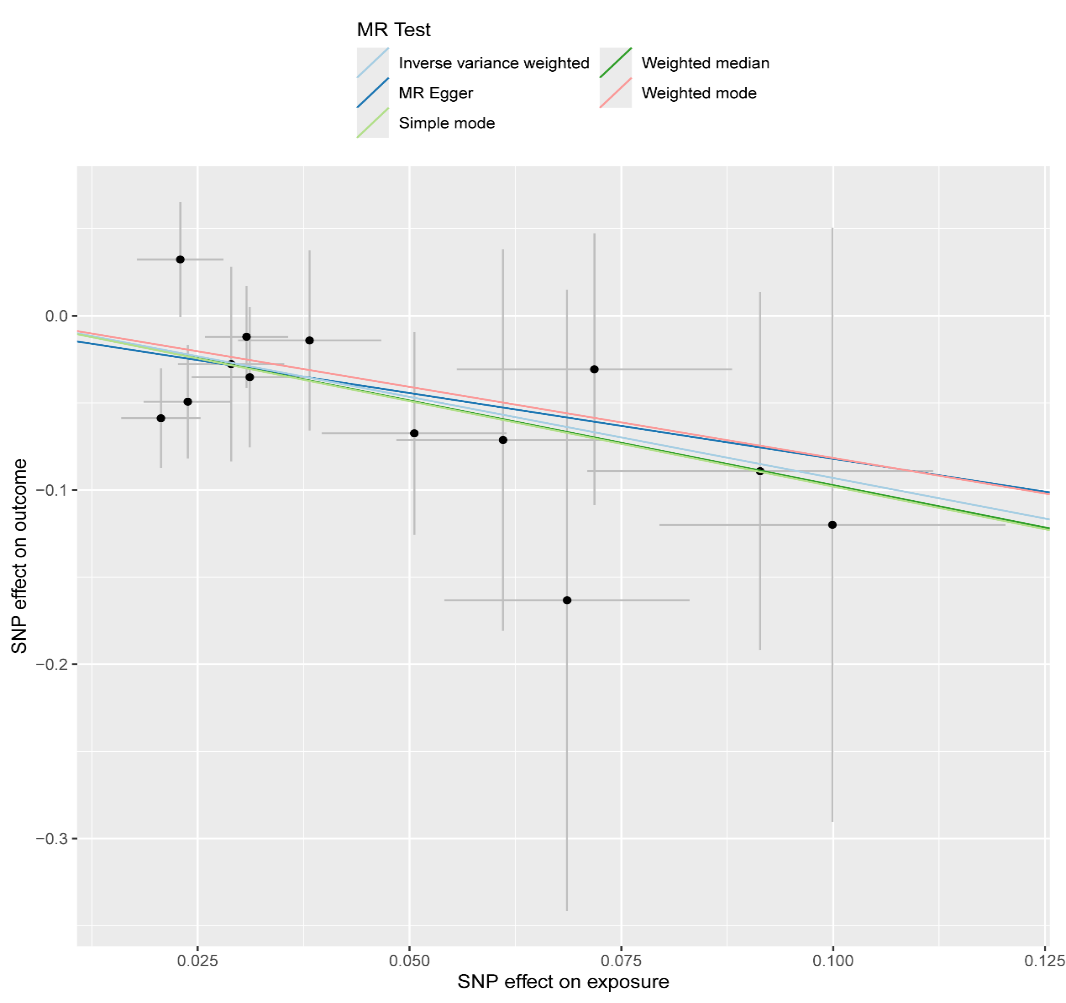

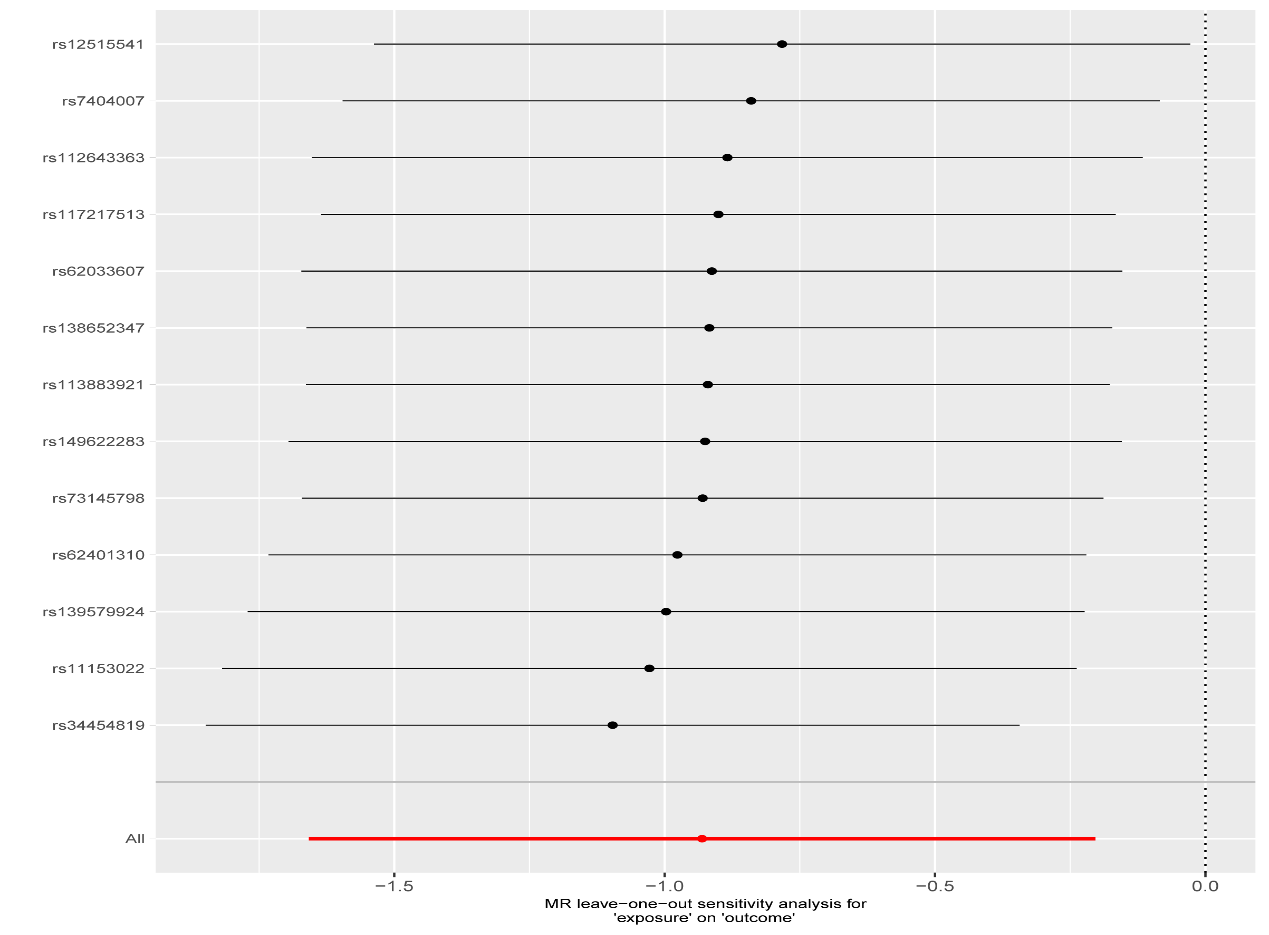


**(C) (D)**

### UBA2922 sp900313925 on UC.


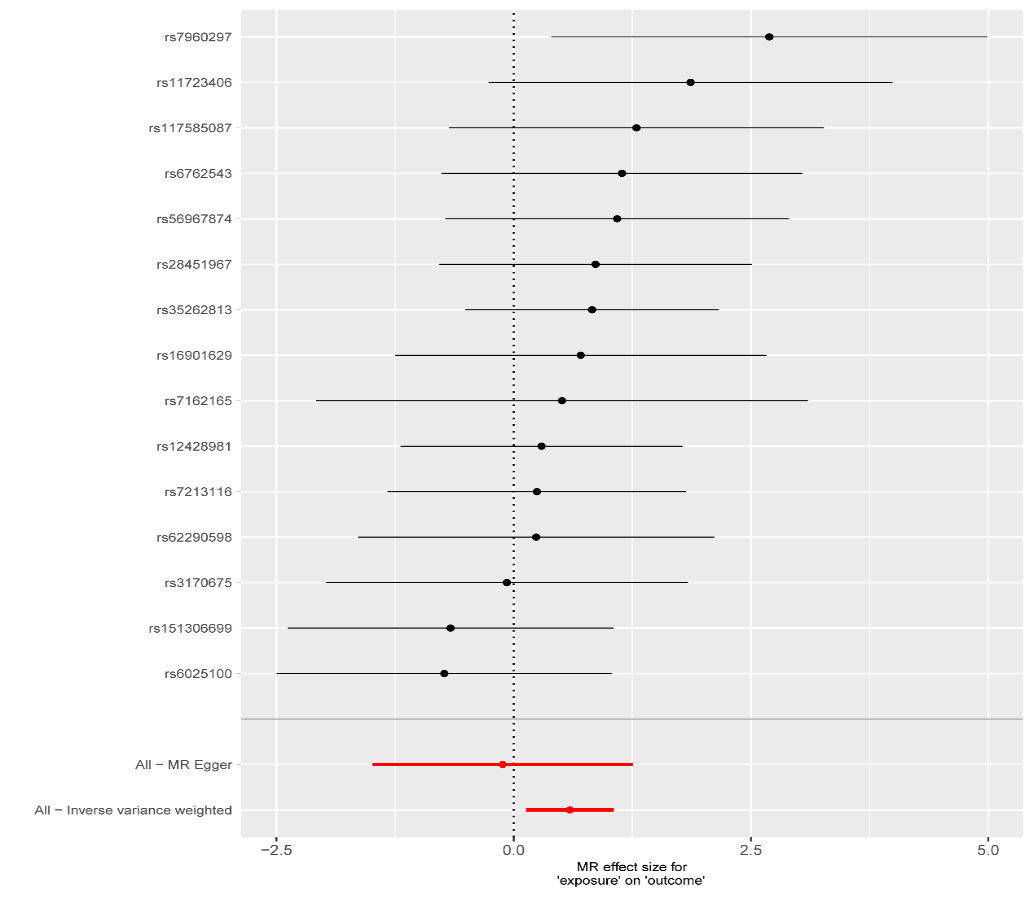

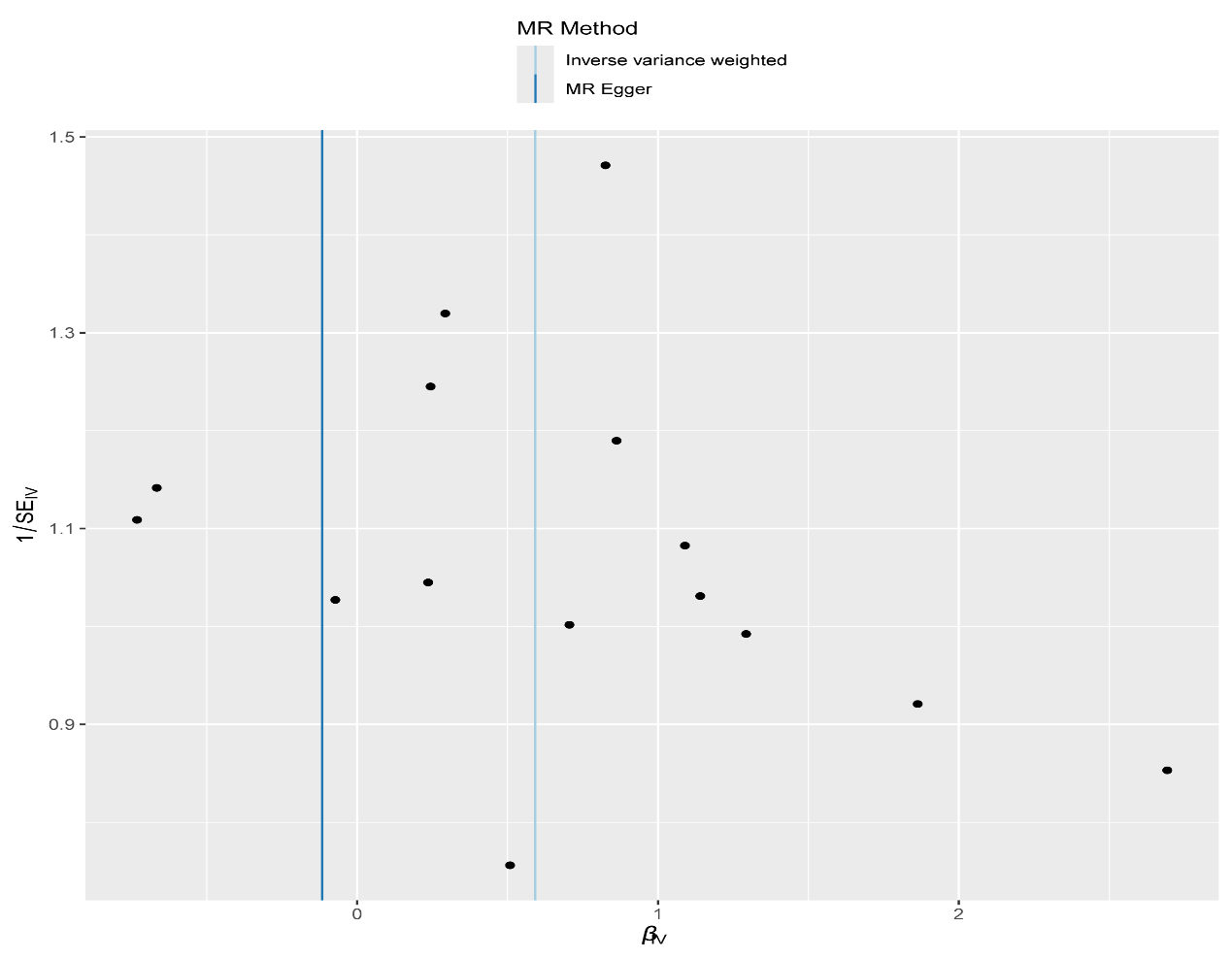


**(A) (B)**


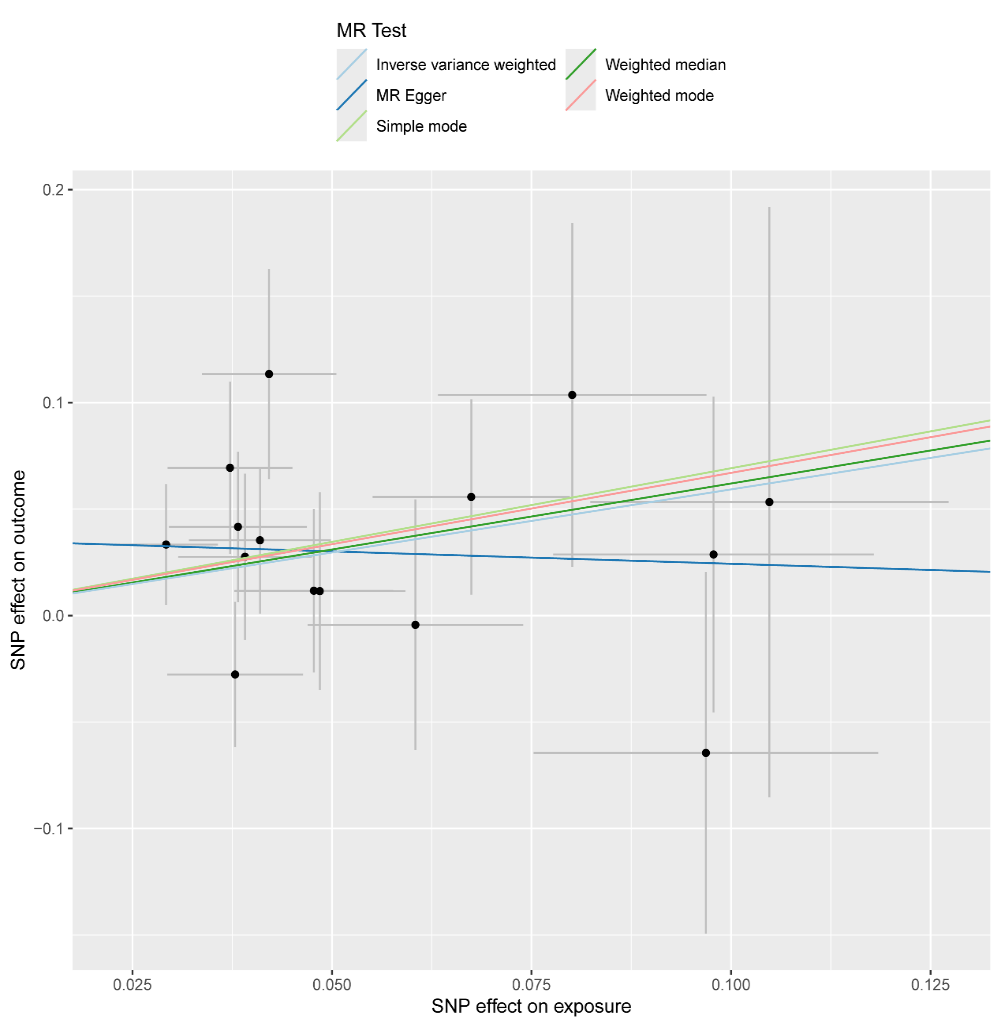

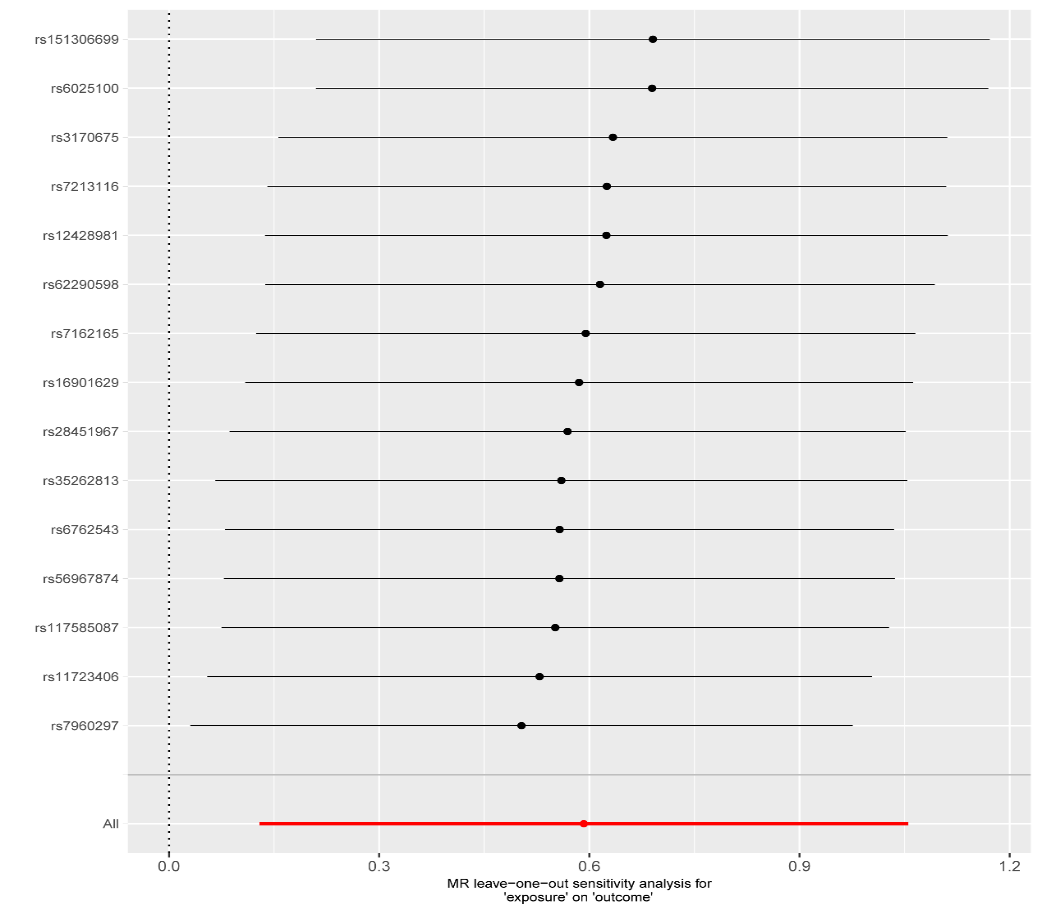


**(C) (D)**
